# BCL‐2‐family protein tBID can act as a BAX‐like effector of apoptosis

**DOI:** 10.15252/embj.2021108690

**Published:** 2021-12-21

**Authors:** Hector Flores‐Romero, Lisa Hohorst, Malina John, Marie‐Christine Albert, Louise E King, Laura Beckmann, Tamas Szabo, Vanessa Hertlein, Xu Luo, Andreas Villunger, Lukas P Frenzel, Hamid Kashkar, Ana J Garcia‐Saez

**Affiliations:** ^1^ Institute for Genetics University of Cologne Cologne Germany; ^2^ Cologne Excellence Cluster on Cellular Stress Responses in Aging‐Associated Diseases (CECAD) University of Cologne Cologne Germany; ^3^ Interfaculty Institute of Biochemistry Eberhard‐Karls‐Universität Tübingen Tübingen Germany; ^4^ Institute for Molecular Immunology, and Center for Molecular Medicine Cologne (CMMC) Faculty of Medicine University Hospital of Cologne University of Cologne Cologne Germany; ^5^ Department I of Internal Medicine University Hospital of Cologne Cologne Germany; ^6^ Center of Integrated Oncology ABCD University Hospital of Cologne Cologne Germany; ^7^ Division of Developmental Immunology Biocenter Medical University of Innsbruck Innsbruck Austria; ^8^ Eppley Institute for Research in Cancer and Allied Diseases Fred & Pamela Buffett Cancer Center University of Nebraska Medical Center Omaha ME USA; ^9^ Department of Pathology and Microbiology University of Nebraska Medical Center Omaha NE USA; ^10^ CeMM Research Center for Molecular Medicine of the Austrian Academy of Sciences Vienna Austria; ^11^ Ludwig Boltzmann Institute for Rare and Undiagnosed Diseases Vienna Austria; ^12^ Present address: Children Cancer Research Institute (CCRI) Vienna Austria

**Keywords:** apoptosis, BCL‐2 proteins, mitochondrial permeabilization, pore formation, Autophagy & Cell Death, Membranes & Trafficking

## Abstract

During apoptosis, the BCL‐2‐family protein tBID promotes mitochondrial permeabilization by activating BAX and BAK and by blocking anti‐apoptotic BCL‐2 members. Here, we report that tBID can also mediate mitochondrial permeabilization by itself, resulting in release of cytochrome c and mitochondrial DNA, caspase activation and apoptosis even in absence of BAX and BAK. This previously unrecognized activity of tBID depends on helix 6, homologous to the pore‐forming regions of BAX and BAK, and can be blocked by pro‐survival BCL‐2 proteins. Importantly, tBID‐mediated mitochondrial permeabilization independent of BAX and BAK is physiologically relevant for SMAC release in the immune response against Shigella infection. Furthermore, it can be exploited to kill leukaemia cells with acquired venetoclax resistance due to lack of active BAX and BAK. Our findings define tBID as an effector of mitochondrial permeabilization in apoptosis and provide a new paradigm for BCL‐2 proteins, with implications for anti‐bacterial immunity and cancer therapy.

## Graphical abstract

Pro‐apoptotic BCL‐2‐family protein tBID can promote mitochondrial permeabilization not just via BAX/BAK activation, but also on its own to mediate antibacterial responses and apoptosis of venetoclax‐resistant leukaemia cells.

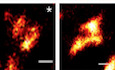

## Introduction

Apoptosis is a form of programmed cell death that allows the immunologically silent clearance of unwanted or malfunctioning cells, for example, during embryonic development or to maintain tissue homeostasis in adult organisms (Singh *et al*, 2019). Permeabilization of the mitochondrial outer membrane (MOM) is a key step in the intrinsic apoptosis pathway, as it defines the point‐of‐no‐return in the cellular commitment to death. It leads to the release into the cytosol of apoptotic factors, such as cytochrome c (cyt c) and Second mitochondria‐derived activator of caspase/direct inhibitor of apoptosis‐binding protein with low pI (SMAC/DIABLO; Zamzami *et al*, 1995; Liu *et al*, 1996), that help to unleash the apoptotic caspase cascade for the organized dismantling of the cell.

The proteins of the BCL‐2 family are major apoptosis regulators that directly control MOM permeabilization (MOMP). The BCL‐2 family members have been further classified into: (i) effectors, such as BAX and BAK, and perhaps BOK, which induce apoptosis by creating the mitochondrial pores responsible for MOMP (Moldoveanu & Czabotar, 2019), (ii) the anti‐apoptotic counterparts, such as BCL‐2, BCLXL or MCL1, which inhibit apoptosis by blocking the pro‐apoptotic family members and play a role in chemotherapeutic resistance (Maji *et al*, 2018) and (iii) the so‐called BH3‐only proteins, including BID and BIM. BH3‐only proteins tune the opposing functions of the effector and the anti‐apoptotic BCL‐2 proteins. They promote apoptosis by activating BAX‐like proteins and/or preventing the anti‐apoptotic proteins from complex formation with them. Small molecule drugs, mimicking their function, i.e., BH3‐mimetics, have entered clinical use to treat cancer.

The BH3‐only protein BID has been shown to link mitochondrial apoptosis with the death receptor‐initiated apoptosis signalling pathway (Li *et al*, 1998; Luo *et al*, 1998). Recently, it has also been implicated in the modulation of the cellular responses to bacterial infection (Masson *et al*, 2011; Andree *et al*, 2014; Hu *et al*, 2017; Heilig *et al*, 2020). BID exists in an inactive form in the cytosol of healthy cells. Upon induction of apoptosis, it is post‐translationally activated through cleavage by caspase‐8, which gives rise to cBID (cleaved BID), composed of the N‐terminal p7 and C‐terminal p15 subunits that remain initially bound to each other. Active cBID then accumulates at the MOM, where the two subunits separate, thereby releasing the N‐terminal part from the cell‐death‐promoting segment (tBID, p15; Gross *et al*, 1999; Bleicken *et al*, 2012). tBID has been shown to promote BAX and BAK activation not only by directly binding to them, but also by releasing them from inhibitory complexes with the anti‐apoptotic BCL‐2‐family members due to its enhanced affinity for these proteins in the membrane (Garcia‐Saez *et al*, 2009; Bleicken *et al*, 2017).

In contrast to other BH3‐only proteins that are largely unstructured, BID is structurally similar to the effector and anti‐apoptotic BCL‐2 proteins. In solution, it also adopts the so‐called BCL‐2 fold, a globular helical 3D structure in which the central, predominantly hydrophobic α‐helix 6 (equivalent to α‐helix 5 in BAX and BAK) is flanked on both sides by pairs of amphipathic helices (Muchmore *et al*, 1996; Chou *et al*, 1999; McDonnell *et al*, 1999; Suzuki *et al*, 2000; Petros *et al*, 2004). In the apoptotic pore formed by BAX and likely BAK, the hairpin of α helices 5–6 contributes to the pore opening by generating membrane stress and by stabilizing the high curvature at the pore edge (Basanez *et al*, 1999; Terrones *et al*, 2004; Garcia‐Saez *et al*, 2005; Fuertes *et al*, 2010). Studies using *in vitro* model systems have reported diverse effects of tBID on membranes, including permeabilization, for which its central α‐helix 6 plays a crucial role (Kudla *et al*, 2000; Esposti *et al*, 2001; Garcia‐Saez *et al*, 2004, 2005). Yet, tBID has been so far considered purely as a regulator of the activity of effector and anti‐apoptotic BCL‐2 proteins, being a major representative of the BH3‐only proteins.

Here, we report the discovery of an additional function of tBID by which it can directly elicit MOMP and apoptosis also when active BAX and BAK are absent. This newly recognized effector‐like function of tBID requires α‐helix 6, but not its BH3 domain, and can be regulated by anti‐apoptotic BCL‐2 proteins. It causes mitochondrial alterations typical of apoptosis, including fragmentation and cristae remodelling, as well as the release of cyt c, SMAC and mitochondrial DNA (mtDNA) into the cytosol. Importantly, we demonstrate that tBID‐mediated MOMP plays a physiological role in SMAC release during *Shigella flexneri* infection and that it has therapeutic potential, as it can be used to kill venetoclax‐resistant leukaemia cells.

## Results

### tBID alone suffices to induce MOMP and apoptosis

The many members of the BCL‐2 family establish a complex interaction network in the cell that dictates its fate. The partially overlapping functions of the different family members make it difficult to dissect the specific roles of the individual members by genetic means. Many of these issues can be excluded by using a cell line lacking all relevant BCL‐2 family proteins (deficient for the ten BH3‐only proteins: BAD, BIK, PUMA, HRK, BMF, BID, BIM, NOXA, BNIP3 and NIX, for five anti‐apoptotic BCL‐2 proteins, including BCL‐2, BCLXL, MCL‐1, BCL‐W and A1, as well as for the effectors BAX and BAK). The resulting HCT116 all BCL‐2 knockout (KO) cells (termed hereafter as HCT AKO) resist all stimuli known to trigger mitochondrial apoptosis (O'Neill *et al*, 2016) and were used here to study the function of tBID. Please note that the BCL‐2 family member BOK, despite being barely detectable in these cells and considered inert to interaction with other family members, has also been removed genetically in some of the key experiments shown below.

**Figure 1:**
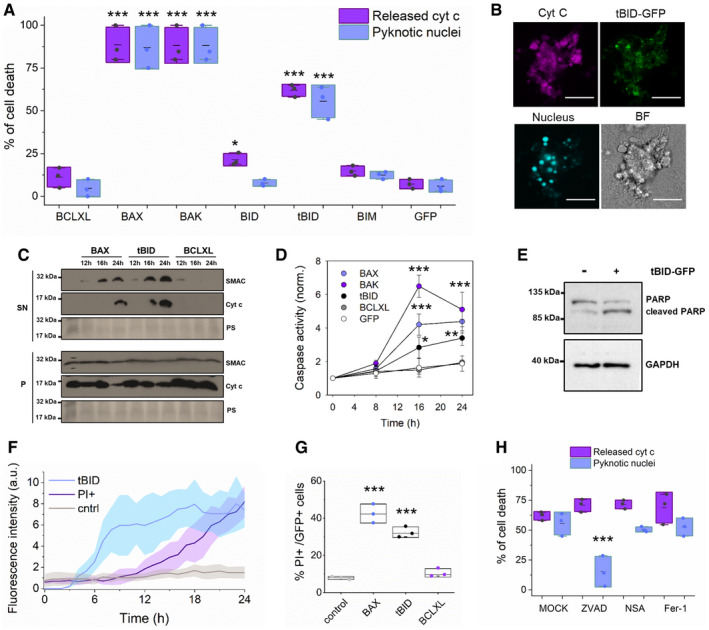
tBID induces MOMP and apoptosis in HCT AKO cells Quantification of the effect of individual BCL‐2‐family proteins on HCT AKO cell death, measured as percentage of cells showing released cyt c or pyknotic nuclei. Data correspond to at least three independent experiments, *n* > 30 cells per condition per experiment. ***P < 0.001 and *P < 0.05 with respect to GFP condition.Representative confocal immunofluorescence and Brightfield (BF) images of an apoptotic HCT AKO cell expressing tBID‐GFP (green), showing cyt c release (magenta), formation of pyknotic nuclei (cyan) and plasma membrane blebs. Scale bar is 10 µm.Western blot analysis of SMAC and cyt c release in GFP‐BAX, tBID‐GFP and GFP‐BCLXL expressing cells at 12, 16 and 24 h after transfection in HCT AKO. SN (supernatant, cytosolic fraction), P (pellet, membrane fraction) and PS (Ponceau Staining).Quantification of caspase activity in HCT AKO cells expressing GFP‐BAX, GFP‐BAK, tBID‐GFP, GFP‐BCLXL and GFP at different time points after transfection. *n* = 3 independent experiments. ***P < 0.001, **P < 0.025 and *P < 0.05 with respect to GFP condition. Error bars represent SD.Representative western blot analysis of PARP1 cleavage upon tBID‐GFP expression in HCT AKO cells. *n* = 3 independent experiments.Kinetics of the expression of tBID‐GFP (blue) and PI intake upon tBID expression (purple) and untransfected HCT116 AKO cells (in grey). Plots show the average fluorescence intensity (dark lines) and its standard deviation (shaded areas) from three technical replicates and three independent experiments. Fluorescence intensity was obtained by analysing the total fluorescence per well using incucyte system every 60 min.Quantification of PI^+^ cells from total transfected cells with GFP‐BAX, tBID‐GFP, GFP‐BCLXL and GFP. *n* = 3 independent experiments with > 10,000 cells per condition per experiment. ***P < 0.001 with respect to untransfected condition.Cell death induced by tBID‐GFP in HCT AKO cells in the absence and presence of ZVAD, NSA and Ferrostatin‐1. Data correspond to three independent experiments, *n* > 30 cells per condition per experiment. ***P < 0.001 with respect to MOCK condition. Data information: In (A), (G) and (H), dots correspond to independent experiments, boxes represent 96% confidence interval, the average is represented by the line inside the box and whiskers correspond to SD. The statistical significance was assessed by one‐way analysis of variance (ANOVA). Source data are available online for this figure.

**Figure EV1:**
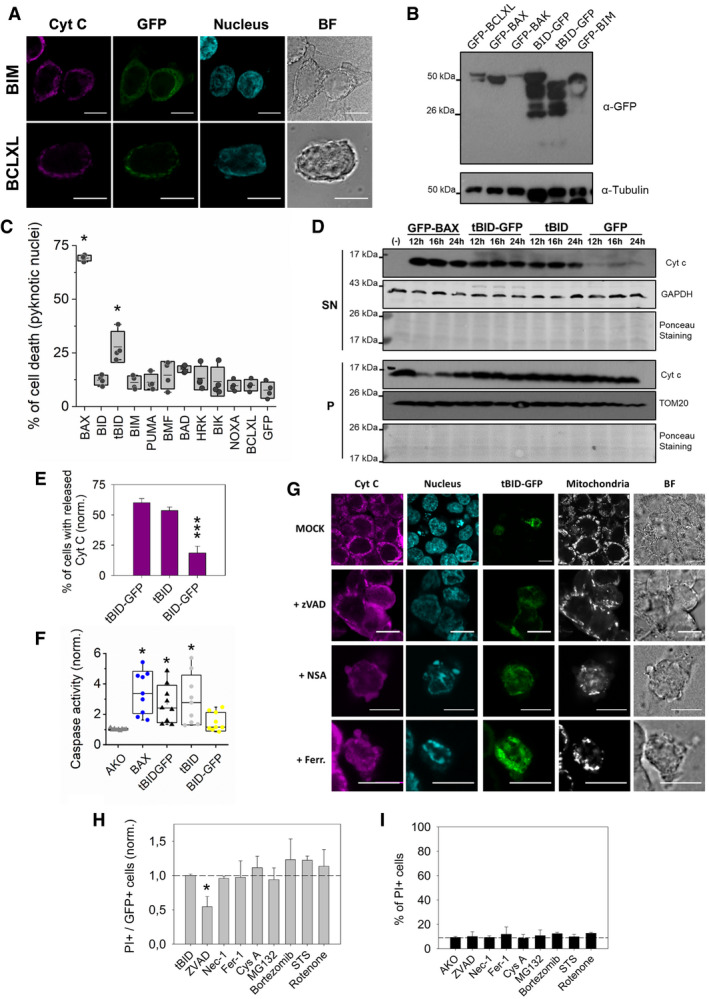
tBID, but not other BH3‐only proteins or BCLXL, induces apoptosis in HCT AKO cells ARepresentative confocal immunofluorescence and BF images of GFP‐BIM and GFP‐BCLXL expressed in HCT116 AKO cells showing mitochondrial cytochrome c and cellular nuclei. Scale bar is 10 µm.BRepresentative western blot of the expression levels of GFP‐tagged BCL2 proteins in HCT AKO cells after 18 h after transfection.CPercentage of cell death by quantification of pyknotic nuclei formation in HCT AKO cells expressing GFP‐tagged BAX, BID, tBID, BIM, PUMA, BMF, BAD, HRK, BIK, NOXA, BCLXL and GFP. *n* = 4 independent experiments with > 50 cells per condition per experiment. *P < 0.05 with respect to GFP condition.DWestern blot of subcellular localization of cyt c of HCT AKO cells expressing GFP‐BAX, tBID‐GFP and non‐tagged tBID.EQuantification of cyt c release in HCT AKO cells expressing tBID‐GFP, tBID and BID‐GFP. *n* = 3 independent experiments with > 20 cells per condition per experiment. Unpaired Student’s *t*‐test ***P < 0.001 with respect to tBID‐GFP condition. Error bars represent SD.FCaspase3/7 activity induced by GFP‐BCL‐2 proteins overexpression in HCT AKO cells normalized to untransfected condition. Each dot represents one technical replicate from *n* = 3 independent experiments *P < 0.05 with respect to untransfected condition.GRepresentative confocal immunofluorescence and BF images of HCT AKO cells expressing tBID‐GFP, and in the presence of ZVAD, NSA and Ferrostatin‐1 (Fer‐1). Scale bar is 10 µm.H, IEffect of different cell death‐related drugs upon incubation on HCT AKO cells transfected with tBID‐GFP, measured as PI^+^ cells. For zVAD, Nec‐1 and Fer‐1 treatment was added 6–8 h after transfection and monitored for additional 6–8 h, whereas for Cys A, MG132, Bortezomib, STS and rotenone, were incubated for 3–4 h after 16–18 h transfection. With > 10,000 cells per condition per experiment. *P < 0.05. Error bars represent SD. Data information: Unless otherwise stated, in C and F, dots correspond to independent experiments, boxes represent 96% confidence interval, the average is represented by the line inside the box and whiskers correspond to SD. and the statistical significance was assessed by one‐way analysis of variance (ANOVA). Source data are available online for this figure.

In order to compare the ability of different GFP‐tagged BCL‐2 proteins to induce apoptosis, we ectopically expressed them tagged with GFP in the HCT AKO cells and visualized MOMP and the cellular pyknotic nuclear phenotype by confocal microscopy. As expected, overexpression of the canonical apoptotic effectors BAX and BAK, but not anti‐apoptotic BCLXL, efficiently induced cell death (Figs 1A and EV1A and B). To our surprise, however, expression of tBID, but not its full‐length counterpart, was also able to induce cyt c and SMAC release from mitochondria. This was accompanied by plasma membrane blebbing and the formation of pyknotic nuclei, all of them well‐recognized hallmarks of apoptosis (Fig 1A–C). This activity was not shared by other activator BH3‐only proteins (Figs 1A and EV1A–C). These results strongly suggest that tBID, in contrast to our current understanding, might be able to directly induce apoptosis independently of BAX and BAK.

To further evaluate this hypothesis, we measured the capacity of tBID‐GFP to induce other hallmarks of apoptotic signalling downstream of MOMP in HCT AKO cells. Despite being somewhat less potent than GFP‐BAX or GFP‐BAK, we found that tBID‐GFP robustly promoted caspase activation (Fig 1D), PARP1 cleavage (Fig 1E) and plasma membrane disruption due to secondary necrosis (Fig 1F and G). As a negative control, GFP‐BCLXL did not induce these effects. Importantly, reconstitution with non‐tagged tBID also induced cyt c release (Fig EV1D and E) and the activation of caspases (Fig EV1F), showing that the C‐terminal GFP‐tag was not significantly contributing to or altering the killing activity of tBID. Along the same lines, expression of untagged tBID using an IRES‐GFP construct yielded the same results (Appendix Fig S1A–E).

To further demonstrate that tBID induced apoptosis and not other cell death pathways, we tested different cell death inhibitors: ZVAD, necrosulfonamide (NSA) and necrostatin‐1 (Nec‐1) and ferrostatin‐1 (Fer‐1), which block apoptosis, necroptosis and ferroptosis respectively (Doll & Conrad, 2017; Cao & Mu, 2020). While NSA, Nec‐1 and Fer‐1 did not affect tBID‐induced cell death, ZVAD significantly inhibited pyknotic nuclei formation, but not cyt c release, indicating that ZVAD impaired cell death at the caspase level downstream of MOMP (Figs 1H and EV1G). Moreover, addition of Cyclosporin A, an inhibitor of the mitochondria permeability transition pore, did not show any significant effect (Fig EV1H and I). Mitochondrial and ER stress triggered by staurosporine (STS), rotenone or the proteasomal inhibitor bortezomib and MG132 did not contribute either to tBID activity, at the times and concentrations tested (Fig EV1H and I).

**Figure EV2:**
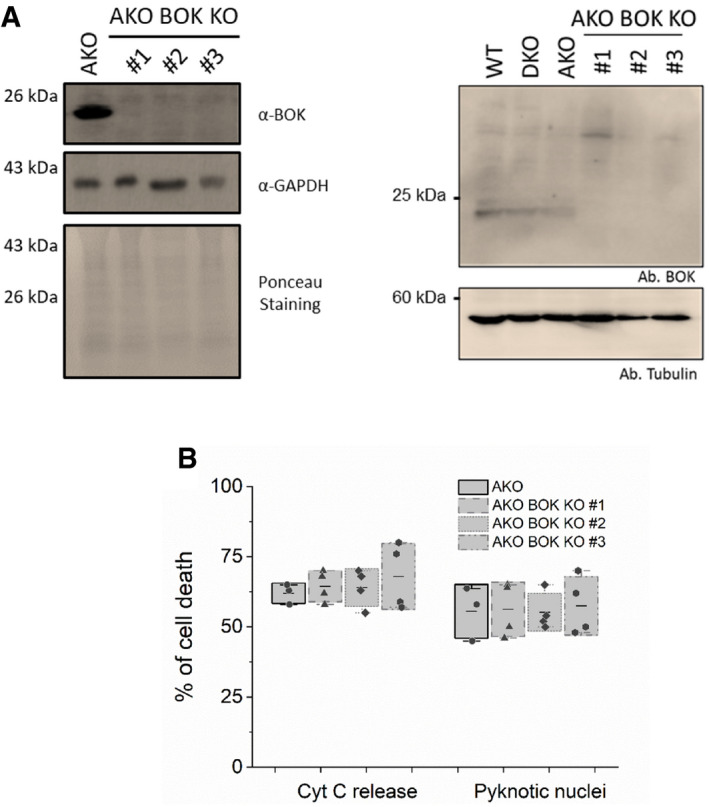
Endogenous BOK is not required for the activity of tBID to induce MOMP Western blot showing the endogenous levels of BOK in HCT wt, DKO, AKO and in three different HCT AKO BOK KO cell lines, with 120 µg (left) and 40 µg (right) per cellular extract.Quantification of the effect of tBID‐GFP on cell death, measured as percentage of cells showing released cytochrome c or pyknotic nuclei. Data correspond to at least three independent experiments, *n* > 30 cells per condition per experiment. Dots correspond to independent experiments, boxes represent 96% confidence interval, the average is represented by the line inside the box and whiskers correspond to SD. The statistical significance was assessed by one‐way analysis of variance (ANOVA). Source data are available online for this figure.

To determine whether tBID‐induced apoptosis in the HCT AKO cells could be mediated via activation of BOK, a BCL‐2 protein that has been reported to be able to directly mediate MOMP (Llambi *et al*, 2016; Fernandez‐Marrero *et al*, 2017; Shalaby *et al*, 2020), we generated a HCT AKO cell line devoid of BOK by CRISPR/Cas9‐mediated deletion (Fig EV2A). Importantly, genetic ablation of BOK did not affect tBID‐induced cell death (Fig EV2B). These findings collectively indicate that tBID is capable of inducing MOMP and apoptosis when overexpressed in HCT AKO cells, independently of the BCL‐2 effectors BAX and BAK, as well as of BOK.

### Endogenous BID can induce apoptosis independently of BAX and BAK

**Figure 2:**
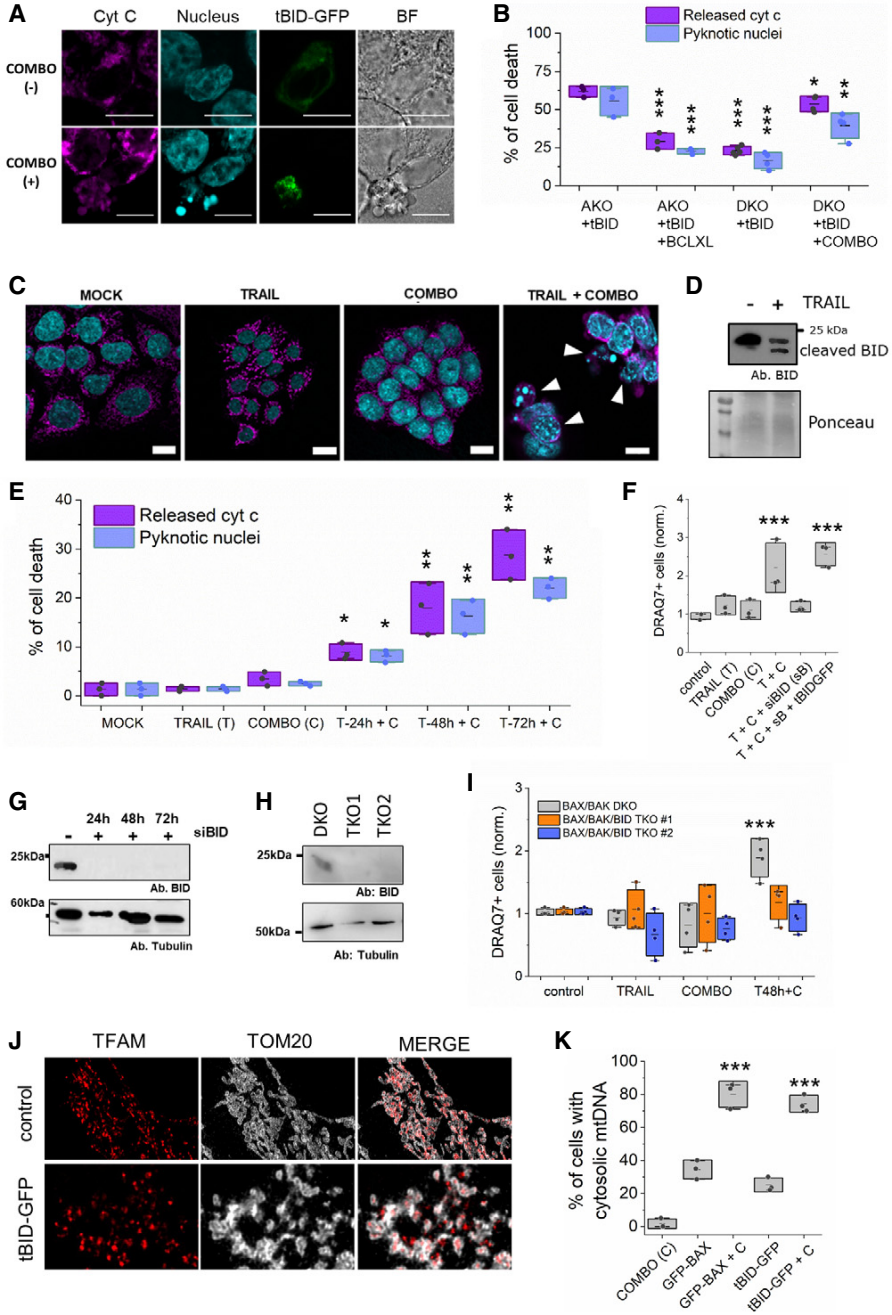
Endogenous BID induces apoptosis independently of BAX and BAK ARepresentative confocal immunofluorescence and bright field (BF) images of HCT DKO cells expressing tBID‐GFP (in green) treated or not with COMBO (ABT737 + S63586). Cyt c shown in magenta and nuclei in cyan. Scale bar is 10 µm.BEffect of tBID‐GFP and mCherry‐BCLXL on cell death, measured as percentage of cells showing released cyt c or pyknotic nuclei. Data correspond to at least three independent experiments, *n* > 30 cells per condition per experiment. ***P < 0.001, **P < 0.025 and *P < 0.05 with respect to tBID‐GFP in HCT AKO condition.CRepresentative confocal immunofluorescence images of HCT DKO cells upon indicated treatments. Nuclei (cyan) and cyt c (magenta) are shown. Scale bar is 10 µm. Arrowheads represent cells showing released cytochrome c and pyknotic nuclei.DRepresentative WB showing the effect of TRAIL stimulation on endogenous BID cleavage in HCT DKO cells. *n* = 3 independent experiments.EPercentage of cell death induced by TRAIL (1 µg/ml), COMBO and their combination at different treatment durations on HCT DKO measured as in panel B. Paired Student’s *t*‐test **P < 0.025 and *P < 0.05 with respect to MOCK condition.FEffect of BID silencing on cell death upon TRAIL/COMBO treatments, measured as DRAQ7^+^ and normalized to siRNA control cell death. *n* = 3 independent experiments. ***P < 0.001 with respect to control condition.G, HRepresentative WB of BID levels after silencing at different time points (G) or when genetically knocked out (H). G, *n* = 3 independent experiments.IEffect of TRAIL (1 µg/ml), COMBO and their combination on HCT DKO and TKO cell death at different time points normalized to control cells. Each dot corresponds to the average of three technical replicates from four independent experiments. ***P < 0.001.JRepresentative HyVolution–super resolution images of TFAM‐RFP expressing U2OS BAX/BAK DKO cells in the absence (top) and presence of tBID‐GFP (bottom). TFAM appears in red, TOM20/mitochondria in grey and tBID‐GFP in green.KQuantification of cells with TFAM release on U2OS BAX/BAK DKO cells transfected with GFP‐BAX/tBID‐GFP and TFAM‐RFP in the presence/absence of COMBO. Dots correspond to individual experiments (*n* = 3 individual experiments with more than 10 cells per condition per experiment). Unpaired Student’s *t*‐test ***P < 0.001 with respect to non‐treated conditions. Data information: Unless otherwise stated, in B, E, F, G, I and K dots correspond to independent experiments, boxes represent 96% confidence interval, the average is represented by the line inside the box and whiskers correspond to SD and the statistical significance was assessed by one‐way analysis of variance (ANOVA). (T, TRAIL; C, COMBO). Source data are available online for this figure.

In agreement with previous knowledge, we found that the killing activity of overexpressed tBID‐GFP was impaired in HCT116 cells lacking solely BAX and BAK (hereafter defined as HCT DKO), but maintaining the BCL‐2 anti‐apoptotic members that can block tBID‐GFP (Fig 2A and B). Along the same line, co‐expression of BCLXL with tBID in HCT AKO drastically decreased tBID‐induced cell death (Fig 2B). Importantly, we could revert the inhibitory effect of the anti‐apoptotic BCL‐2‐family members over tBID‐induced cell death by addition of ABT‐737, an inhibitor of BCLXL, BCL‐2 and BCLW, combined with the MCL1 inhibitor S63845 (Fig 2A and B), hereafter referred to as “COMBO” treatment.

**Figure EV3:**
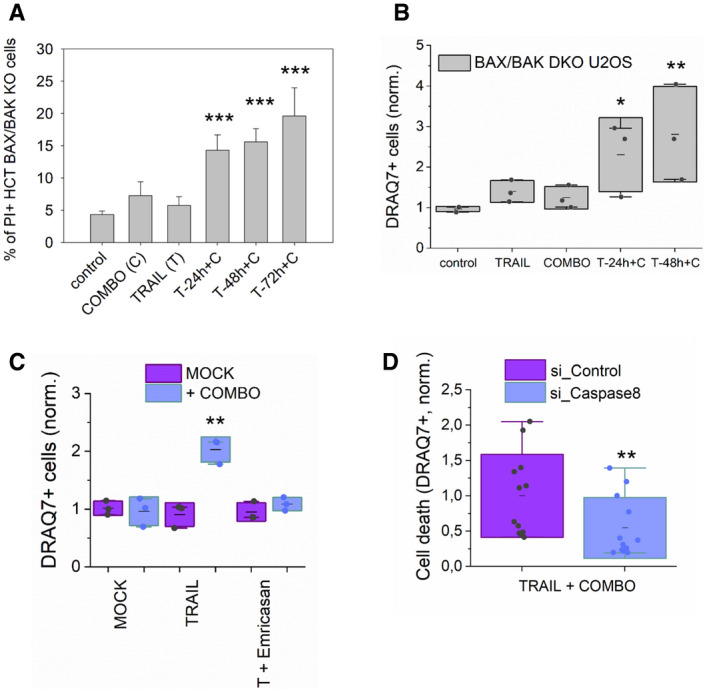
Cell death induced by combination treatment of TRAIL and COMBO requires BID cleavage AEffect of TRAIL (1 µg/ml), COMBO and their combination on HCT DKO transfected, measured as PI^+^ cells. *n* = 4 independent experiments with over 10,000 cells per condition per experiment. ***P < 0.001 with respect to untreated conditions. Error bars represent SD.B, CCell death induced by TRAIL, COMBO and their combination in U2OS DKO (B) and HCT DKO (C), measured as DRAQ7^+^ cells and normalized to untreated cells. *P < 0.05 and **P < 0.025 with respect to control condition.DCell death induced by TRAIL and COMBO combination in HCT DKO transfected with siRNA control or siRNA for Caspase‐8, measured as DRAQ7^+^ cells and normalized to siControl transfected cells. **P < 0.025 with respect to control condition. Unpaired Student’s *t*‐test. Data information: In B, C and D, dots correspond to the average value of three technical replicates of three independent experiments. Boxes represent 96% confidence interval, the average is represented by the line inside the box and whiskers correspond to SD and the statistical significance was assessed by one‐way analysis of variance (ANOVA).

To examine whether endogenous levels of tBID were sufficient to trigger MOMP and apoptosis, we stimulated HCT DKO cells with the death receptor ligand TRAIL, which promotes caspase‐8 activation and cleavage of endogenous BID into tBID. We additionally treated the cells with COMBO to circumvent tBID inhibition by the pro‐survival BCL‐2 proteins in the HCT DKO cells. As expected, TRAIL treatment alone effectively induced BID cleavage in HCT DKO cells (Fig 2D), but did not lead to cell death (Fig 2C and E). Yet, remarkably, we observed a synergistic apoptosis‐inducing effect when COMBO was added to HCT DKO cells pre‐treated with TRAIL (Figs 2C and E, and EV3A). We obtained similar results in U2OS BAK/BAK DKO cells (Fig EV3B), excluding a cell‐line‐specific phenomenon.

Importantly, COMBO stimulation alone did not induce cell death in any of the BAX/BAK DKO cell lines tested (Figs 2E and EV3A and B). Furthermore, treatment with emricasan, a caspase‐8 inhibitor, or knock‐down of caspase‐8, significantly reduced the synergistic effect of TRAIL/COMBO treatment in HCT DKO cells, measured as the decrease on DRAQ7‐positive cells (Fig EV3C and D). This shows a role for caspase‐8 on the process, either by inhibiting BID cleavage, a feedback loop between the different caspases, or both.

To further prove that it is endogenous truncated BID that specifically mediates apoptosis in these settings, we knocked down (KD) by siRNA and knocked out (KO) BID by CRISPR/Cas9 in HCT DKO cells (Fig 2G and H) to generate triple knockout HCT TKO cells. In support of our hypothesis, depletion of BID by either means significantly reduced the lethality of the TRAIL/COMBO treatment in these cells (Fig 2F and I), which could be rescued by ectopically expressed tBID‐GFP (Fig 2F).

Next, as tBID seems to be able to induce MOMP, we asked if it was also able to induce mitochondrial inner membrane permeabilization (MIMP) and mtDNA release, as recently reported for the canonical effectors BAX and BAK (McArthur *et al*, 2018; Riley *et al*, 2018). To address this question, we co‐expressed tBID‐GFP together with the mtDNA marker TFAM‐RED in U2OS BAX/BAK DKO cells (Fig 2J and Appendix Fig S2A and B). By using HyVolution–superresolution imaging, we observed that under non‐apoptotic conditions, neither BAX nor tBID were able to induce mtDNA release (Fig 2K). However, upon inhibition of the anti‐apoptotic proteins with COMBO treatment, both BAX and tBID were able to induce mitochondrial fragmentation and TFAM translocation from the mitochondria into the cytosol (Fig 2K). These results indicate that tBID can execute also this recently recognized function of the canonical effectors BAX and BAK.

### tBID induces apoptosis independently of its BH3 domain but requires an intact helix 6

**Figure 3:**
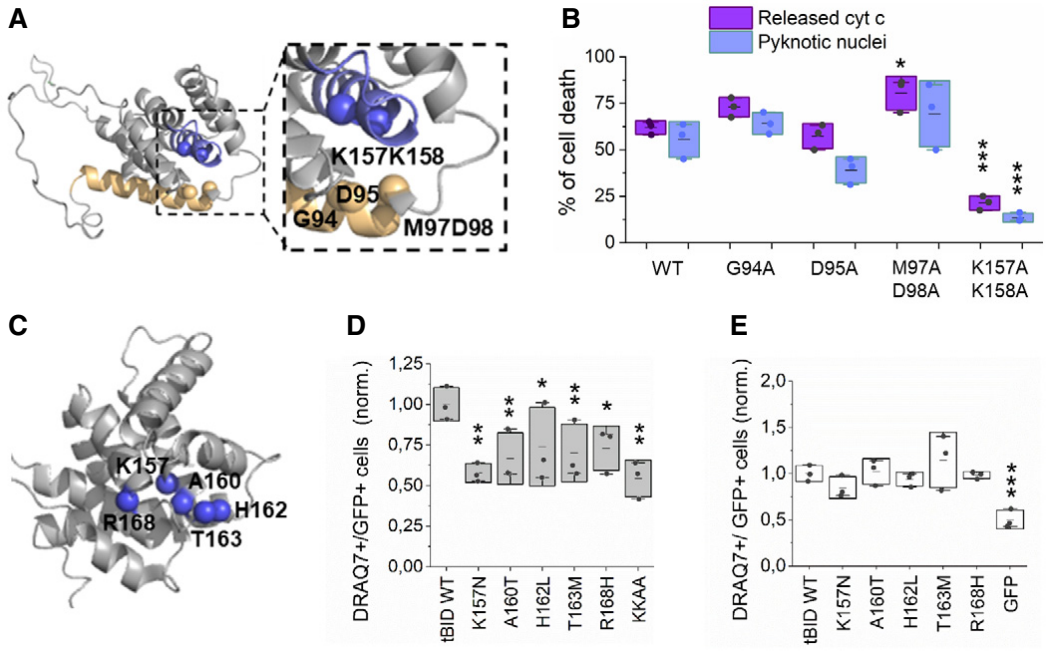
tBID induces apoptosis independently of its BH3 domain, but requires an intact α‐helix 6 Structure of BID (PDB:2BID) depicting residues G94, D95 and M97‐D98 localized at the BH3 domain in light orange, and K157/K158 at the α‐helix 6 in blue.Cell death induced by tBID‐GFP and its variants on HCT AKO, measured as percentage of cells showing released cyt c or pyknotic nuclei. Data correspond to three independent experiments, with *n* > 30 cells per condition per experiment. ***P < 0.001 and *P < 0.05 with respect to tBID‐GFP WT condition.Structure of BID (PDB:2BID) depicting cancer‐related residues localized at α‐helix 6 in blue.Effect of tBID wt and α6‐mutants overexpression on HCT AKO cells, measured as DRAQ7^+^/GFP^+^ cells and normalized to tBID wt condition. *n* = 3 independent experiments, **P < 0.025 and *P < 0.05 with respect to tBID‐GFP wt condition.Effect of tBID wt and α6‐mutants overexpression on MEF BID KO cells, measured as DRAQ7^+^/GFP^+^ cells and normalized to tBID wt condition. Effect of GFP overexpression on BID KO cells relative to tBID wt was assessed by pyknotic nuclei formation quantification. *n* = 3 independent experiments ***P < 0.001 with respect to tBID‐GFP wt condition. Data information: In B, D and E, dots correspond to independent experiments, boxes represent 96% confidence interval, the average is represented by the line inside the box and whiskers correspond to SD and the statistical significance was assessed by one‐way analysis of variance (ANOVA).

**Figure EV4:**
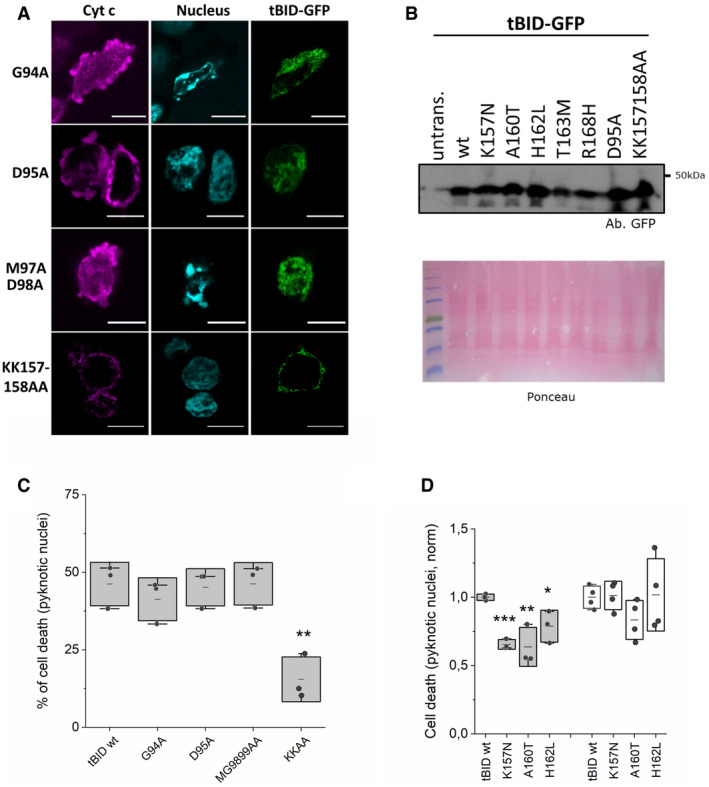
tBID undergoes apoptosis independently of its BH3 domain and dependent on its α‐helix 6 Representative confocal immunofluorescence images of tBID‐GFP G94A, D95A, M97A/D98A and K157A/K158A mutants expressed in HCT 116 AKO. Cytochrome c (magenta), nuclei (cyan) and tBID‐GFP localization (green). Scale bar is 10 µm.Western blot of the expression levels of tBID‐GFP wt and its mutants when overexpressed in HCT AKO cells.Quantification of pyknotic nuclei formation in HCT AKO cells expressing tBID‐IRES‐GFP wt, G94A, D95A, MG9899AA and KK157158AA mutants. Each dot corresponds to a technical replicate from *n* = 3 independent experiments with > 30 cells per condition per experiment. **P < 0.025 with respect to wt condition.Quantification of pyknotic nuclei formation in cells expressing tBID‐IRES‐GFP wt, K157N, A 160 T and H162L mutants (HCT AKO cells (grey) and MEF BID KO (white). Each dot corresponds to a technical replicate with *n* > 30 cells per condition per experiment from 3–4 independent experiments. *P < 0.05, **P < 0.025 and ***P < 0.001 with respect to wt condition. Data information: In C and D, boxes represent 96% confidence interval, the average is represented by the line inside the box and whiskers correspond to SD. The statistical significance was assessed by unpaired Student’s *t*‐test. Source data are available online for this figure.

We next set out to dissect the molecular mechanism how tBID induces apoptotic cell death. To this aim, we explored the putative regions responsible for the ability of tBID to trigger MOMP. We first evaluated the role of the BH3 domain, involved in protein/protein interactions critical for BAX/BAK activation and for blocking of the anti‐apoptotic BCL‐2‐family members (Kim *et al*, 2006; Fletcher *et al*, 2008; Czabotar *et al*, 2013). As shown in Figs 3A and B, and EV4A–D, versions of tBID‐GFP or tBID‐IRES‐GFP harbouring the mutations G94A, D95A or M97A/D98A in the BH3 domain maintained a similar killing activity in HCT AKO cells compared to the wild‐type protein. These results suggest that the BH3 domain, usually involved in BH3‐into‐groove interactions among BCL‐2 family members, including BAX and BAK homo‐oligomerization, is not required for MOMP induced by tBID.

Then, we examined the role of the central hydrophobic α‐helix 6 of BID, which is structurally equivalent to the pore‐forming helix α5 in the piercing domain of BAX and BAK (Fig 3A) (Chou *et al*, 1999; McDonnell *et al*, 1999; Suzuki *et al*, 2000; Garcia‐Saez *et al*, 2005; Petit *et al*, 2009; Sanchez‐Puelles *et al*, 2019). Remarkably, changing two consecutive lysine residues at positions 157 and 158 to alanine (K157A/K158A) dramatically reduced the ability of tBID‐GFP to kill in HCT AKO cells. These results suggest that the α‐helix 6 of tBID is required for tBID killing activity, likely by directly participating in the permeabilization of the outer mitochondrial membrane (Figs 3A and B, and EV4C).

In support for a functional role of the α‐helix 6 of tBID, analysis of the TCGA data base revealed that several mutations in this helix were found in cancer patient samples, including: K157N, A160T, H162L, T163 M and R168H (Fig 3C). The fact that these BID mutants still have an intact BH3 domain to interact with effector and pro‐survival BCL‐2 proteins raised the question whether they could be functionally linked to the ability of tBID to induce apoptosis independently of BAX and BAK. According to this hypothesis, the tBID variants should be able to activate BAX and BAK in a similar manner to wild‐type tBID, while exhibiting reduced killing activity in the absence of BAX and BAK. Indeed, despite being expressed at similar levels as tBID wt (Fig EV4B), these mutations partially impaired tBID killing activity in HCT AKO cells, with the K157N mutant being most affected (Fig 3D). In contrast, the overexpression of the respective tBID mutants in *Bid^‐/‐^* MEF led to a similar degree of cell death as the overexpression of wild‐type tBID in the same cell line (Fig 3E). These results were also confirmed with a selection of these mutants using the IRES‐GFP construct (Fig EV4D). Together, these results support the notion that these tBID mutations found in cancer patients do not affect its ability to activate BAX and BAK, but to induce MOMP in their absence.

### tBID perturbs mitochondrial structure without recognizable self‐assembly into defined nanostructures

**Figure 4:**
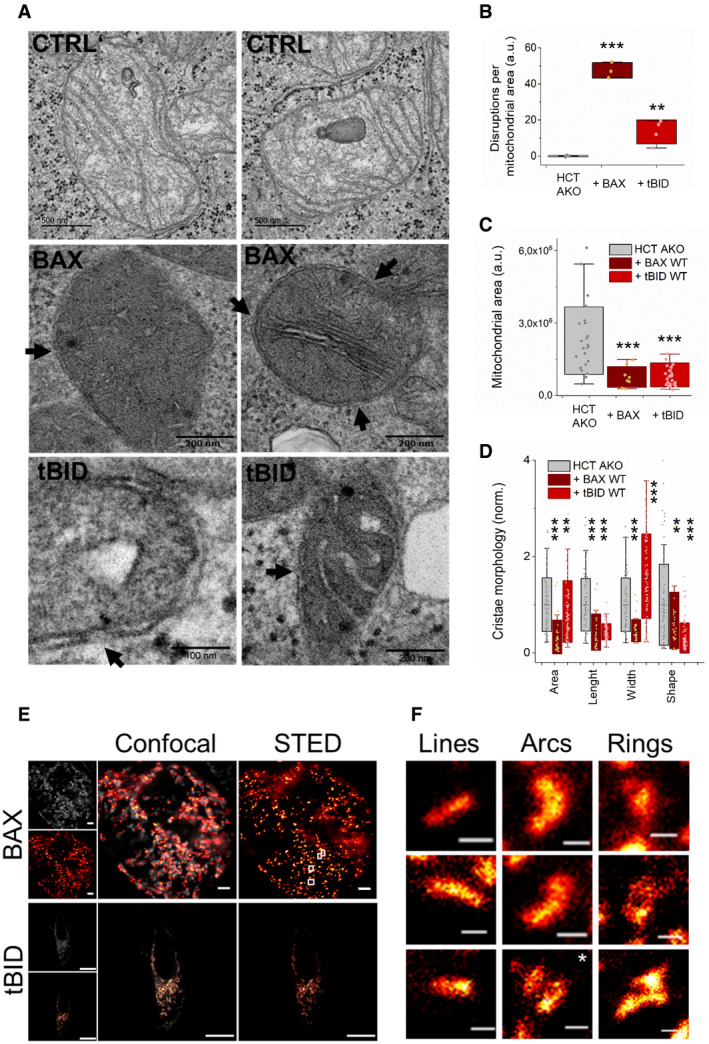
tBID‐induced mitochondrial fragmentation, MOMP and cristae remodelling does not require its oligomerization into supramolecular assemblies ARepresentative CLEM images of mitochondria in HCT AKO cells (control) and HCT AKO cells expressing GFP‐BAX or tBID‐GFP. Black arrows indicate MOM disruptions.B–DEffect of GFP‐BAX and tBID‐GFP on HCT AKO mitochondrial structure. (B) Number of disruptions at the MOM per mitochondrial area, each dot represents an individual cell. (C) Mean area per mitochondrion at the condition tested, each dot represents an individual mitochondrion. (D) Analysis of cristae morphology, each dot represents an individual crista. Cristae shape calculated as length/width. Data represent *n* > 50 mitochondria from 3–5 independent cells. In (B–D), boxes represent 96% confidence interval, the average is represented by the line inside the box and whiskers correspond to SD. The statistical significance was assessed by unpaired Student’s *t*‐test. ***P < 0.001 and **P < 0.025 with respect to HCT AKO or untransfected condition.ERepresentative confocal and STED images of GFP‐BAX and tBID‐GFP expressed in HCT AKO. Scale bar is 1 µm for BAX images, 5 µm for tBID images.FZoomed‐in structures of GFP‐BAX supramolecular assemblies with the shape of lines (*double lines), arcs and rings in HCT AKO cells. Scale bar is 100 nm.

Since mitochondrial fragmentation and reorganization are a hallmark of BAX/BAK‐induced apoptosis, we decided to perform a comparative analysis of the mitochondrial phenotype during BAX and tBID‐induced apoptosis in HCT AKO cells (Fig 4A and Appendix Fig S3A and B). Both proteins induced disruptions of the MOM, which were more abundant in the case of BAX compared to tBID (Fig 4A and B and Appendix Fig S3A and B). Both BAX and tBID promoted mitochondrial fragmentation, quantified as the reduction of the average area of individual mitochondria (Fig 4C). Cristae remodelling was also observed in apoptosis induced by both proteins, albeit with a different phenotype. BAX‐mediated MOMP led to a reduction in cristae size, which otherwise maintained their shape (length/width ratio). In contrast, tBID overexpression maintained the overall area of the cristae, but altered their shape by enlarging the width in comparison with the length, resulting in more roundish, seemingly blown‐up cristae (Fig 4D and Appendix Fig S3A and B).

In light of these results, we investigated whether tBID, as observed for BAX, would also self‐assemble into distinct supramolecular structures in correlation with the mitochondrial alterations linked to MOMP. BAX has been shown to organize into large assemblies with the shapes of lines, arcs and rings, linked to apoptotic pores and co‐localizing with the sites where mtDNA release occurs (Grosse *et al*, 2016; Salvador‐Gallego *et al*, 2016; McArthur *et al*, 2018; Riley *et al*, 2018). However, in contrast to BAX, tBID appeared largely homogeneously distributed in the mitochondria of HCT AKO cells in confocal microscopy images (Fig 4E). Since the distinct structures of BAX could only be detected with super‐resolution techniques, we used STED (Stimulated emission depletion) microscopy to compare the nanoscale organization of tBID‐GFP and GFP‐BAX in apoptotic HCT AKO cells. In agreement with previous results, GFP‐BAX organized into defined supramolecular assemblies, such as lines, arcs and rings also in this cell line (Fig 4E and F and Appendix Fig S3C), indicating that other BCL‐2 proteins are not required for this phenotype. However, we could not identify any distinct structures of tBID‐GFP under the same conditions (Fig 4E), perhaps due to its reduced homo‐oligomerization tendency (Bleicken *et al*, 2012). These findings suggest that accumulation of tBID into discrete foci and assembly into lines, arc or rings is not needed for tBID‐induced MOMP or that they would be significantly smaller and below the spatial resolution of our STED microscope, which sets it apart from the effector protein BAX.

### SMAC release from mitochondria during *Shigella* infection is dependent on tBID and does not require BAX and BAK activation

The X‐linked inhibitor of apoptosis protein (XIAP) is a potent caspase inhibitor that also regulates innate immunity via NF‐κB and mitogen‐activated protein kinase (MAPK) cascade activation (Silke & Meier, 2013). XIAP can be antagonized by cytosolic SMAC, which is normally released from mitochondria during apoptosis. The enteroinvasive bacterium *Shigella flexneri* evades XIAP‐mediated immune responses by inducing the selective release of SMAC from mitochondria, which happens in a BID‐dependent manner. For this, *Shigella* induces the calpain‐dependent cleavage of BID into tBID that releases SMAC, but not cyt c, in absence of apoptosis (Andree *et al*, 2014).

**Figure 5:**
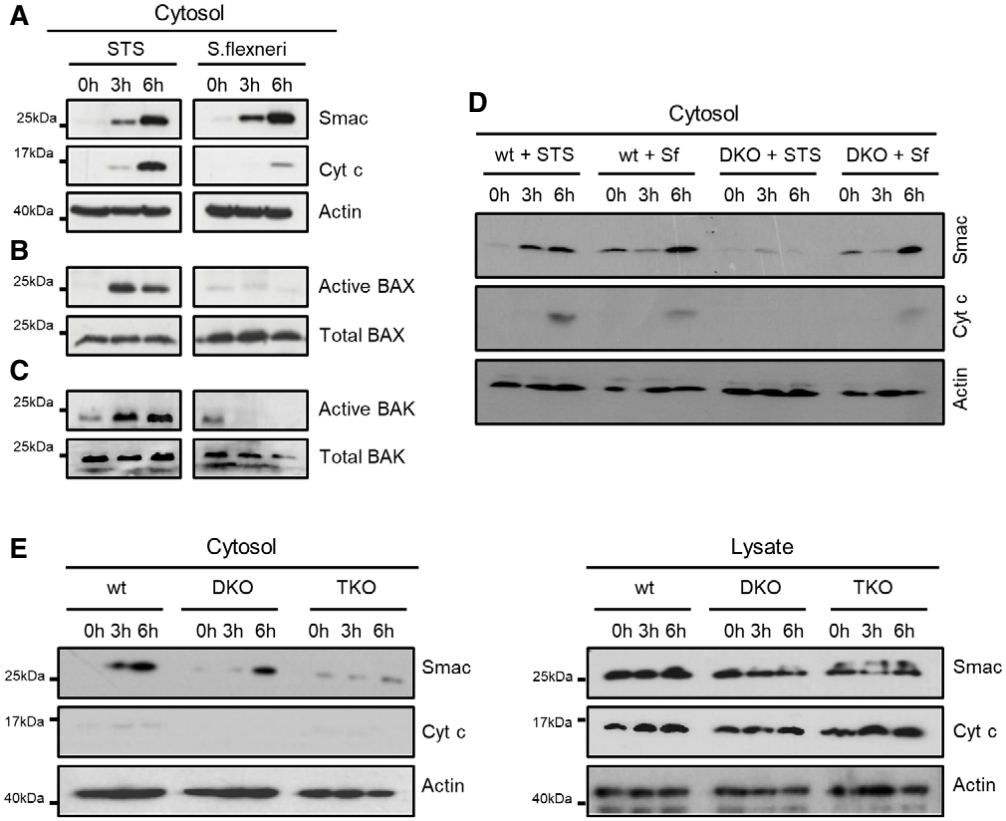
BID‐dependent release of SMAC during *Shigella* infection does not require BAX/BAK activation Arepresentative western blot of cytosolic cyt c and SMAC in HeLa cells.B, CWestern blot analysis of active BAX (B) and BAK (C), in HeLa cells treated with STS or infected with *Shigella flexneri* (MOI 30) at different time points after infection.DRepresentative western blot of cytosolic cyt c and SMAC in HeLa wt and HeLa BAX/BAK DKO cells upon staurosporine (STS) treatment and *Shigella flexneri* infection (Sf).ERepresentative western blot analysis of cytosolic and total (lysate) cyt c and SMAC in HCT wt, HCT DKO and HCT TKO cells infected with *Shigella flexneri* at the indicated time points after treatment/infection. Source data are available online for this figure.

We hypothesized that selective MOMP induced by tBID might play a role in SMAC release upon *Shigella* infection, independently of BAX and BAK. To test this, we infected HeLa cells with *Shigella flexneri* M90T and confirmed the induction of SMAC release and, to a lesser extent cyt c release, as previously described (Fig 5A; Andree *et al*, 2014). Remarkably, under these conditions, we could not detect BAX or BAK activation, as determined by immunoprecipitation against active BAX and BAK (Fig 5B and C). As a positive control, we observed both SMAC and cyt c release, as well as efficient BAX and BAK activation in apoptotic HeLa cells stimulated with STS (Fig 5A–C). In agreement with this, *Shigella* infection of HeLa cells lacking BAK and BAX (HeLa DKO) elicited SMAC release at equivalent levels of wt cells (Fig 5D).

To further dissect the functional role of BID and BAX/BAK in this model, we infected HCT 116 WT, DKO and TKO cells with *Shigella flexneri* M90T. Importantly, we detected similar levels of SMAC release in both wild‐type and DKO cells, but not in the TKO cells lacking BID (Fig 5E). These results indicate that upon *Shigella* infection, tBID is required for mitochondrial permeabilization and selective SMAC release, which occurs without activation of BAX or BAK. Our findings point out a pathophysiological role of tBID‐mediated and BAX/BAK‐independent MOMP in cellular inflammatory signalling.

### tBID kills venetoclax‐resistant leukaemia cells lacking active BAX and BAK

**Figure 6:**
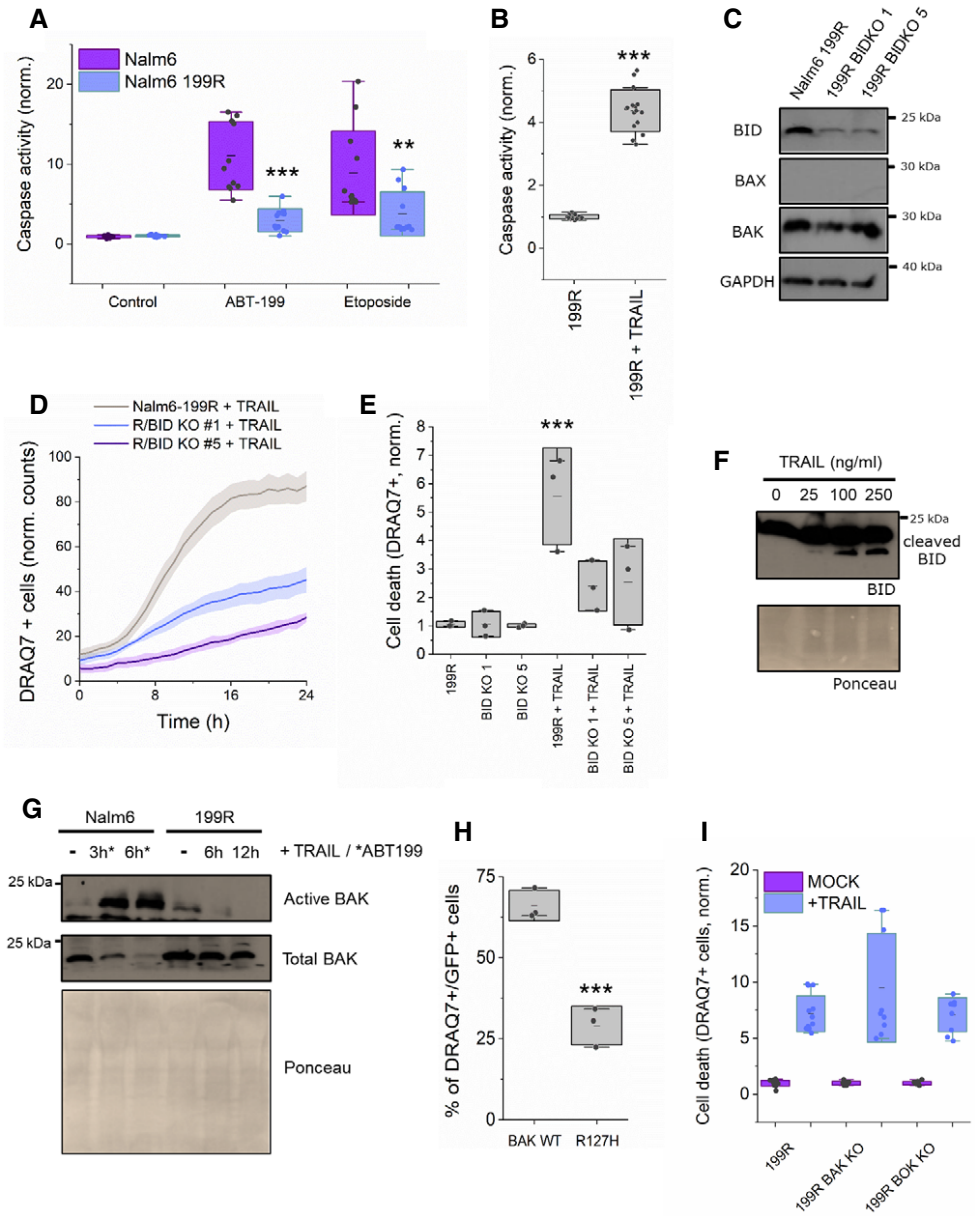
TRAIL‐induced BID cleavage mediates apoptosis in Nalm6 venetoclax‐resistant cells without activation of BAX and BAK ACaspase‐3/7 activity induced by ABT199 and etoposide on Nalm6 (venetoclax‐sensitive) and Nalm6 199R (venetoclax‐resistant) cells, normalized to untreated cells. Unpaired Student’s *t*‐test ***P < 0.001 and **P < 0.025 (Nalm6 with respect to Nalm6 199R at different conditions). Dots correspond to technical replicates of *n* = 3 independent experiments.BCaspase‐3/7 activity in Nalm6 199R cells with or without 250 ng/ml TRAIL stimulation, normalized to untreated cells. Unpaired Student’s *t*‐test ***P < 0.001. Dots correspond to technical replicates of *n* = 3 independent experiments.CRepresentative western blot analysis of BID, BAX and BAK expression levels in Nalm6, Nalm6 199R, Nalm6 199R BID KO #clone1 and Nalm6 199R BID KO #clone5.DRepresentative kinetics of cell death, represented as DRAQ7^+^ cells in Nalm6 199R cells (grey), Nalm6 199R BID KO #1 cells (blue) and Nalm6 199R BID KO #5 cells (purple) upon 250 ng/ml TRAIL treatment. Plots show the average fluorescence intensity (dark lines) and its standard deviation (shaded areas) from five technical replicates. Fluorescence intensity was obtained by analysing the total fluorescence per well using incucyte system every 60 min.EEffect of 24‐h incubation with 250 ng/ml TRAIL on Nalm6 199R, Nalm6 199R BID KO #1 and Nalm6 199R BID KO #5, measured as DRAQ7^+^ cells and normalized to untreated Nalm6 199R cells. Each dot corresponds to the average normalized value of 3–5 technical replicates in *n* = 3 independent experiments, ***P < 0.001 with respect to untreated conditions.FRepresentative western blot of TRAIL‐induced BID cleavage in Nalm6 199R cells at increasing concentrations of TRAIL (0, 25, 100 and 250 ng/ml) for 4 h.GRepresentative western blot analysis of BAK activation in Nalm6 and Nalm6 199R cells at different time points after treatment.HCell death induced by GFP‐BAK and GFP‐BAK R127H on HCT AKO, measured as DRAQ7^+^/GFP^+^ cells. Unpaired Student’s *t*‐test. ***P < 0.001 with respect to GFP BAK wt.IEffect of 24‐h incubation with 250 ng/ml TRAIL on Nalm6 199R, Nalm6 199R BAK KO and Nalm6 199R BOK KO, measured as DRAQ7^+^ cells with respect to total cell surface and normalized to untreated conditions. Each dot corresponds to the average normalized value of 3–4 technical replicates in *n* = 3 independent experiments. Data information: Unless otherwise stated in A, B, E and H, dots correspond to independent experiments, boxes represent 96% confidence interval, the average is represented by the line inside the box and whiskers correspond to S.D and the statistical significance was assessed by one‐way analysis of variance (ANOVA). Source data are available online for this figure.

**Figure EV5:**
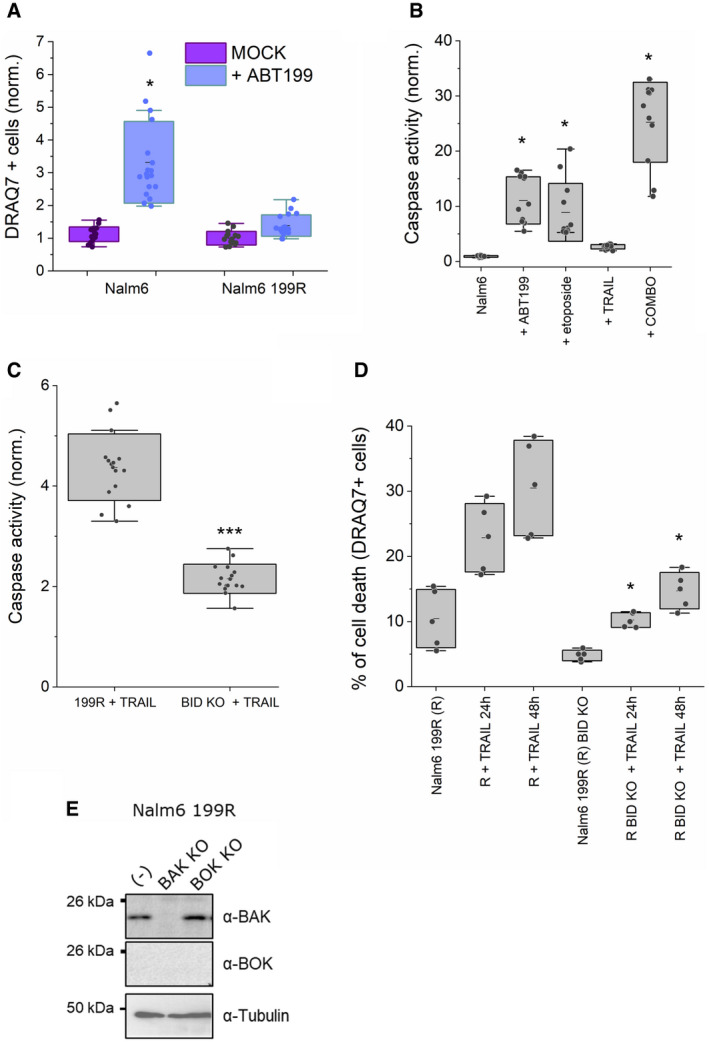
TRAIL‐induced BID cleavage mediates cell death in Nalm6 venetoclax‐resistant cells Effect of ABT199 on Nalm6 and Nalm6 199R, measured as DRAQ7^+^ cells. Each dot represents one technical replicate from three independent experiments. *P < 0.05 (treated with respect to untreated cells).Caspase 3/7 activity in Nalm6 cells when stimulated with indicated drugs and normalized to untreated cells *P < 0.05.Caspase 3/7 activity in Nalm6 199R and Nalm6 199R BID KO cells stimulated with TRAIL (250 ng/ml). ***P < 0.001 (with respect to 199R + TRAIL condition).Effect of TRAIL on Nalm6 199R and Nalm6 199R BID KO, measured as DRAQ7^+^ cells at different times with > 10,000 cells per condition per experiment. *P < 0.05 (with respect to untreated Nalm6 199R condition) *n* = 5 technical replicates from two independent experiments.Western blot showing the endogenous levels of BAK and BOK in Nalm 6 199R, Nalm 6 199R BAK KO and Nalm 6 199R BOK KO cells. Of note, endogenous BOK levels could not be detected by WB in the parental 199R cells. Data information: Unless otherwise stated in A–D, dots correspond to independent experiments, boxes represent 96% confidence interval, the average is represented by the line inside the box and whiskers correspond to SD. The statistical significance was assessed by one‐way analysis of variance (ANOVA). Source data are available online for this figure.

Venetoclax (or ABT‐199) is a clinically approved BH3‐mimetic molecule that efficiently neutralizes BCL‐2, thereby leading to BAX/BAK activation and MOMP to induce apoptosis (Fischer *et al*, 2019). By continued exposure to venetoclax *in vitro*, we generated a resistant variant of Nalm6 pre‐B ALL cells, named Nalm6 199R (Figs 6A and EV5A and B). Nalm6 199R cells are also resistant to other apoptotic stimuli like etoposide, while remaining sensitive to TRAIL (Fig 6A and B). They have lost BAX and express an inactive version of BAK due to a mutation in its BCL‐2 binding groove (BAK R127H) (Fig 6C and H; Dewson *et al*, 2008; Dai *et al*, 2011; Pang *et al*, 2012; Brouwer *et al*, 2014).

We then used these cells to explore the potential use of tBID to directly induce MOMP and kill venetoclax‐resistant cells. To test whether the remaining TRAIL sensitivity was dependent on tBID, we generated Nalm6 199R cells depleted of BID (Fig 6C) and treated them with TRAIL. Genetic ablation of BID significantly reduced cell death induced by TRAIL (Figs 6D and E, and Fig EV5C and D), in support of our hypothesis. Importantly, under these conditions, we detected activation of tBID but not of BAK (Fig 6F and G). The impaired apoptotic activity of BAK R127H in HCT AKO cells serves as an additional control that TRAIL‐induced apoptosis in Nalm6 199R cells was independent of BAK (Fig 6H). In addition, deletion of BAK or BOK in Nalm6 R199 cells did not affect TRAIL‐induced cell death (Figs 6I and EV5E). These results pinpoint the therapeutic potential of tBID activation to kill leukaemia cells defective in BAX and BAK activation. Our analysis above (Fig 3) also predicts potential mechanisms of drug resistance, already found in cancer patients, i.e., selecting for mutations in alpha 6 of BID.

## Discussion

Here, we report on a hitherto unknown function of tBID as a direct effector of MOMP that is capable of releasing apoptotic factors into the cytosol, activating the caspase cascade and triggering apoptotic cell death. We show that this mitochondrial permeabilization mediated by tBID is independent of BAX and BAK and physiologically relevant for the release of SMAC to dampen XIAP‐mediated immune responses in the context of *Shigella* infection. Moreover, activating endogenous BID can be clinically relevant for therapy to kill venetoclax‐resistant leukaemia cells lacking active BAX and BAK.

Our findings call for a reclassification of the BCL‐2 family. tBID has so far been classified as a “direct activator” BH3‐only protein due to its ability to directly activate BAX and BAK, as well as to inhibit the pro‐survival BCL‐2 family members (Landeta *et al*, 2014; Bleicken *et al*, 2017; Kale *et al*, 2018; Flores‐Romero *et al*, 2019; Singh *et al*, 2019). tBID exerts these functions via physical interaction of its BH3 domain with the hydrophobic groove present in the globular fold of both effector and anti‐apoptotic BCL‐2 proteins (Muchmore *et al*, 1996; Chou *et al*, 1999; McDonnell *et al*, 1999; Suzuki *et al*, 2000; Bleicken *et al*, 2017). We demonstrate here that, in addition, tBID can directly permeabilize mitochondria and release cyt c and SMAC to induce apoptosis, a function that was so far solely attributed to the effector proteins BAX and BAK, and recently also to the closely related BCL‐2 protein BOK (Llambi *et al*, 2016).

Interestingly, this newly discovered activity of tBID does not rely on its BH3 domain, suggesting that it does not require canonical BH3‐into‐groove interactions with itself nor with other BCL‐2 family members. This suggests that oligomerization may not be fundamental for tBID‐induced MOMP. In agreement with this, we noted reduced self‐interactions between tBID molecules in chemically controlled minimal systems in our previous studies (Bleicken *et al*, 2012). We also found that tBID in cells undergoing MOMP in the absence of BAX, BAK and other BCL‐2 proteins is not accompanied by accumulation of the protein into discrete foci, neither by the formation of distinct supramolecular structures, as we and others reported for BAX (Grosse *et al*, 2016; Salvador‐Gallego *et al*, 2016). This raises the intriguing question whether high‐order oligomerization is indeed necessary for BAX and BAK‐induced MOMP, or whether this feature is serving a different purpose. Although so far it was not possible to disentangle BAX and BAK oligomerization from their pore‐forming activity, previous studies suggested that their potency to induce cytochrome c release did not directly correlate with the extent of oligomerization (Xu *et al*, 2013; Kuwana *et al*, 2020). Nevertheless, oligomerization of BAX and BAK is likely to play a role in the efficiency of pore formation for MOMP and/or in the enlargement and stabilization of the apoptotic pore (Zhang *et al*, 2016).

Despite its dispensability for MOMP, the BH3 domain of tBID could serve a regulatory function. In support of this, co‐expression of tBID with anti‐apoptotic protein BCLXL significantly reduced the ability of tBID to induce cell death. In addition, the overexpression of tBID in HCT DKO cells did not induce cell death unless the anti‐apoptotic proteins were inhibited with ABT‐737 and S63845 combination treatment.

The ability of tBID to mediate MOMP instead required an intact helix 6, which is homologous to the helix 5 of BAX and BAK (Chou *et al*, 1999; McDonnell *et al*, 1999; Suzuki *et al*, 2000; Billen *et al*, 2008), and which we and others previously showed is able to form pores in model membrane systems (Zhai *et al*, 2001; Garcia‐Saez *et al*, 2004, 2005; Ma *et al*, 2019). Indeed, the sequences of helix 6 of tBID and the homologous helices 5 of BAX and BAK share several features, such as their amphipathic character, the short length of their hydrophobic stretch, the presence of flanking lysine residues and the affinity for negatively charged phospholipids. Remarkably, these properties are also typically observed in membrane pore‐forming antimicrobial peptides (Ros & Garcia‐Saez, 2015; Cosentino *et al*, 2016; Flores‐Romero *et al*, 2020). This argues in favour of helices 5 (and likely 6) of BAX and BAK, as well as helix 6 of tBID, constituting minimal domains responsible for MOMP, as proposed before (Basanez *et al*, 1999; Garcia‐Saez *et al*, 2004; Garcia‐Saez *et al*, 2005; Fuertes *et al*, 2010; Flores‐Romero *et al*, 2017; Flores‐Romero *et al*, 2020). In support of a functional role of helix 6 of tBID, we also found that the amino acid substitutions K157N, A160T, H162L, T163 M and R168H, detected in cancer patients, drastically reduced the ability of tBID to induce MOMP.

Based on this, we propose a general concept, in which it is the exposure of helices 5 of BAX and BAK, or helix 6 of tBID, that unleashes their ability to induce MOMP. In BAX and BAK, this would be triggered by their conformational changes upon activation at the MOM, while in the case of tBID, this is enabled via cleavage by caspase‐8 and translocation to the MOM. Accordingly, we speculate that high‐order oligomerization would not be required to cytochrome c release, but mainly contribute to later stages of MOMP by increasing the efficiency of pore formation, as well as the stability and growth rate of the apoptotic pore. The higher tendency of BAX and BAK to oligomerize would, therefore, underlie their higher potency to induce MOM disruptions, as detected by EM, as well as cell death, compared to tBID.

When comparing the mitochondrial alterations accompanying MOMP induced by tBID versus BAX, we found that both proteins were able to promote mitochondrial fragmentation to a similar extent. However, their effect on mitochondrial cristae was different, suggesting that mitochondrial fragmentation and cristae remodelling can be uncoupled. tBID, but not BAX, was capable of rearranging the cristae shape from linear to roundish while BAX decreased the overall cristae area while maintaining the shape. This is in coherence with previous results in apoptotic MEF cells, reporting that tBID presents a potent mitochondrial remodelling activity (Scorrano *et al*, 2002). Such activity involved individual cristae becoming fused and opening of the junctions between the cristae and the intermembrane space, with the subsequent mobilization of the cyt c stores. Importantly, we show here that this mitochondrial remodelling activity of tBID does not depend on BAX or BAK. In this scenario, one could envision that during apoptosis two parallel mitochondrial processes may be induced by BCL‐2‐effectors. The first would require cristae remodelling to free cyt c from the cristae, where tBID would be more efficient than BAX, and likely BAK. The second one would involve disruptions of the MOM to release the apoptotic factors to the cytosol, where BAX and BAK would be more potent than tBID.

BID structurally adopts the BCL‐2 fold and is, therefore, more similar to the BCL‐2 multi‐domain proteins than to the unstructured BH3‐only family members. It also contains an amphipathic central helix with pore‐forming properties, yet it seems to have a reduced capacity to self‐assemble into high‐order oligomers. Considering this and previous knowledge, we propose a new framework for the classification of BCL‐2 proteins that is based on the BCL‐2 fold, which harbours two main features. First, the BCL‐2 fold in the water‐soluble conformation shields the two central hydrophobic helices whose amphipathic character determines the pore‐forming properties of the proteins, and thereby their ability to induce MOMP. In this regard, the central helices of BAX, BAK, BID and BOK present a sequence typical of pore‐forming peptides. In contrast, the central helix of BCLXL lacks the flanking positive residues and does not seem able to form membrane pores (Muchmore *et al*, 1996; Garcia‐Saez *et al*, 2004). Second, the BCL‐2 fold provides the backbone for the hydrophobic groove that allows oligomerization and regulatory interactions with proteins containing BH3 domains. These BH3‐into‐groove interactions will be pro‐apoptotic if they favour the conformational change and exposure of the pore‐forming helix in the BH3‐receiving protein, or anti‐apoptotic, if the binding of the BH3 domain does not trigger a conformational change or if the helix exposed cannot form membrane pores. Besides incorporating tBID and BOK as rightful MOMP effectors, this model would also explain the capacity of many pro‐survival BCL‐2 proteins to promote apoptosis under certain conditions (Cheng *et al*, 1997; Basanez *et al*, 2001; Hellmuth & Stemmann, 2020). This phenotypic switch could be driven by disruption of their groove as a sink for BH3 domains, and/or by the potential pro‐apoptotic function of their BH3 domains or of their central helices.

Finally, we present two scenarios in which the ability of endogenous tBID to induce mitochondrial membrane permeabilization independently of BAX and BAK is of pathophysiological relevance. On the one hand, the enteroinvasive bacterium *Shigella flexneri* induces calpain‐dependent BID cleavage and SMAC release to evade the XIAP‐dependent NF‐kB activation and immune responses (Andree *et al*, 2014). We show that this mitochondrial permeabilization that releases SMAC occurs without activating BAX or BAK and depends on the presence and proteolytic activation of BID. In line with this, a recent study showed that caspase‐1, in the absence of Gasdermin D, cleaves tBID and induces SMAC release in the context of *Salmonella* infection (Heilig *et al*, 2020). This suggests that SMAC release mediated by tBID‐induced MOM permeabilization plays a more general role in the defence against different bacterial infection (Campbell *et al*, 2020; Heilig *et al*, 2020; Morrish *et al*, 2020).

Additionally, we demonstrate the therapeutic potential of tBID‐mediated MOMP in venetoclax‐resistant Nalm6 leukaemia cells lacking active BAX and BAK, in which TRAIL treatment efficiently induces BID‐dependent apoptosis. In this regard, our results open new avenues for the use of BID activation as a strategy to kill cancer cells that have become insensitive to BAX and BAK. However, our findings made in the TCGA data base, identifying mutations in helix a 6, already point towards possible resistance mechanisms that may arise in such cancers, once treated with TRAIL. Whether low‐dose treatment of Nalm6 R199 cells with TRAIL selects for such mutations in BID will be subject of future studies.

In summary, when and how a cell undergoes MOMP is the result of the interplay of all BCL‐2 proteins simultaneously present in that cell, which integrates of all the pro‐ and anti‐apoptotic functions that these proteins can perform. Our findings show that, in the normal context of the cell, tBID is capable of performing three distinct pro‐apoptotic functions, similar to BAX and BAK. First, it can block the prosurvival BCL‐2 members via complex formation with them. Second, tBID can activate BAX and BAK through direct interaction with them. Interestingly, BAX and BAK can also auto‐activate themselves with a similar mechanism how tBID activates them (Pagliari *et al*, 2005; O'Neill *et al*, 2016; Iyer *et al*, 2020). And third, tBID can also induce MOMP directly and initiate apoptosis. Although the MOMP‐effector function of tBID is less efficient than that of BAX and BAK, and thus it will likely not dominate under conditions when all effectors are activated, this newly recognized activity of tBID can be decisive in some (patho)‐physiological scenarios, like in the context of Shigella infection, or in tumours that have become resistant to BAX and BAK, as we report here. Our discovery opens new opportunities for the design of novel strategies to fight against bacterial infection and cancer.

## Materials and Methods

### Chemicals and reagents

ABT‐737 (APEX Bio), S63845 (ActiveBiochem), TRAIL (Abcam), DRAQ7 (Biolegend), Propidium iodide (PI) (Thermo Fisher Scientific), Digitonin (Sigma Aldrich), CHAPS (Sigma Aldrich) and MitoTracker 561/647 (Thermo Fisher Scientific). ZVAD, NSA, Ferrostatin‐1 from Invitrogen and Cs‐A, MG132, Bortezomib, staurosporine (STS) and rotenone from Sigma Aldrich.

### Cell culture

HeLa wt, HeLa BAX/BAK KO and BAX/BAK DKO U2OS cells were cultured in low‐glucose Dulbecco’s modified Eagle’s medium (DMEM) (Sigma‐Aldrich), MEF BID KO cells with high‐glucose DMEM and HCT WT, BAX/BAK DKO, BAX/BAK/BID TKO, all BCL‐2 KO and all BCL‐2 KO BOK KO cells with McCoy’s 5A medium (Gibco); all of them supplemented with 10% foetal bovine serum (FBS), 1% penicillin–streptomycin (P/S) (Thermo Fisher Scientific) at 37°C in a humidified incubator containing 5% CO_2_. Nalm6, Nalm6 199R, Nalm6 199R BID, BAK and BOK single KO cells were cultured in with RPMI (Gibco) media with 20% foetal bovine serum (FBS), 1% penicillin–streptomycin (P/S) (Thermo Fisher Scientific) and incubated under the same conditions. HCT116 WT, BAX/BAK DKO HCT116 were a generous gift from Dr Schulze‐Osthoff from the University of Tübingen. MEF BID KO and HCT all BCL‐2 KO were generated previously (Andree *et al*, 2014; O'Neill *et al*, 2016). BAX/BAK DKO U2OS ATCC^®^ HTB‐96. BAX/BAK/BID TKO HCT116, BOK KO all BCL‐2 KO HCT116, Nalm6 199R BID, BAK and BOK single KO cells were generated by CRISPR/Cas9.

### CRISPR/Cas9 knockout cell line generation

BOK, caspase‐9 and BID CRISPR/Cas9 knockout were generated in HCT116 all Bcl‐2 KO (O'Neill *et al*, 2016) and HCT116 BAX/BAK DKO generating HCT AKO BOK KO and HCT BAX/BAK/BID TKO respectively. For CRISPR transfection, 1–2 × 10^5^ cells were seeded in a six‐well plate 48 h before transfection. 500 ng of CRISPR construct was transfected with 1 µl of Lipofectamine 2000 (Thermo Fisher) according to the manufacturer’s instructions. Twenty‐four hours after transfection cells were transferred to a 15‐cm dish and selected for 7 days with media supplemented with 0.5 µg/ml puromycin. Single colonies were picked and cultured for validation. Success of the knockout was validated using western blotting and genotyping by Sanger’s sequencing of the target region. For genotyping, the genomic DNA was isolated from HCT116 cells using DNeasy blood and tissue kit (Qiagen) and the genomic region of interest was amplified by PCR. The PCR product was purified using QIAquick Gel Extraction Kit (Qiagen) and used for Sanger’s sequencing. Plasmid and constructs: the following guide RNA sequences were used for the generation of BOK and BID CRISPR/Cas9 knockout cell lines; *BOK* gRNA CGCCACGTTGCGGTAGACGCtgg, *BID* gRNA GCTCATCGTAGCCCTCCCACtgg and BAK gRNA CACCGCGAGTGTCTCAAGCGC ATCGgtt. Pairs of oligonucleotides containing the gRNA sequence were cloned into the pSpCas9(BB)‐2A‐Puro V2.0 (px459, Addgene #62988) using the restriction enzyme BbsI (NEB). For the Nalm6 199R BID, BAK and BOK single KO cell line generation pU6‐(BbsI)sgRNA_CAG‐Cas9‐venus‐bpA vector was used as described (Yumlu *et al*, 2017). Here, 5 × 10^5^ cells were seeded in a six‐well and 4 µg of CRISPR construct was transfected with 10 µl of Lipofectamine 2000 (Thermo Fisher) according to manufacturer’s instructions. Twenty‐four hours after transfection cells were sorted for mVenus‐positive cells and cultured for validation. Success of the knockout was validated by western blot analysis.

### Cell death assays

Cyt c release and nuclear morphology were characterized as described previously (Flores‐Romero *et al*, 2019) with small modifications. HCT AKO and DKO cells growing onto a coverslip were transiently transfected with plasmids encoding GFP‐tagged BCL‐2 proteins using lipofectamine 2000 (Life Technologies) and incubated for 18–24 h. Next, cells were incubated with 200 nM MitoTracker at 37°C for 30 min and washed with prewarmed media. Then, cells were fixed with 3.8% paraformaldehyde in PBS, washed twice with PBS and incubated in ice‐cold acetone and washed again with PBS. Samples were blocked by using 3% bovine serum albumin (BSA)/0,1% Triton‐X100 in PBS. Coverslips were subsequently immunoblotted with a primary anti‐cyt. c anti‐body (1:200, BD‐556432) and a secondary fluorescent anti‐mouse 633 antibody (1:400, Life Technologies) and finally, the nuclei were dyed with Hoechst 33342 (Invitrogen). When the effect of non‐tagged tBID inducing cyt c was analysed, we detected ectopically expressed tBID by immunoblotting (anti BID 1:200, 2002S Cell Signaling) and a secondary fluorescent anti‐rabbit 488 antibody (1:400, Life Technologies). When mtDNA release was analysed, BAX/BAK DKO U2OS cells were transfected with 3:1 ratio of tBID‐GFP/GFP‐BAX and TFAM‐RFP (Riley *et al*, 2018) for 24 h in the presence of 10 µM ZVAD and fixed as described above. Next, cells were immunostained with primary anti‐TOM (FL‐145, sc‐11415 Santa Cruz) and a secondary fluorescent anti‐rabbit 647 antibody (Life Technologies). Samples were acquired with a Zeiss LSM 710 ConfoCor3 microscope (Carl Zeiss) for cyt c release and nuclear morphology characterization and Leica SP8 microscope both with 63× NA 1.5 oil immersion objective and lightning setup (HyVolution–superresolution, Leica Microsystems GmbH) for mtDNA release experiments. Images were processed by Fiji software. Concentrations used are: ABT‐737/S63845 (5–10 µM for 4–6 h), ZVAD (10 µM), NSA (10 µM), necrostatin‐1 (10 µM), ferrostatin‐1 (2 µM), CsA (1 µM), MG132 (1 µM); bortezomib (1 µM), STS (1 µM) and rotenone (1 µM). ZVAD, NSA, necrostatin‐1 and ferrostatin‐1 were added 6–8 h after transfection.

For caspase‐3/7 measurements, cells were seeded in white bottom 96‐well plates (Greiner Bio‐One) in media without phenol red. Cells were left untreated or treated for 4 h with etoposide (5 μM), STS (1 μM), ABT199 (5 µM), COMBO (ABT737 5 µM/S63845 5 µM), TRAIL (250 ng/ml). Following treatment, cells were subjected to caspase‐3/7 activity measurement with Caspase Glo assay kit (Promega). Briefly, equal amounts of Caspase Glo reagent were added to each well and the plate was incubated for 10 min at room temperature. The total luminescence of each sample was measured in a plate reader (Infinite M200, Tecan). In samples containing GFP‐BAX, tBID‐GFP or its mutants, plasmids were transfected 18–24 h before the caspase activity analysis.

The plasma membrane integrity was tested by flow cytometry and IncuCyte S3 2018B (Sartorius) measuring the ability of cells to exclude PI or DRAQ7. Flow cytometric analyses were conducted using CytoFlex, and data were analysed using the FACS Diva software (Beckman Coulter). After treatment, both attached and non‐attached cell populations were collected. Cells were washed twice with cold PBS, centrifuged (500 g, 5 min, 4°C) and resuspended in PBS containing PI (2 µg/ml). A total of 10,000 cells were counted by flow cytometry, and membrane breakage was determined as a PI‐positive population. *In situ* measured cell death assays were performed using the IncuCyte bioimaging platform (Essen) at 37°C 5% CO_2_; two to four images per well were captured, analysed and averaged. Cell death was measured by the incorporation of DRAQ7 (599/644) (D15106, Thermo). In the experiments using TRAIL, COMBO, ABT199, emricasan and combinations, the cells were pre‐treated with TRAIL 250–1,000 ng/ml for leukaemia cells and HCT116/U2OS respectively for 24, 48 and 72 h and ABT199 5 µM for 24 h and emricasan 300 nM for 48 h. Subsequently, the medium was replaced with medium containing DRAQ7 with or without COMBO (ABT737 5–10 µM S6385 5–10 µM) and cell death was assessed using IncuCyte basic analysis software. In the IncuCyte experiments with leukaemia cells, wells were precoated with 5 µg/ml fibronectin in PBS following manufacturer’s recommendations. For the samples containing GFP‐BAX, GFP‐BAK, tBID‐GFP or its mutants, plasmids were transfected in media containing DRAQ7 and monitored for 24 h and quantified manually.

For cyt c and SMAC release, HCT AKO cells grown in six‐well dishes were harvested in cold PBS by scraping and pelleting at 500 g for 5 min at 4°C. Next, cell pellets were incubated in permeabilization buffer (0,025% digitonin, 20 mM HEPES pH 7.5, 100 mM sucrose, 2.5 mM MgCl_2_, 100 mM KCl, supplemented with freshly prepared protease inhibitors) for 10 min at 4°C. Then, samples were centrifuged at 14,000 g for 10 min at 4°C, collecting the supernatants (containing the cytosolic fractions) and pellets (containing mitochondrial fractions) were resuspended in RIPA buffer (10 mM Tris‐HCl, pH 8.0, 1 mM EDTA, 0.5 mM EGTA, 1% Triton X‐100, 0.1% sodium deoxycholate, 0.1% SDS, 140 mM NaCl supplemented with fresh protease inhibitors) for 30 min at 4°C. In PARP1 cleavage experiments, cells were harvested as described above and then pellets were directly resuspended in urea buffer (6 M) and heated at 95°C for 10 min. Samples were subjected to SDS‐PAGE and immunoblotting analysis using anti‐cyt c (BD‐554233), anti‐SMAC (D5S3R #15108 Cell Signaling) and anti‐PARP (#9542L Cell Signaling). Samples containing overexpressed GFP‐BAX, tBID‐GFP or GFP‐BCLXL, were transfected 18–24 h prior to cell harvesting. In subcellular fractionation experiments, ZVAD (10 µM) was added 6–8 h after transfection.

### Silencing experiments

siRNA transfection was performed in six‐well plates when assessed by WB and in 96‐well plates for the IncuCyte experiments. In the former case, for each well, 20 pmol of BID siRNA (GGAGCCAGCUAGAUAUUAA, Dharmacon) or siRNA control/non‐targeting (D‐001810‐0120, Dharmacon) was premixed with 2‐μl lipofectamine 2000 (Life Technologies) in 200 μl Opti‐MEM (Thermo Fisher). The mixture was incubated for 20 min at room temperature, diluted with 800 μl Opti‐MEM and exchanged for cellular media. After 4–6 h, 1 ml of media supplemented with 20% FBS and 1% P/S was added to each well. If it is not otherwise stated, siRNA transfection was performed during 48 h. The levels of the endogenous protein were checked by immunoblotting. For the IncuCyte experiments, 20 nM (2 pmol) of BID, Caspase‐8 (CASP8 smartpool, Dharmacon) and scramble siRNA per well were used.

### Western blotting

A quantity of 50–250 μg of protein sample were loaded on 8–15% polyacrylamide gels and transferred onto PVDF membrane (Merck Millipore) or Amersham Hybond‐ECL nitrocellulose membranes (GE Healthcare) using the Turboblot (BioRad) or a wet transfer system (BioRad). Blots were incubated overnight at 4°C with primary antibodies, probed with secondary antibodies and developed using ECL (Perkin Elmer). The following primary antibodies were used: anti‐BAX (1:1,000, Cell Signaling #2772), anti‐actin (1:10,000, Cell Signaling #4967), anti‐GAPDH (1:5,000, Santa Cruz sc‐47724), anti‐PARP (1:1,000, Cell Signaling #9542), anti‐BAK (1:1,000, Cell Signaling, #3814), anti‐BID (1:1,000, 2002S Cell Signaling), anti‐BOK (1:1,000, Abcam 186745), anti‐α‐tubulin (1:5,000 #2144 Cell Signaling), anti‐VDAC (1:1,000, D73D12, Cell Signaling), anti‐Smac/Diablo (1:1,000, D5S3R, Cell Signaling), anti‐cytochrome c (1:1,000, BD Pharmingen 556433) and anti‐GFP from mouse IgG1κ (1:1,000, clones 7.1 and 13.1, Roche).

### Immunoprecipitations

Immunoprecipitation of active BAX and BAK was carried out as follows: 200 μg of whole‐cell lysates was brought to a final volume of 500 µl with 1% CHAPS lysis buffer with KCl 150 mM pH 7.4. Samples were incubated for 12 h at 4°C with 2 μg of monoclonal anti‐BAX 6A7 antibody (BD Biosciences, Heidelberg, Germany) or anti‐BAK (BAK ab1, Calbiochem) (Yethon *et al*, 2003; Peyerl *et al*, 2007; Dewson *et al*, 2008). Antigen–antibody complexes were immobilized on Gamma Bind G Sepharose (GE Healthcare, Freiburg, Germany) by rotation for 2 h at 4°C. The complexes were pelleted (1 min, 14,000 *g*) and the supernatant removed. The complexes were then washed three times with lysis buffer and subjected to SDS‐PAGE and western blotting as described above.

### *Shigella* infection

Bacterial infection of the indicated cell lines was performed using the *Shigella flexneri* strain M90T as described previously (Philpott *et al*, 2000). Prior to infection, cells were starved in media without FCS for 1 h followed by incubation with Shigella (MOI = 30) for 15 min at RT and transferred at 37°C for 30 min (time point zero). Plates were then transferred into fresh media containing 10% FCS and 50 µg/ml gentamycin to kill extracellular bacteria. Cellular and cytosolic extracts were prepared as previously described (Kashkar *et al*, 2003).

### STED microscopy

HCT116 AKO cells were grown onto Ibidi µ‐Dish with finder grid‐50 glass bottom, transfected with GFP‐BAX and tBID‐GFP, incubated with 500 nM MitoTracker at 37°C for 30 min and fixed as described above. STED images were acquired using a commercial setup (TCS SP8 gSTED 3X, Leica Microsystems) equipped with a white light laser for excitation and a 592‐nm donut‐shaped laser for depletion. A 100× oil objective with a numerical aperture of 1.4 (HC PL APO CS2 100×/1.40 oil, Leica Microsystems) was used and the signal was acquired with a sensitive HyD detector (Leica Microsystems). Confocal microscopy and gSTED images were processed only for contrast stretching by Fiji. BAX structures were detected by using ASAP program as described previously (Danial & Garcia‐Saez, 2019), followed by a manual classification.

### CLEM

Cells were growing onto Ibidi µ‐Dish with finder grid‐50 glass bottom and fixed for 30 min at room temperature and 30 min at 4°C in 4% formaldehyde with 2.5% sucrose and 100 mM CaCl_2_ in HEPES buffer pH 7.4. Cells were washed three times with 0.1 M cacodylate buffer and fluorescent and brightfield images were taken using the Evos FL Auto 2 (Thermo Fisher) with 20× and 40× objectives. Localization coordinates of cells of interest were noted. Cells were incubated with 1% osmium tetroxide for 30 min at 4°C. After 3 × 5 min wash with 0.1 M cacodylate buffer, samples were dehydrated using an ascending ethanol series (50%, 70%, 90%, 100%) for 7 min each at 4°C. Cells were infiltrated with a mixture of 50% Epon/ethanol for 1 h, 66% Epon/ethanol for 2 h and with pure Epon overnight at 4°C. TAAB capsules filled with Epon were placed upside down onto the glass bottom and cured for 48 h at 60°C. Glass bottom was removed by alternatingly putting the dish into boiling water and liquid nitrogen. The block face was trimmed to the previously noted square using a razor blade and ultrathin sections of 70 nm were cut using an ultramicrotome (Leica Microsystems, UC6) and a diamond knife (Diatome, Biel, Switzerland) and stained with 1.5% uranyl acetate for 15 min at 37°C and lead citrate solution for 4 min. Images were acquired using a JEM‐2100 Plus Transmission Electron Microscope (JEOL) operating at 80 kV equipped with a OneView 4K camera (Gatan). Cells of interest were identified using the fluorescent and brightfield images previously taken. In the samples where STED was performed to resolve for BAX supramolecular structures, fixative was supplemented with 2.5% glutaraldehyde. Image analysis for mitochondrial area, MOM disruptions and cristae morphology was performed manually using Fiji. Briefly, images were analysed by defining the areas of the whole mitochondria measuring their perimeter using the Polygon Selection Tool. Lengths and widths of the cristae were measured using the Line Selection Tool, considering the longest and widest points of each crista.

### TCGA analysis

Somatic mutations of BID previously described in cancer (TCGA cohorts) were retrieved from the COSMIC database v92 (https://cancer.sanger.ac.uk/cosmic, https://pubmed.ncbi.nlm.nih.gov/30371878/). Mutation positions were mapped to transcript ENST00000622694 as a reference. MAF file parsing and analysis of mutations were facilitated by the maftools library (https://pubmed.ncbi.nlm.nih.gov/30341162/) and data analysis was carried out in R v4.0.3 (R Core Team, 2021). To select the most relevant somatic mutations from those previously annotated, only missense and non‐sense SNVs, deletions or mutations with moderate to high predicted impact were considered.

### Statistical analysis

Sample size (*n*) is indicated in the figure legends. Statistical significance between two experimental groups was calculated by unpaired *t* test and in multiple experimental groups also one‐way ANOVA was used with default method corrections using Sigmaplot11. Box charts represent 96% confidence interval, the average is represented by the line inside the box and whiskers correspond to S.D as detailed for each graph in the figure legend.

## Author contributions

HF‐R performed research and analysed data. LH, MJ and LEK performed research and analysed data. M‐CA and HK provided Shigella model and performed related experiments. LB and L‐PF generated and characterized venetoclax‐resistant leukaemia cells. TS and AV performed TCGA analysis. VH generated HCT AKO BOK KO KO cells. XL provided HCT AKO cells. AJG‐S conceived the project and supervised research. AJG‐S and HF‐R wrote the manuscript with the help of all other authors.

## Supporting information



Appendix

Expanded View Figures PDF

Source Data for Expanded View and Appendix

Source Data for Figure 1

Source Data for Figure 2

Source Data for Figure 5

Source Data for Figure 6

## Data Availability

This study includes no data deposited in external repositories.
