# Supplementary material for: BCL‐2‐family protein tBID can act as a BAX‐like effector of apoptosis
Source: EMBO J. 2021 Dec 21;41(2):e108690. doi: 10.15252/embj.2021108690 (PMC8762556; doi:10.15252/embj.2021108690)
Supplement: Supplementary file 1 — Appendix [file EMBJ-41-e108690-s005.pdf]

# Appendix

## Contents

Appendix Figure S1. Untagged tBID behaves like tBID-GFP ........ 2

Appendix Figure S2. tBID-GFP overexpression induces mitochondrial DNA release in HCT AKO cells ... 3

Appendix Figure S3. GFP-BAX and tBID-GFP assemblies and their impact onto mitochondrial structure ........ 4

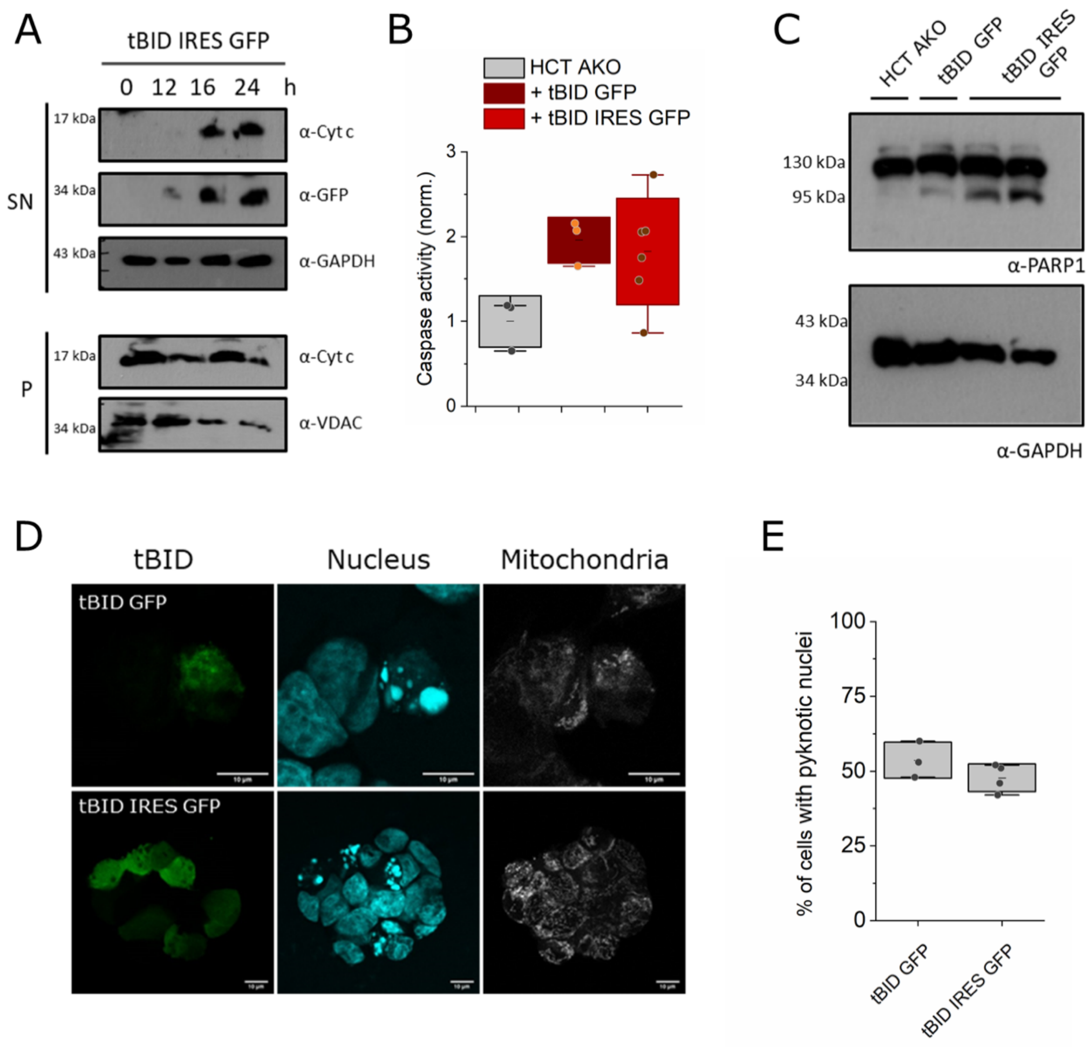


**Appendix Figure S1. Untagged tBID behaves like tBID-GFP**. A) Western Blot of subcellular localization of cyt c of HCT AKO cells expressing tBID-IRES-GFP at different times. B) Caspase3/7 activity induced by tBID-GFP and tBID-IRES-GFP overexpression in HCT AKO cells normalized to untransfected condition. Each dot represents one technical replicate from n=2 independent experiments. C) Representative Western Blot analysis of PARP1 cleavage upon tBID-GFP and tBID-IRES-GFP expression in HCT AKO cells. D) Representative confocal immunofluorescence of tBID-GFP and tBID-IRES-GFP expressed in HCT116 AKO cells showing cellular nuclei. Scale bar 10 µm. E) Quantification of pyknotic nuclei formation in HCT AKO cells expressing tBID-GFP and tBID-IRES-GFP. Each dot corresponds to a technical replicate from n=3 independent experiments with >20 cells per condition per experiment. Error bars represent S.D.

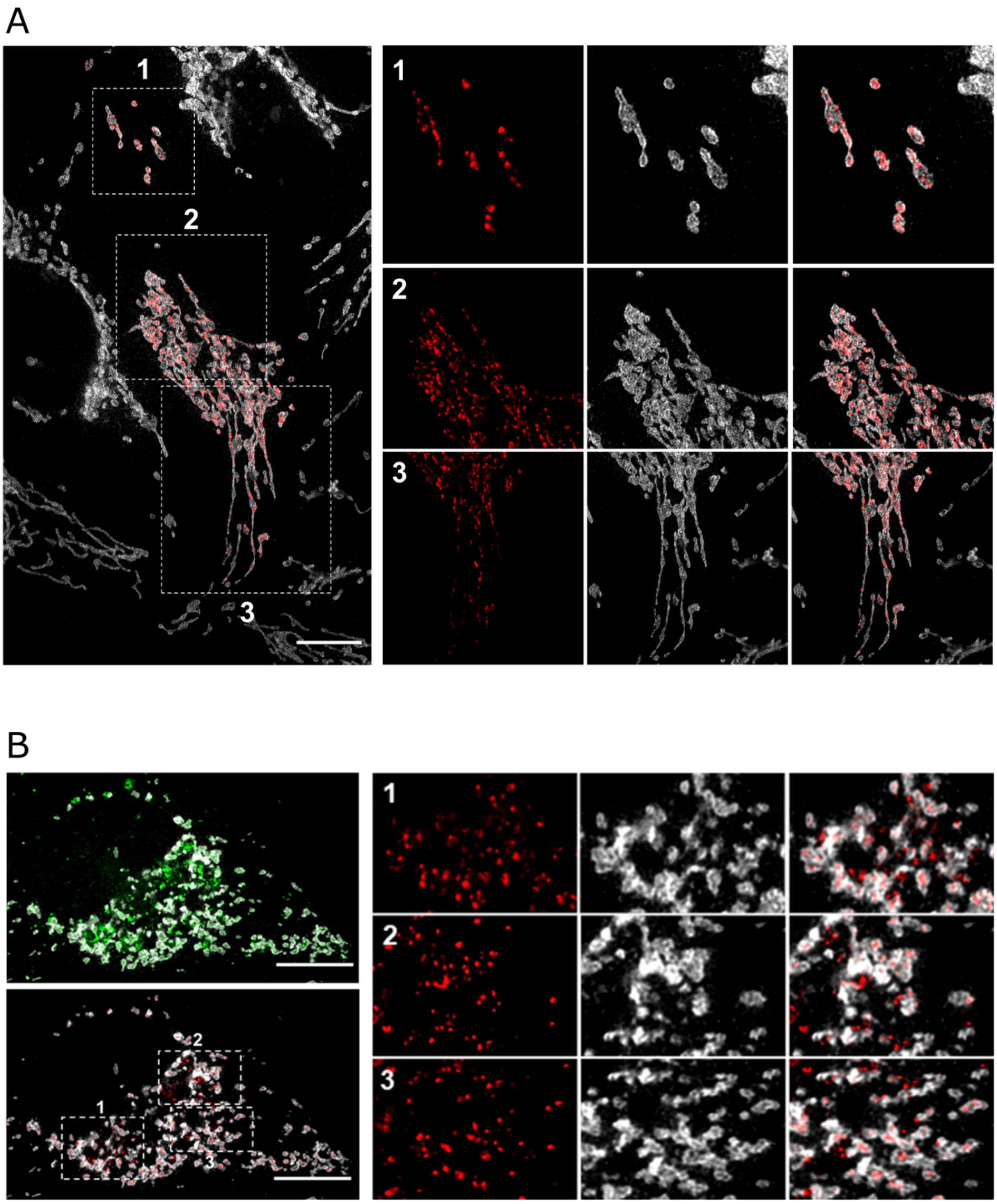


**Appendix Figure S2. tBID-GFP overexpression induces mitochondrial DNA release in HCT AKO cells.** A-B) Representative HyVolution – superresolution images of TFAM-RFP expressing U2OS BAX/BAK DKO cells in the absence (A) and presence of tBID-GFP (B) with COMBO. Scale bar 10 µm. TFAM appears in red, TOM20/mitochondria in grey and tBID-GFP in green.

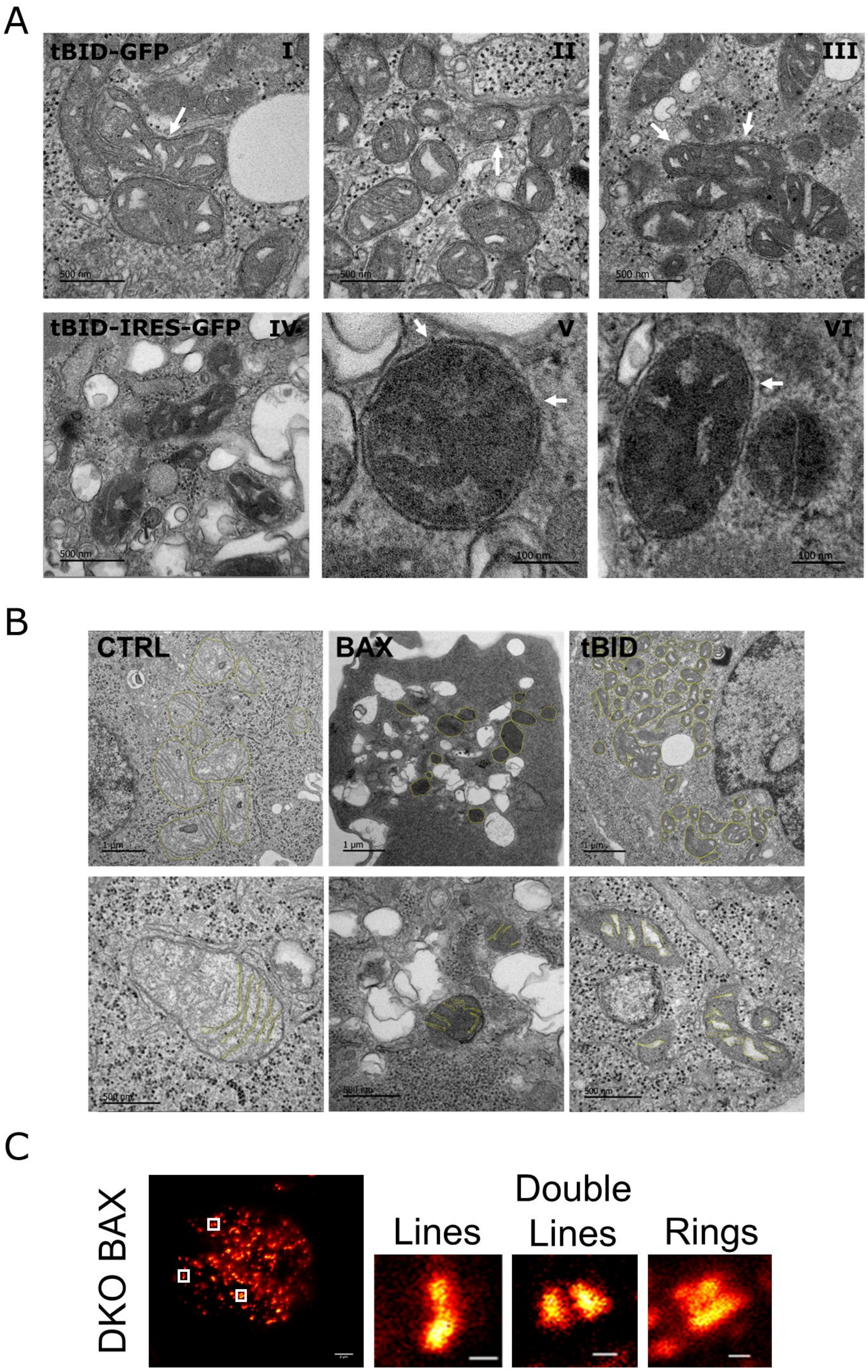


**Appendix Figure S3. GFP-BAX and tBID-GFP assemblies and their impact onto mitochondrial structure.** A) Representative CLEM images of HCT AKO cells transfected tBID-GFP and tBID-IRES-GFP, with membrane disruptions selected with arrows. B) Representative CLEM images of non-transfected HCT AKO cells (CTRL) and HCT AKO cells transfected with GFP-BAX or tBID-GFP, with selected mitochondrial cristae highlighted in yellow. C) Representative STED image of GFP-BAX expressed in HCT DKO cells. Scale bar 1 µm. Zoom-in of the structures of BAX supramolecular assemblies. Scale bar 100 nm.
