# Supplementary material for: BCL‐2‐family protein tBID can act as a BAX‐like effector of apoptosis
Source: EMBO J. 2021 Dec 21;41(2):e108690. doi: 10.15252/embj.2021108690 (PMC8762556; doi:10.15252/embj.2021108690)
Supplement: Supplementary file 2 — Expanded View Figures PDF [file EMBJ-41-e108690-s007.pdf]

Expanded View Figures

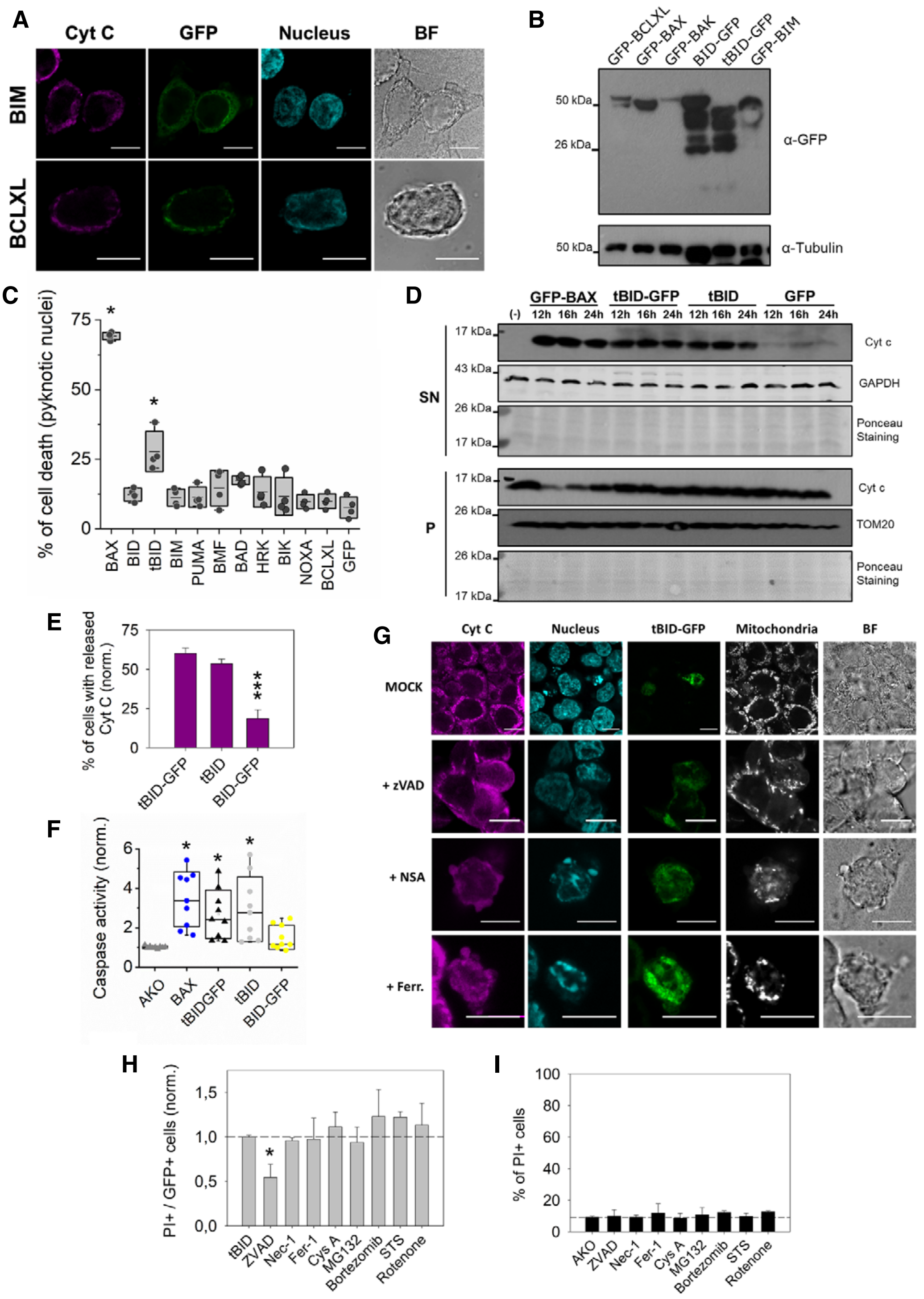

Figure EV1.

 © 2021 The Authors

Figure EV1. tBID, but not other BH3-only proteins or BCLXL, induces apoptosis in HCT AKO cells.

A Representative confocal immunofluorescence and BF images of GFP-BIM and GFP-BCLXL expressed in HCT116 AKO cells showing mitochondrial cytochrome c and cellular nuclei. Scale bar is 10 μm.

B Representative western blot of the expression levels of GFP-tagged BCL2 proteins in HCT AKO cells after 18 h after transfection.

C Percentage of cell death by quantification of pyknotic nuclei formation in HCT AKO cells expressing GFP-tagged BAX, BID, tBID, BIM, PUMA, BMF, BAD, HRK, BIK, NOXA, BCLXL and GFP. $n = 4$ independent experiments with > 50 cells per condition per experiment. $^*P < 0.05$ with respect to GFP condition.

D Western blot of subcellular localization of cyt c of HCT AKO cells expressing GFP-BAX, tBID-GFP and non-tagged tBID.

E Quantification of cyt c release in HCT AKO cells expressing tBID-GFP, tBID and BID-GFP. $n = 3$ independent experiments with > 20 cells per condition per experiment. Unpaired Student's *t*-test $^{***}P < 0.001$ with respect to tBID-GFP condition. Error bars represent SD.

F Caspase3/7 activity induced by GFP-BCL-2 proteins overexpression in HCT AKO cells normalized to untransfected condition. Each dot represents one technical replicate from $n = 3$ independent experiments $^*P < 0.05$ with respect to untransfected condition.

G Representative confocal immunofluorescence and BF images of HCT AKO cells expressing tBID-GFP, and in the presence of ZVAD, NSA and Ferrostatin-1 (Fer-1). Scale bar is 10 μm.

H, I Effect of different cell death-related drugs upon incubation on HCT AKO cells transfected with tBID-GFP, measured as PI^+ cells. For zVAD, Nec-1 and Fer-1 treatment was added 6–8 h after transfection and monitored for additional 6–8 h, whereas for Cys A, MG132, Bortezomib, STS and rotenone, were incubated for 3–4 h after 16–18 h transfection. With > 10,000 cells per condition per experiment. $^*P < 0.05$. Error bars represent SD.

Data information: Unless otherwise stated, in C and F, dots correspond to independent experiments, boxes represent 96% confidence interval, the average is represented by the line inside the box and whiskers correspond to SD. and the statistical significance was assessed by one-way analysis of variance (ANOVA).
Source data are available online for this figure.

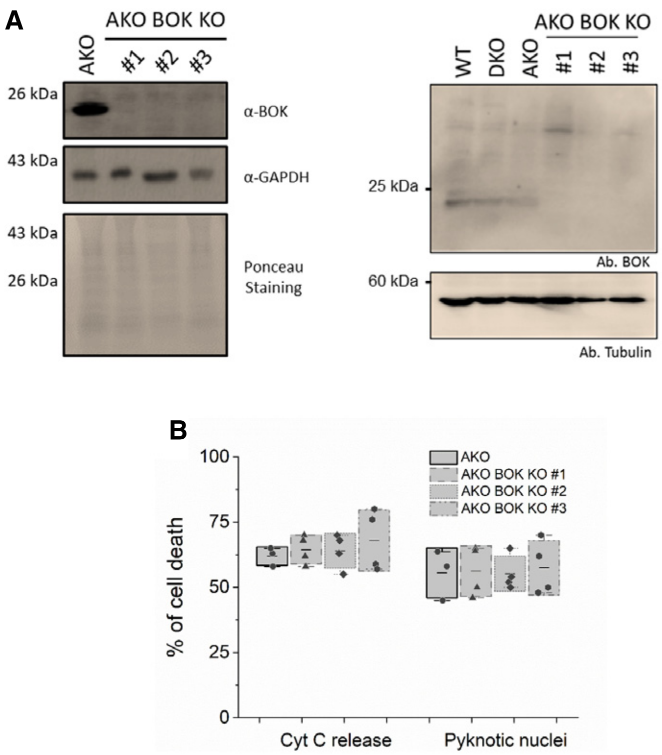

Figure EV2. Endogenous BOK is not required for the activity of tBID to induce MOMP.

A Western blot showing the endogenous levels of BOK in HCT wt, DKO, AKO and in three different HCT AKO BOK KO cell lines, with 120 μg (left) and 40 μg (right) per cellular extract.

B Quantification of the effect of tBID-GFP on cell death, measured as percentage of cells showing released cytochrome c or pyknotic nuclei. Data correspond to at least three independent experiments, $n > 30$ cells per condition per experiment. Dots correspond to independent experiments, boxes represent 96% confidence interval, the average is represented by the line inside the box and whiskers correspond to SD. The statistical significance was assessed by one-way analysis of variance (ANOVA).

Source data are available online for this figure.

© 2021 The Authors

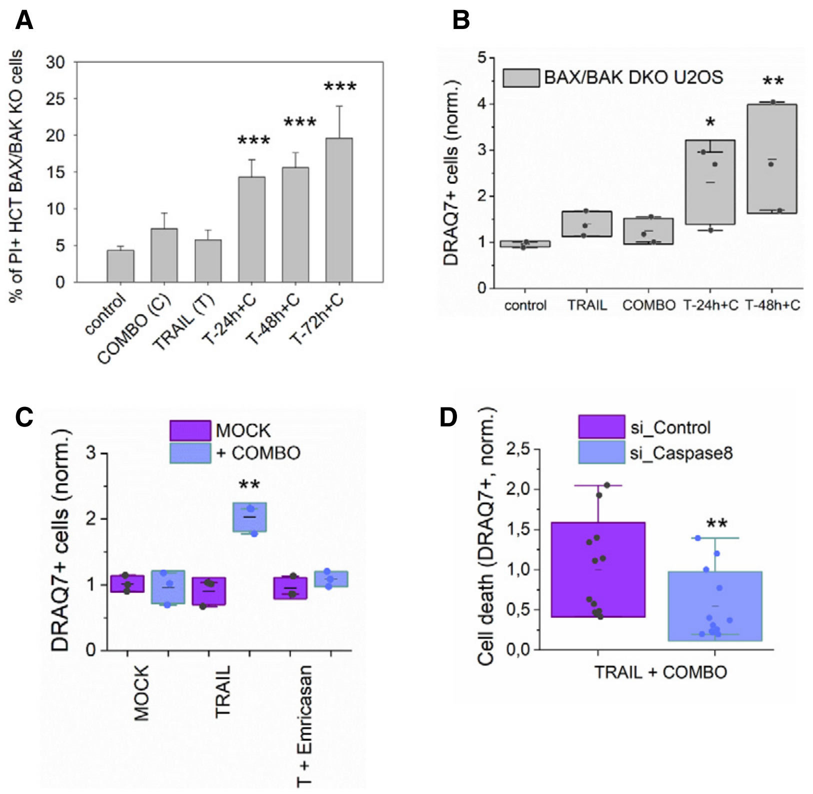

Figure EV3. Cell death induced by combination treatment of TRAIL and COMBO requires BID cleavage.

A Effect of TRAIL (1 μg/ml), COMBO and their combination on HCT DKO transfected, measured as PI^+ cells. $n = 4$ independent experiments with over 10,000 cells per condition per experiment. ***$P < 0.001$ with respect to untreated conditions. Error bars represent SD.

B, C Cell death induced by TRAIL, COMBO and their combination in U2OS DKO (B) and HCT DKO (C), measured as $DRAQ7^+$ cells and normalized to untreated cells. *$P < 0.05$ and **$P < 0.025$ with respect to control condition.

D Cell death induced by TRAIL and COMBO combination in HCT DKO transfected with siRNA control or siRNA for Caspase-8, measured as $DRAQ7^+$ cells and normalized to siControl transfected cells. **$P < 0.025$ with respect to control condition. Unpaired Student's *t*-test.

Data information: In B, C and D, dots correspond to the average value of three technical replicates of three independent experiments. Boxes represent 96% confidence interval, the average is represented by the line inside the box and whiskers correspond to SD and the statistical significance was assessed by one-way analysis of variance (ANOVA).

© 2021 The Authors

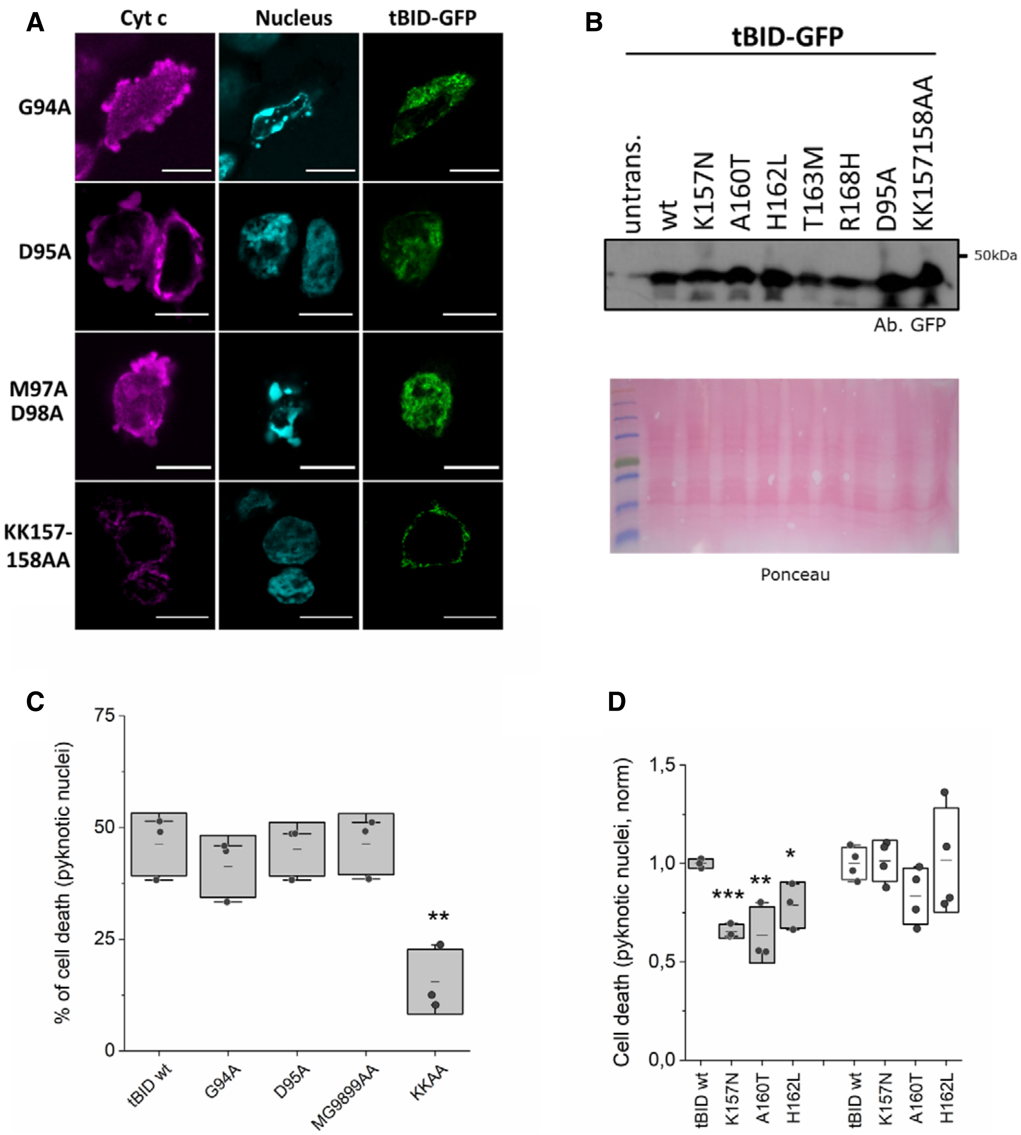

Figure EV4. tBID undergoes apoptosis independently of its BH3 domain and dependent on its α-helix 6.

A Representative confocal immunofluorescence images of tBID-GFP G94A, D95A, M97A/D98A and K157A/K158A mutants expressed in HCT 116 AKO. Cytochrome c (magenta), nuclei (cyan) and tBID-GFP localization (green). Scale bar is 10 μm.

B Western blot of the expression levels of tBID-GFP wt and its mutants when overexpressed in HCT AKO cells.

C Quantification of pyknotic nuclei formation in HCT AKO cells expressing tBID-IRES-GFP wt, G94A, D95A, MG9899AA and KK157158AA mutants. Each dot corresponds to a technical replicate from $n = 3$ independent experiments with > 30 cells per condition per experiment. $^{**}P < 0.025$ with respect to wt condition.

D Quantification of pyknotic nuclei formation in cells expressing tBID-IRES-GFP wt, K157N, A 160 T and H162L mutants (HCT AKO cells (grey) and MEF BID KO (white). Each dot corresponds to a technical replicate with $n > 30$ cells per condition per experiment from 3–4 independent experiments. $^{*}P < 0.05$, $^{**}P < 0.025$ and $^{***}P < 0.001$ with respect to wt condition.

Data information: In C and D, boxes represent 96% confidence interval, the average is represented by the line inside the box and whiskers correspond to SD. The statistical significance was assessed by unpaired Student's *t*-test.
Source data are available online for this figure.

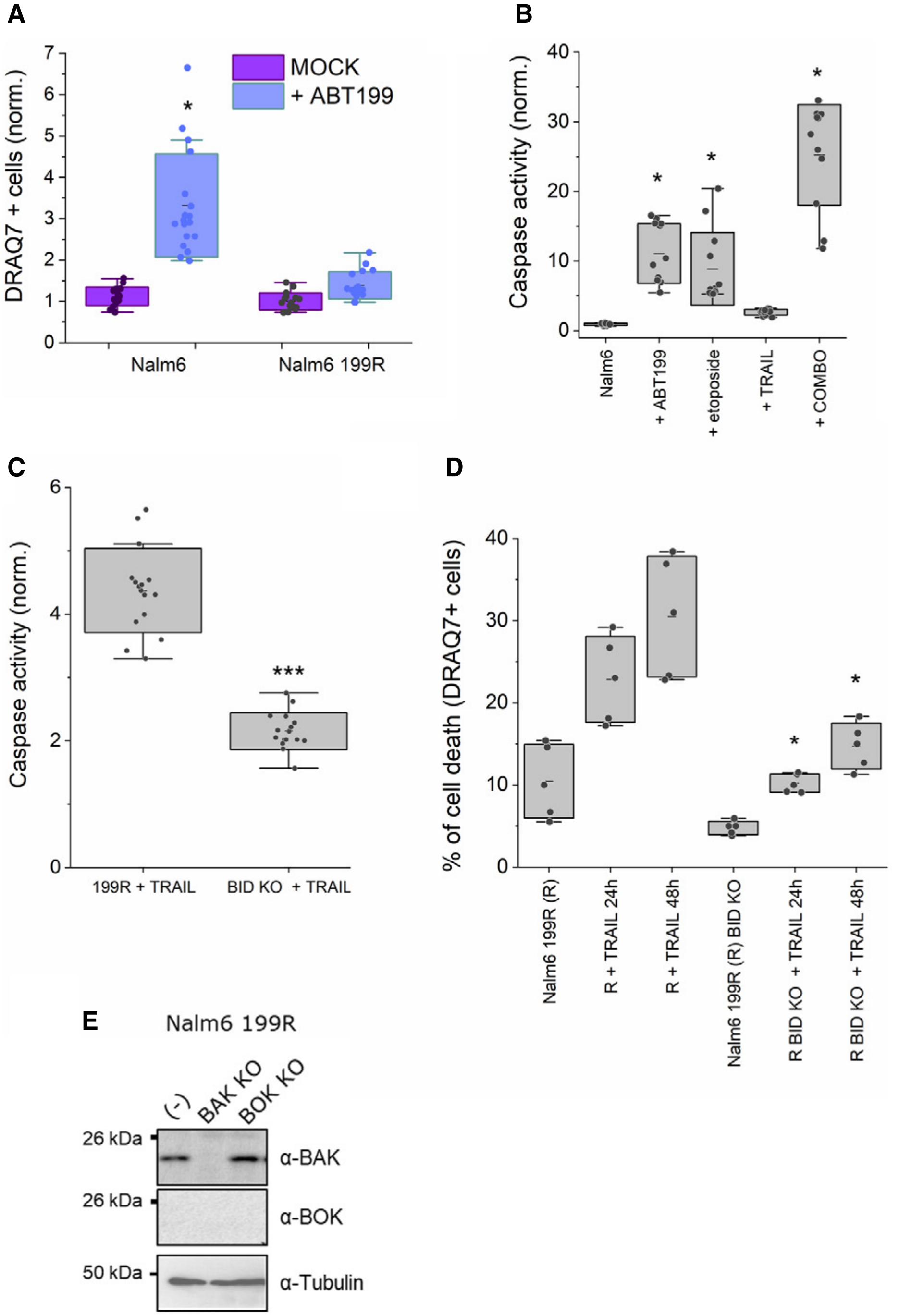

Figure EV5. TRAIL-induced BID cleavage mediates cell death in Nalm6 venetoclax-resistant cells.

A Effect of ABT199 on Nalm6 and Nalm6 199R, measured as DRAQ7^{+} cells. Each dot represents one technical replicate from three independent experiments. *$P < 0.05$ (treated with respect to untreated cells).

B Caspase 3/7 activity in Nalm6 cells when stimulated with indicated drugs and normalized to untreated cells *$P < 0.05$.

C Caspase 3/7 activity in Nalm6 199R and Nalm6 199R BID KO cells stimulated with TRAIL (250 ng/ml). ***$P < 0.001$ (with respect to 199R + TRAIL condition).

D Effect of TRAIL on Nalm6 199R and Nalm6 199R BID KO, measured as DRAQ7^{+} cells at different times with > 10,000 cells per condition per experiment. *$P < 0.05$ (with respect to untreated Nalm6 199R condition) $n = 5$ technical replicates from two independent experiments.

E Western blot showing the endogenous levels of BAK and BOK in Nalm 6 199R, Nalm 6 199R BAK KO and Nalm 6 199R BOK KO cells. Of note, endogenous BOK levels could not be detected by WB in the parental 199R cells.

Data information: Unless otherwise stated in A–D, dots correspond to independent experiments, boxes represent 96% confidence interval, the average is represented by the line inside the box and whiskers correspond to SD. The statistical significance was assessed by one-way analysis of variance (ANOVA).
Source data are available online for this figure.

© 2021 The Authors
