# Supplementary figures and images for: BCL‐2‐family protein tBID can act as a BAX‐like effector of apoptosis
Source: EMBO J. 2021 Dec 21;41(2):e108690. doi: 10.15252/embj.2021108690 (PMC8762556; doi:10.15252/embj.2021108690)

Figure EV4B source data

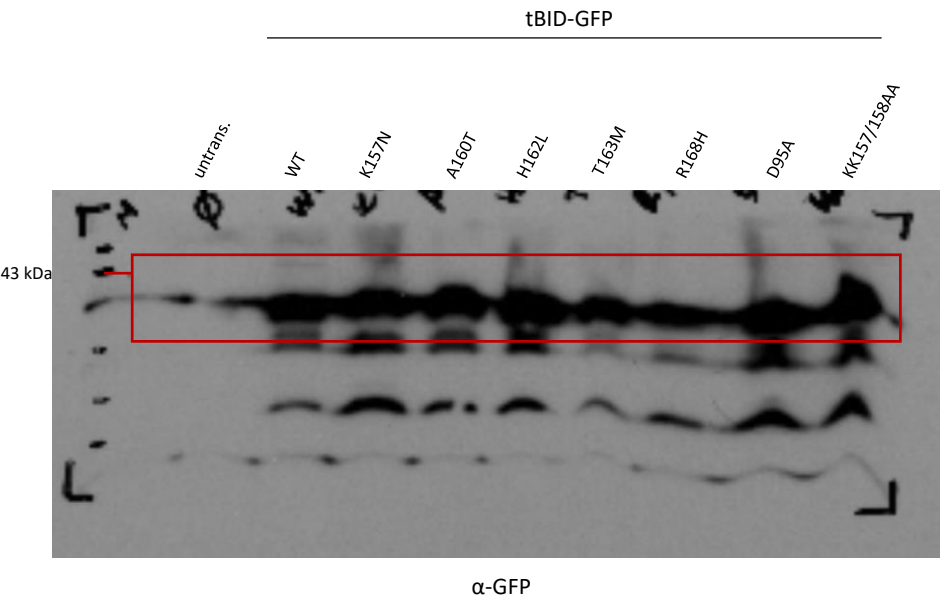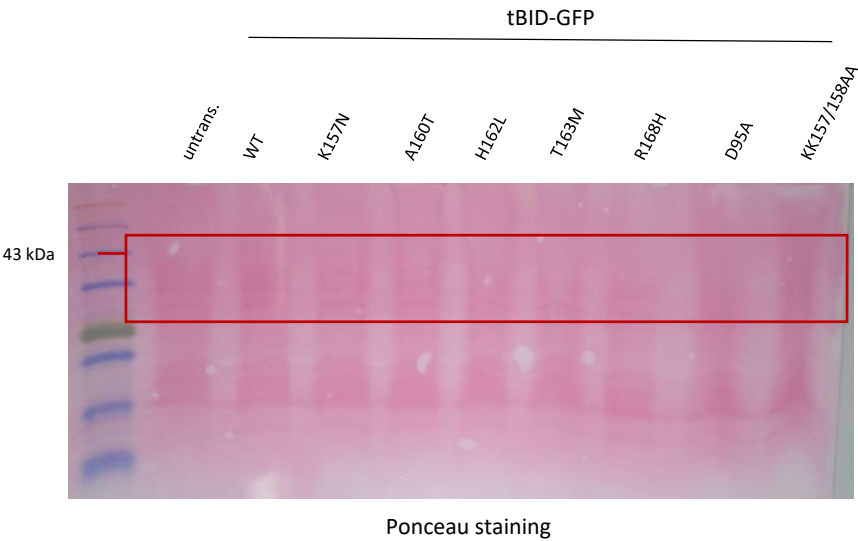

Supplement: Supplementary file 3 — Source Data for Expanded View and Appendix [file EMBJ-41-e108690-s008.zip › EV_and_Appendix_Source_Data/FigureEV4_Source_Data.pdf]

Figure EV5E source data

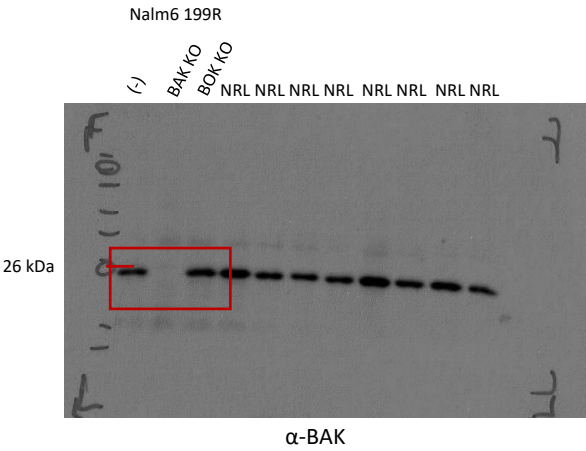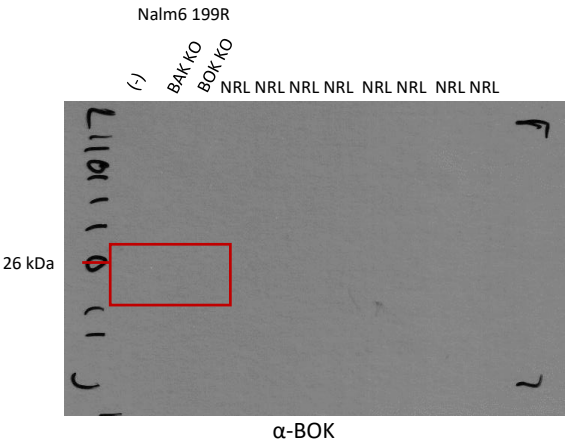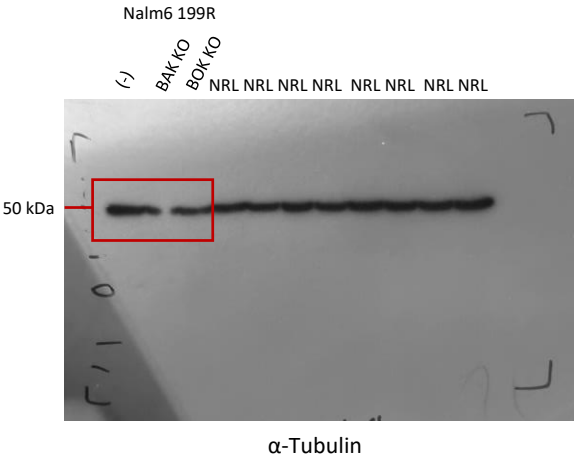

\* NRL = Not Relevant Lane

Supplement: Supplementary file 3 — Source Data for Expanded View and Appendix [file EMBJ-41-e108690-s008.zip › EV_and_Appendix_Source_Data/FigureEV5_Source_Data.pdf]

Figure EV2A source data

#1

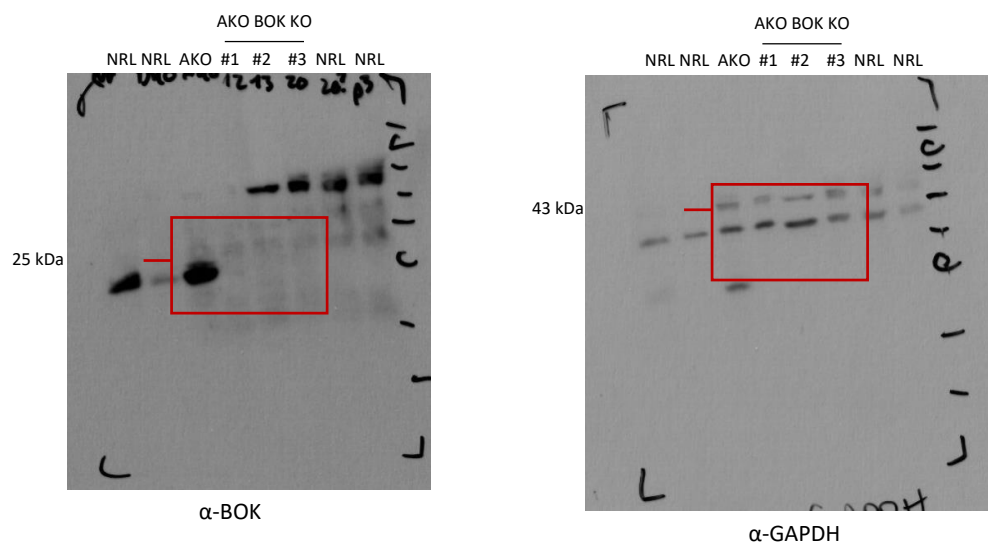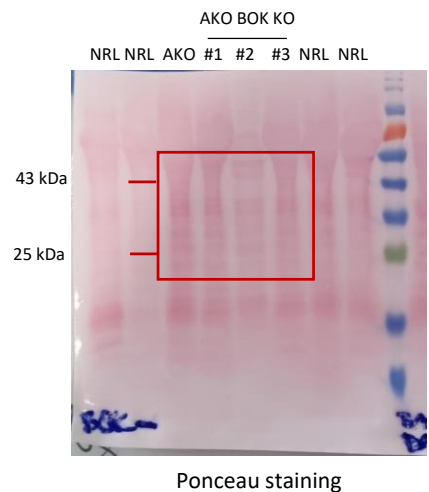

#2

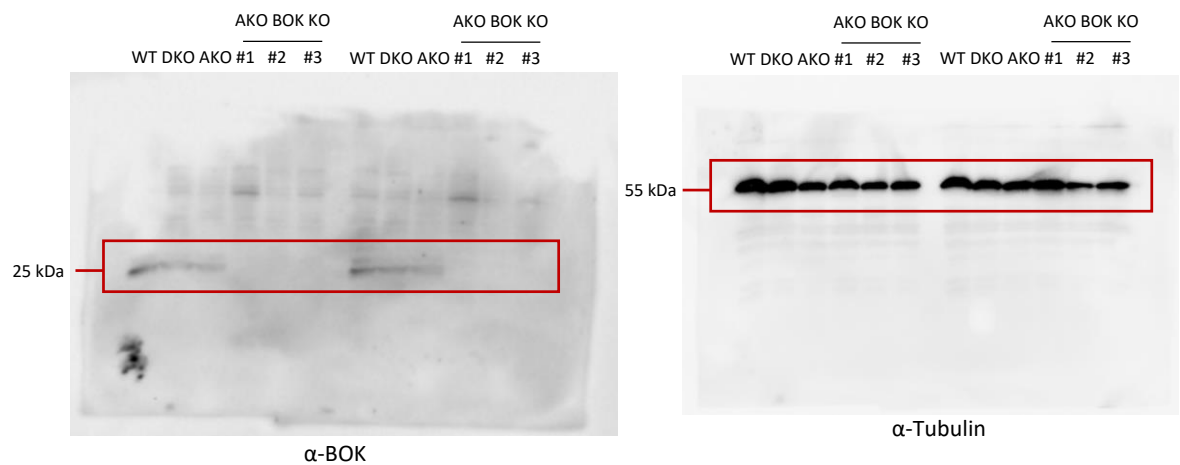

Supplement: Supplementary file 3 — Source Data for Expanded View and Appendix [file EMBJ-41-e108690-s008.zip › EV_and_Appendix_Source_Data/FigureEV2_Source_Data.pdf]

Appendix figure S1A source data

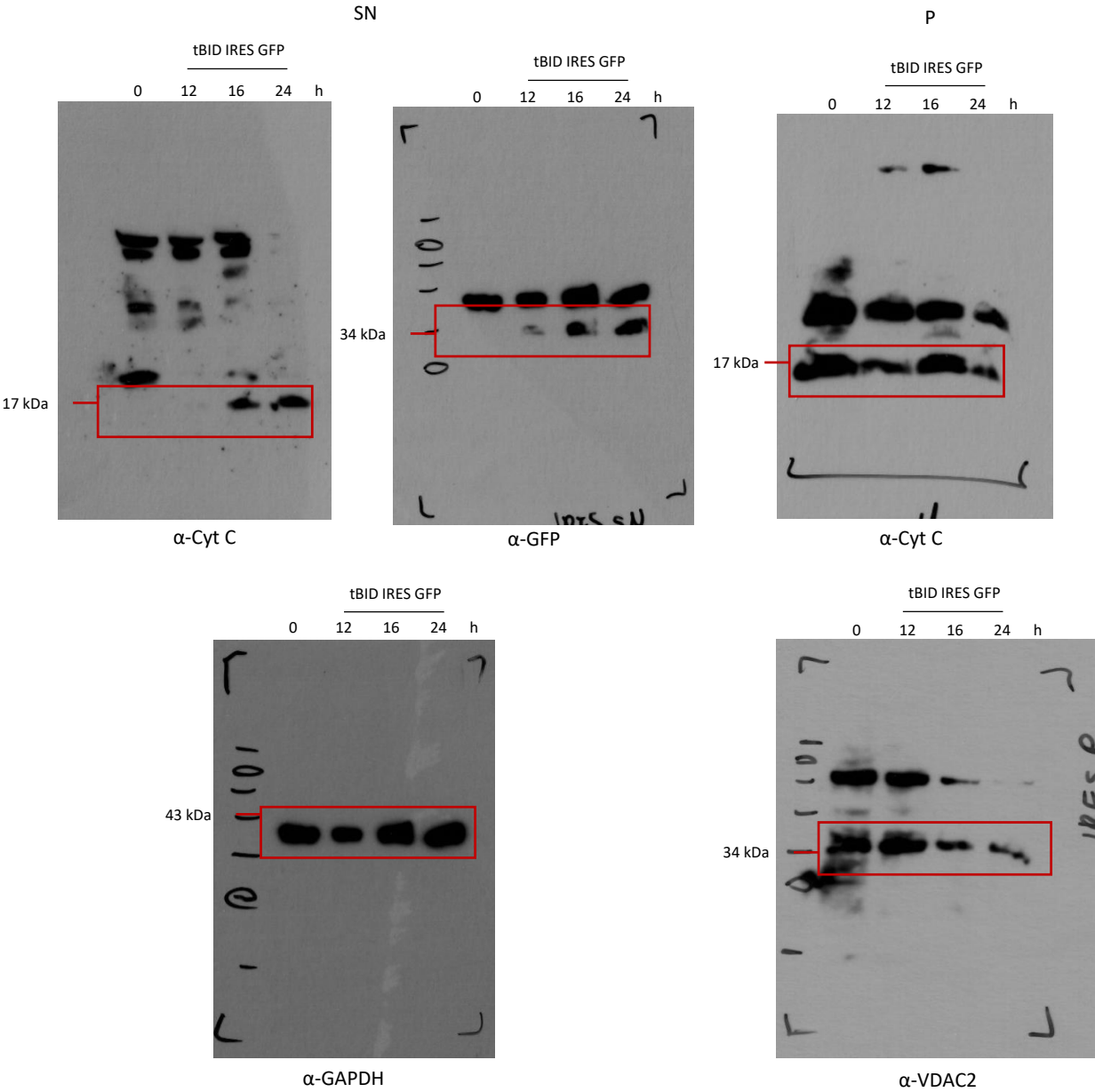

Appendix figure S1C source data

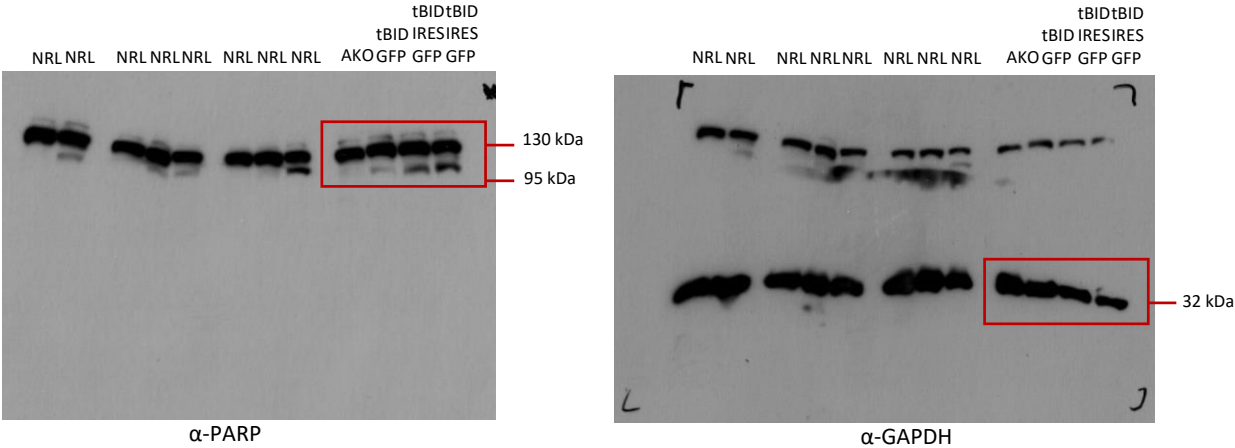

\* NRL = Not Relevant Lane

Supplement: Supplementary file 3 — Source Data for Expanded View and Appendix [file EMBJ-41-e108690-s008.zip › EV_and_Appendix_Source_Data/Source_Data_Appendix_FigS1.pdf]

Figure EV1B source data

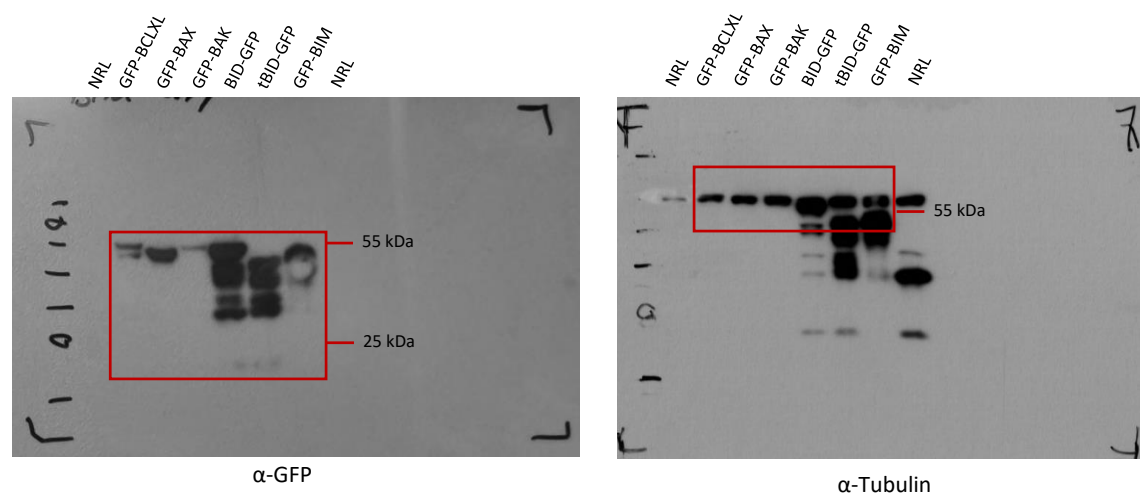

Figure EV1D source data

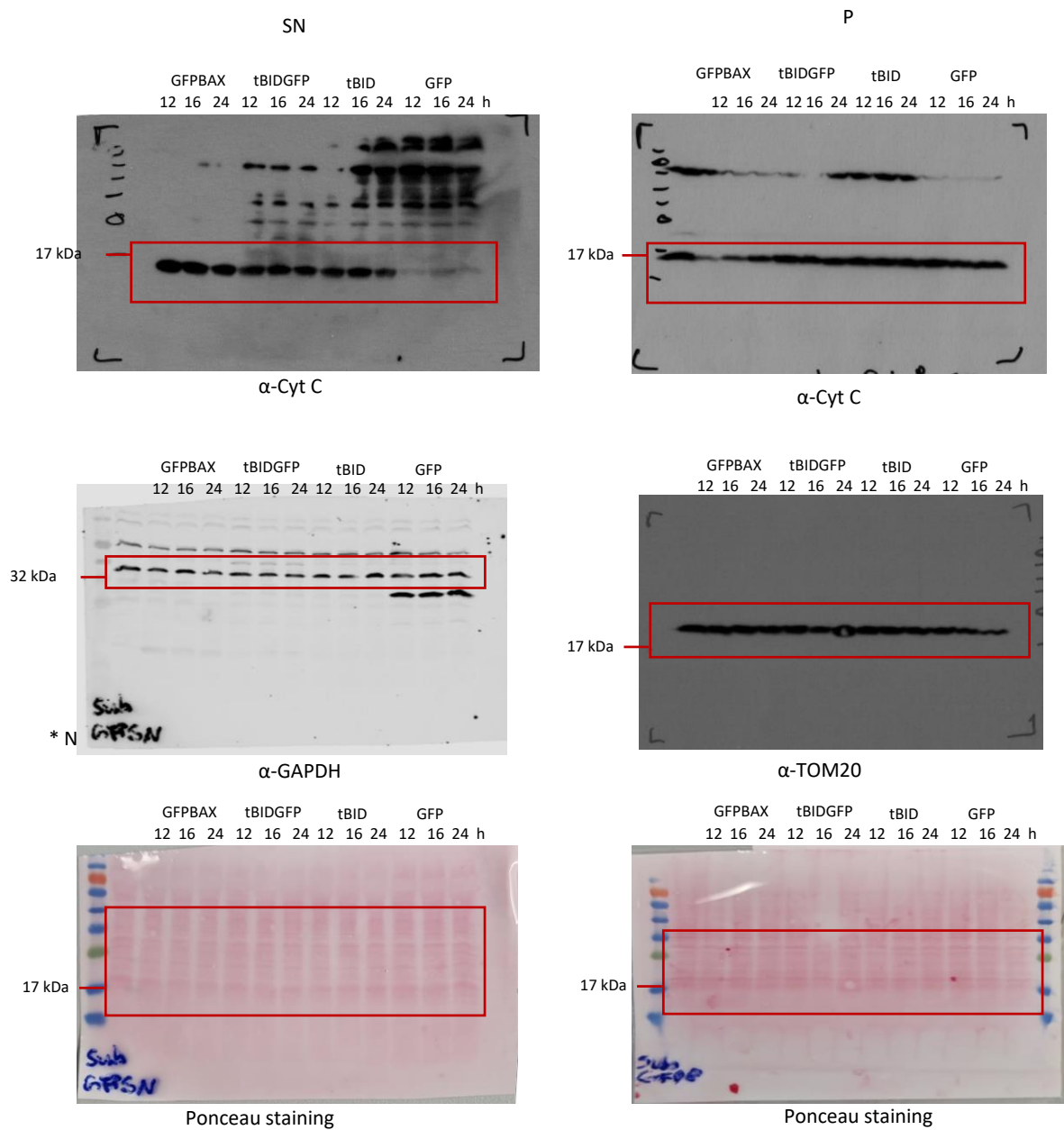

Supplement: Supplementary file 3 — Source Data for Expanded View and Appendix [file EMBJ-41-e108690-s008.zip › EV_and_Appendix_Source_Data/FigureEV1_Source_Data.pdf]

Figure 1C source data

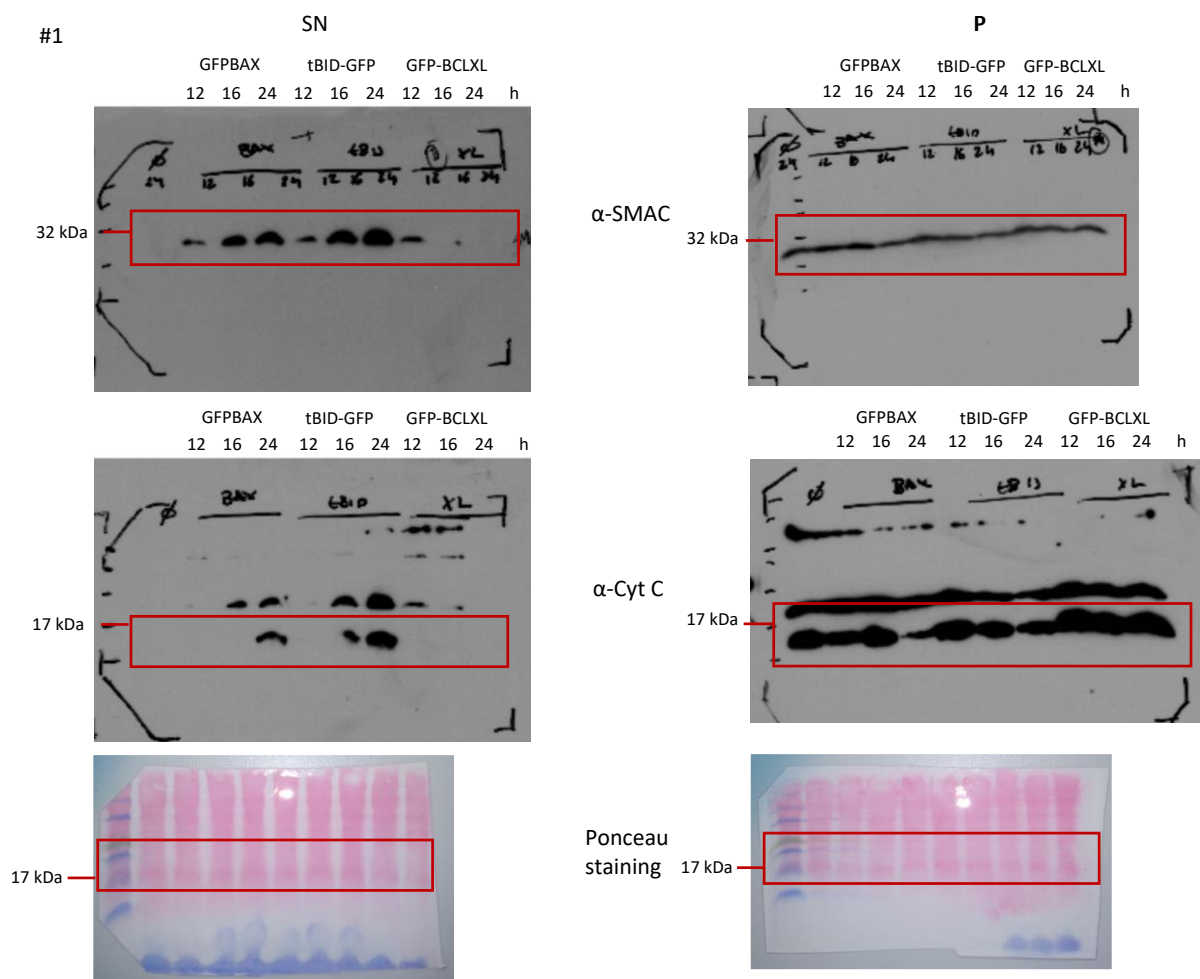

Figure 1E source data

#1

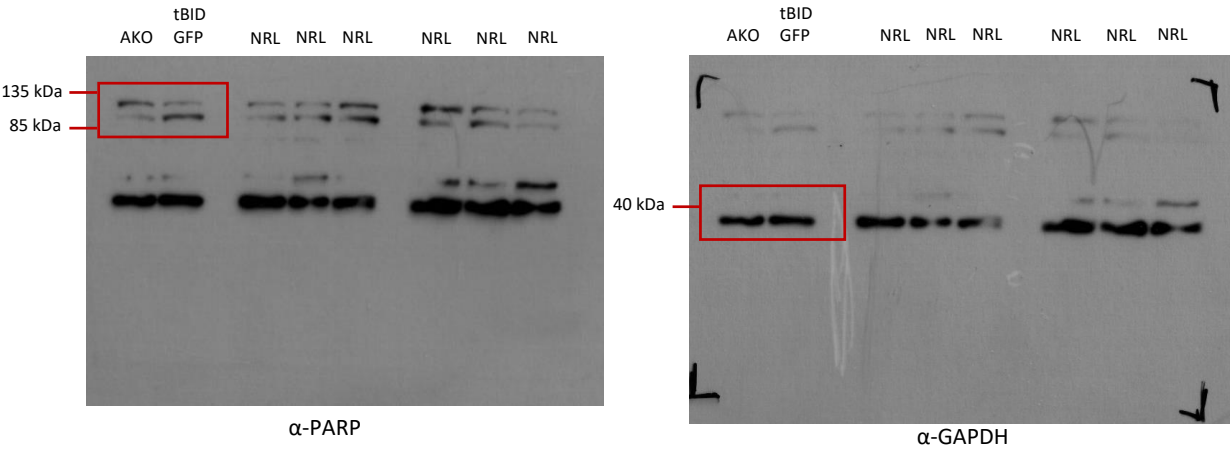

#2

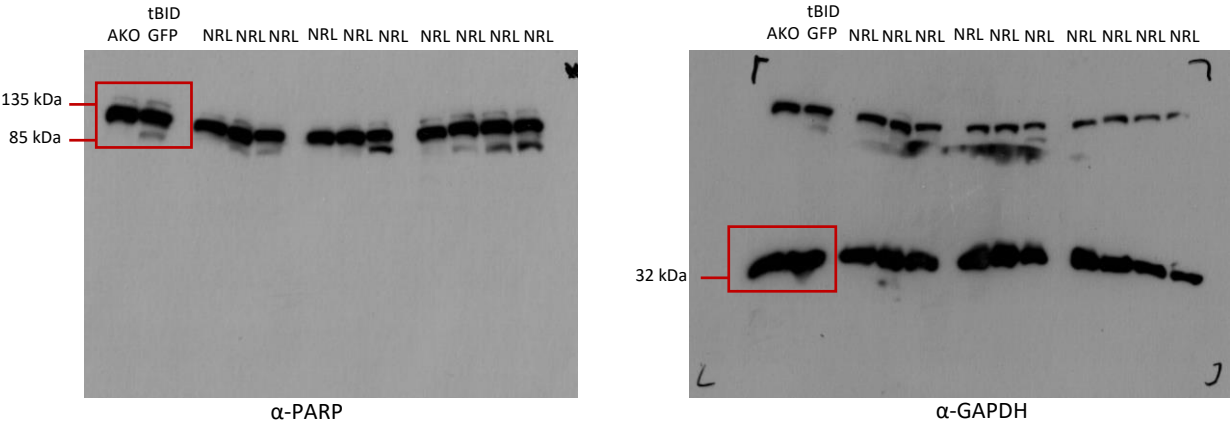

#3

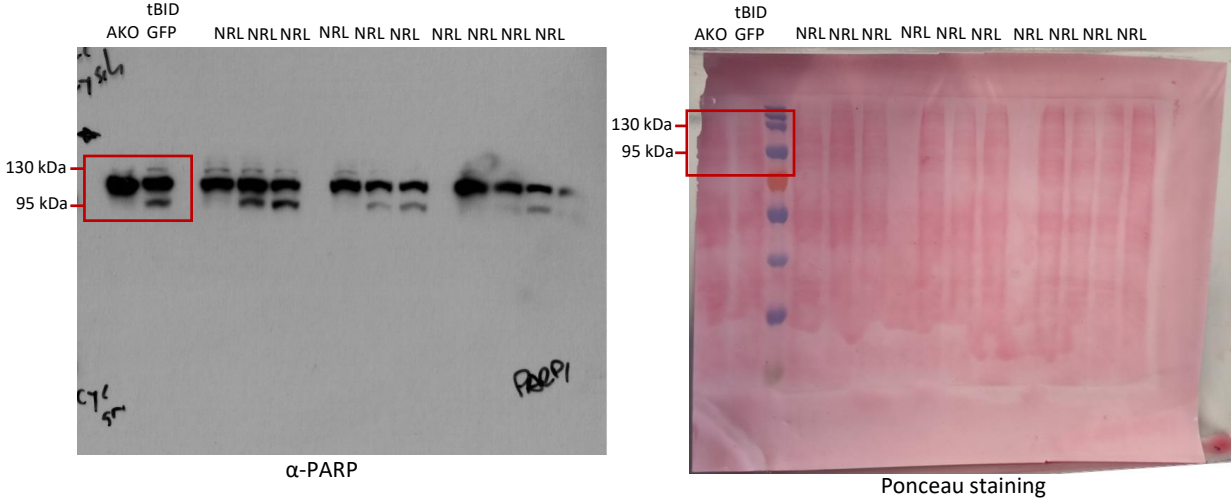

\* NRL = Not Relevant Lane

Supplement: Supplementary file 4 — Source Data for Figure 1 [file EMBJ-41-e108690-s004.pdf]

Figure 2D source data

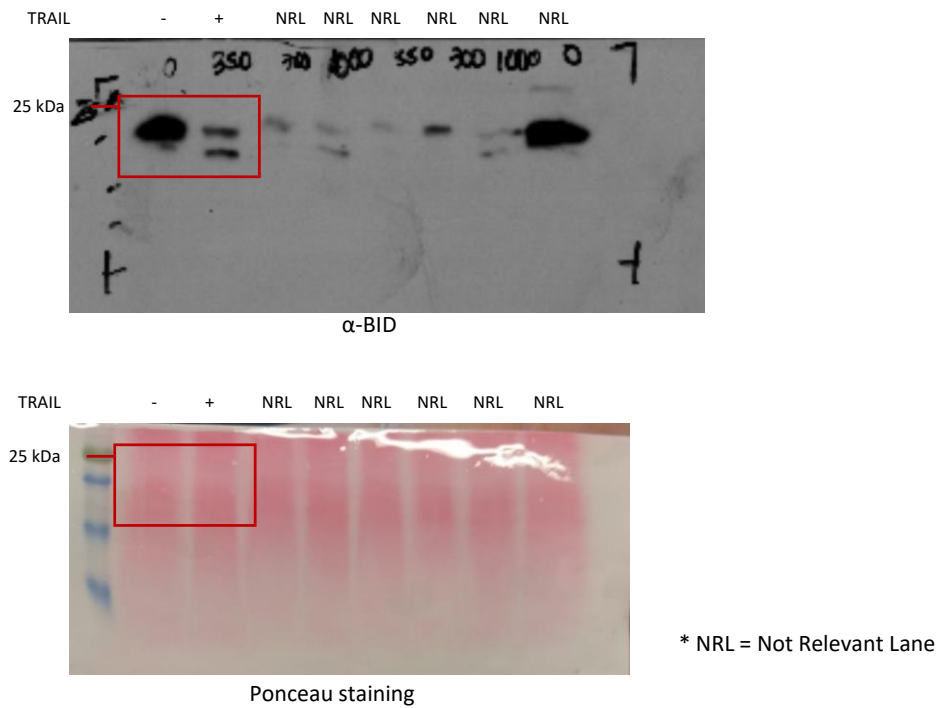

Figure 2G source data

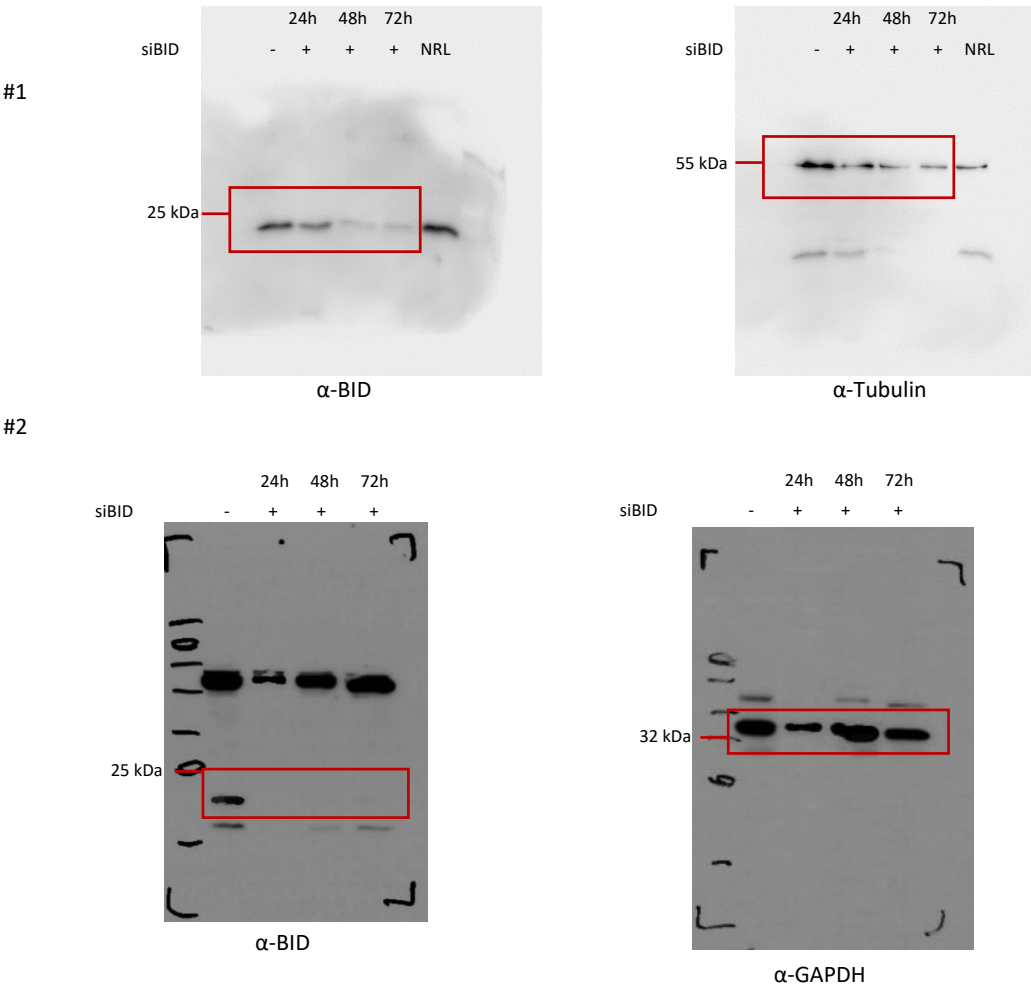

Figure 2H source data

#1

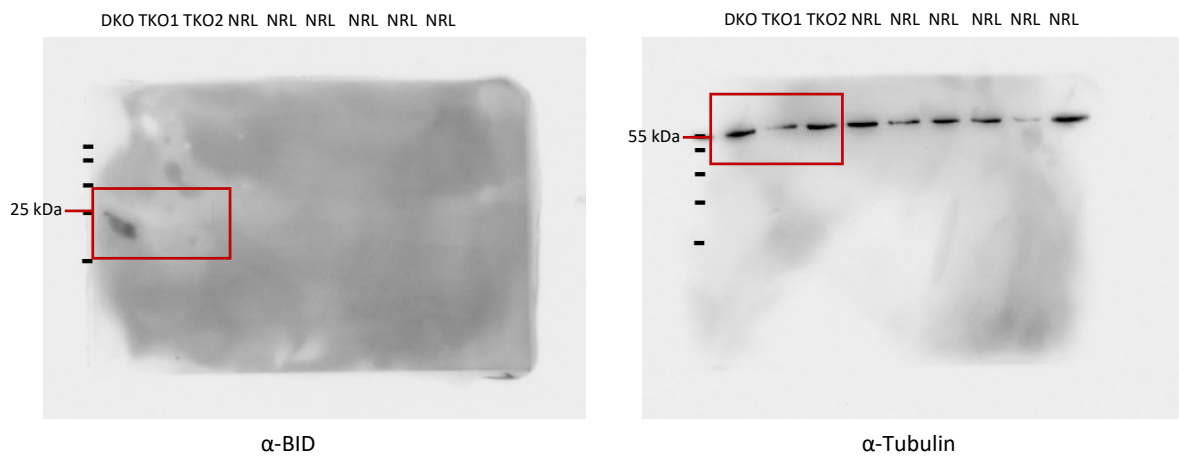

#2

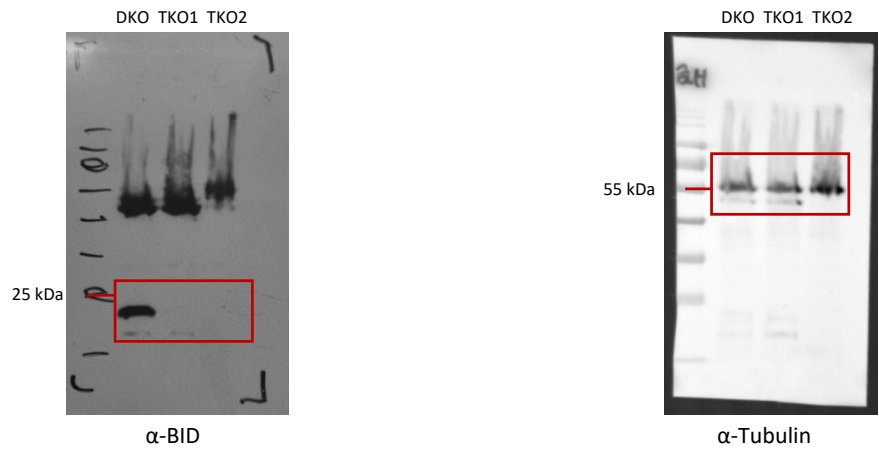

\* NRL = Not Relevant Lane

Supplement: Supplementary file 5 — Source Data for Figure 2 [file EMBJ-41-e108690-s001.pdf]
