# Supplementary material for: BCL‐2‐family protein tBID can act as a BAX‐like effector of apoptosis
Source: EMBO J. 2021 Dec 21;41(2):e108690. doi: 10.15252/embj.2021108690 (PMC8762556; doi:10.15252/embj.2021108690)

Figure 5A source data

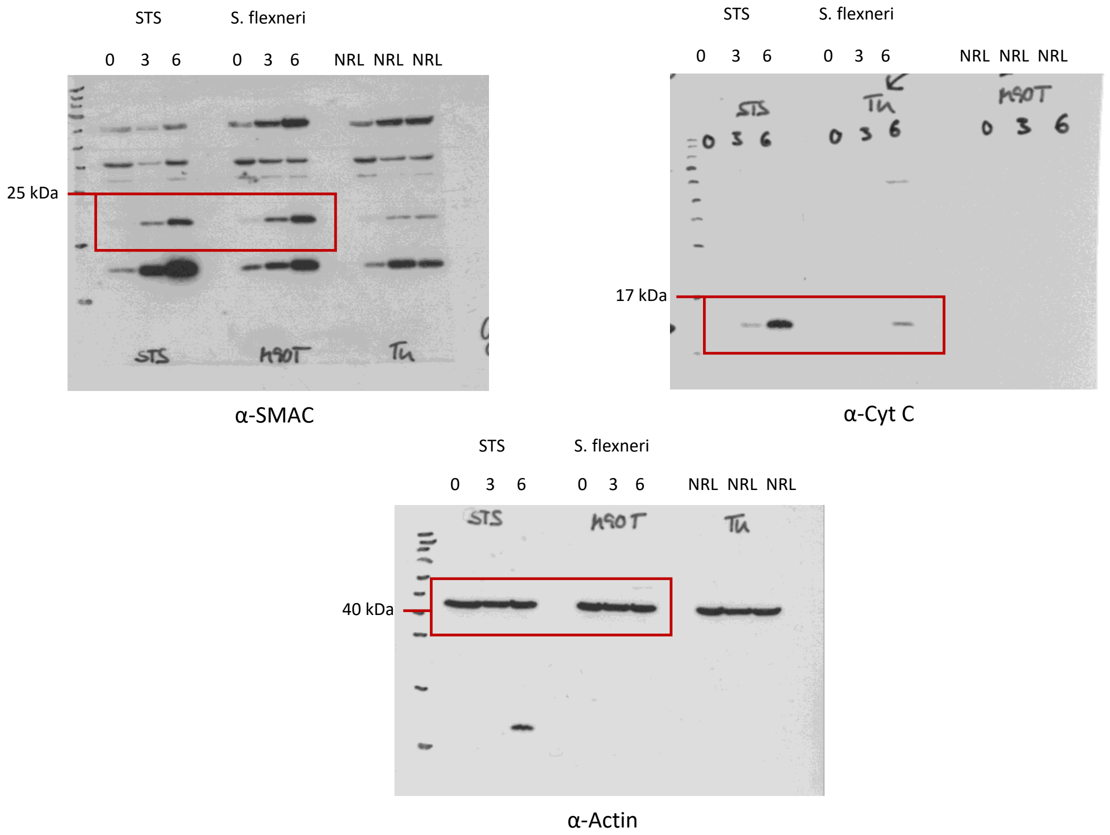

Figure 5B source data

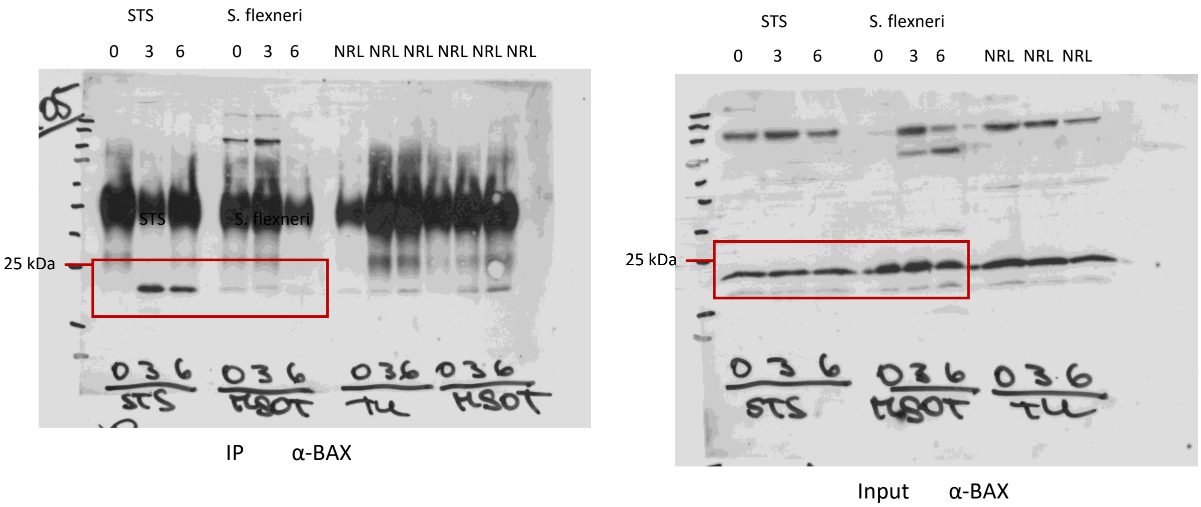

* NRL = Not Relevant Lane

Figure 5C source data

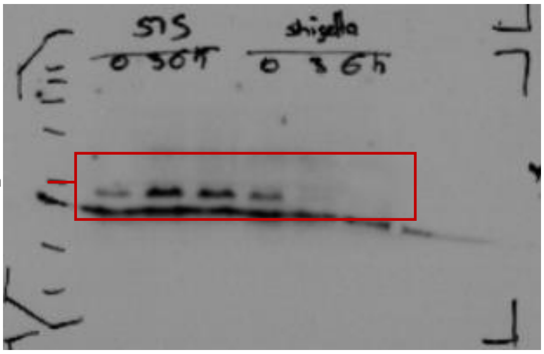

IP α-BAK

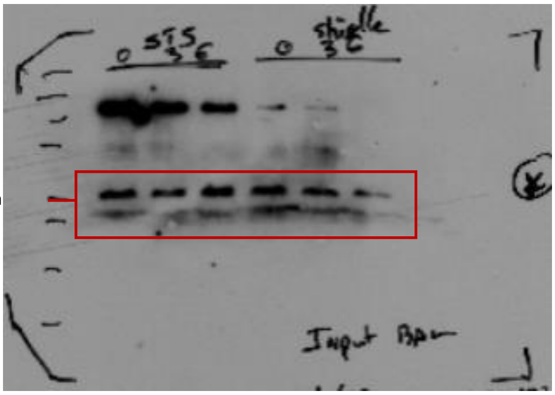

Input α-BAK

Figure 5D source data

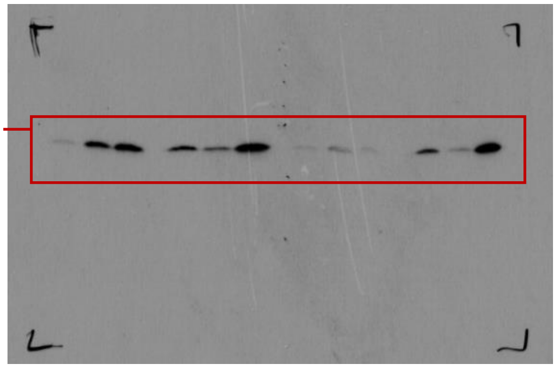

α-SMAC

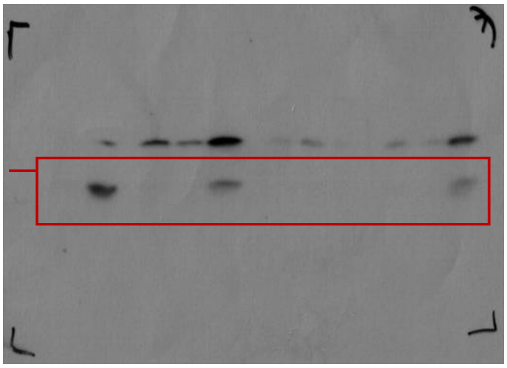

α-Cyt C

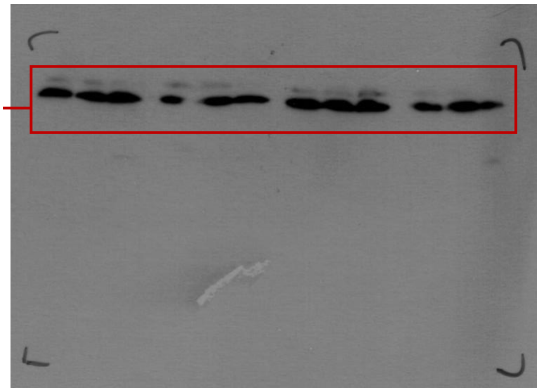

α-GAPDH

Figure 5E source data

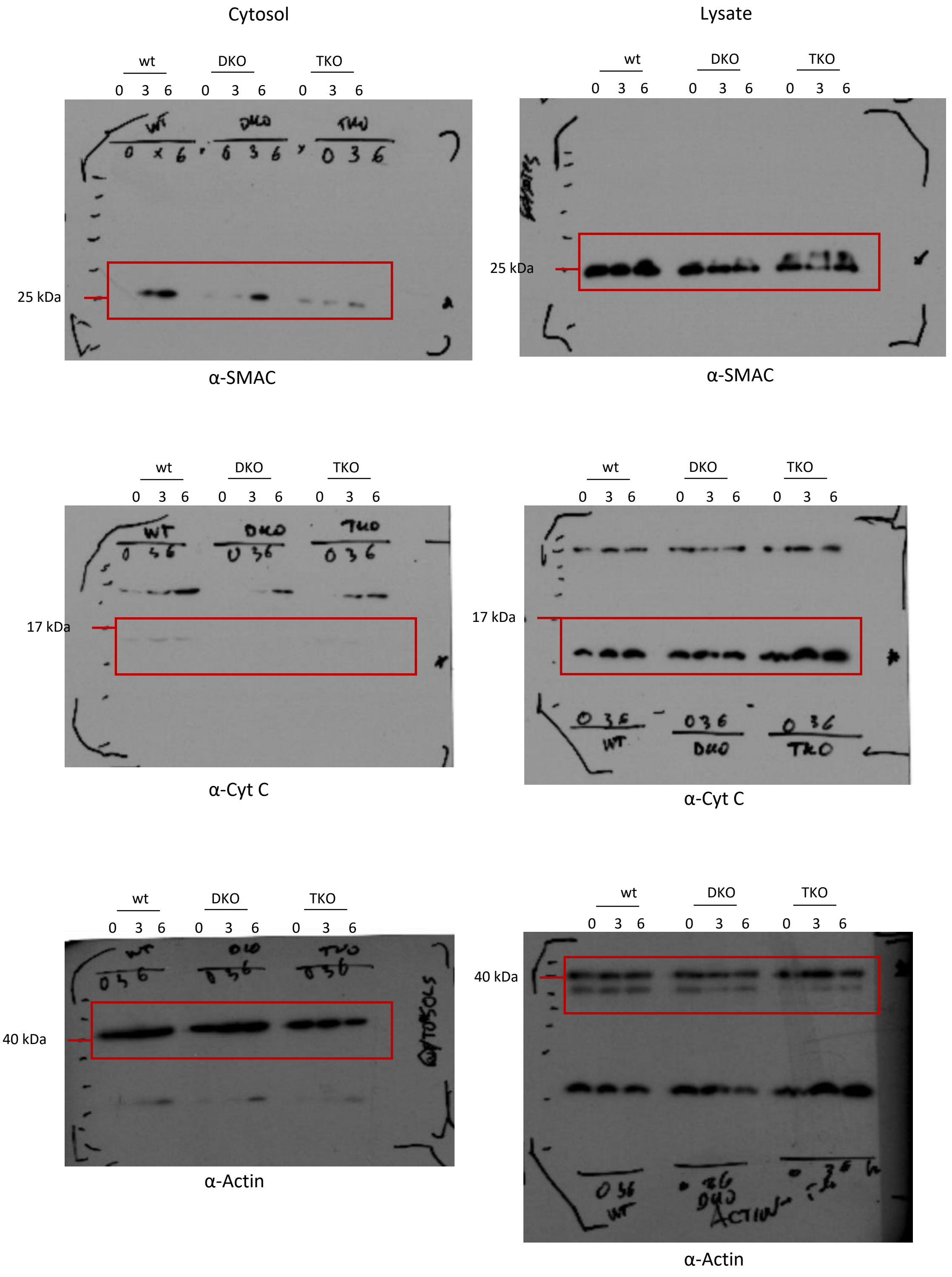

Supplement: Supplementary file 6 — Source Data for Figure 5 [file EMBJ-41-e108690-s002.pdf]
