# Supplementary material for: BCL‐2‐family protein tBID can act as a BAX‐like effector of apoptosis
Source: EMBO J. 2021 Dec 21;41(2):e108690. doi: 10.15252/embj.2021108690 (PMC8762556; doi:10.15252/embj.2021108690)
Supplement: Supplementary file 7 — Source Data for Figure 6 [file EMBJ-41-e108690-s003.pdf]

Figure 6C source data

#1

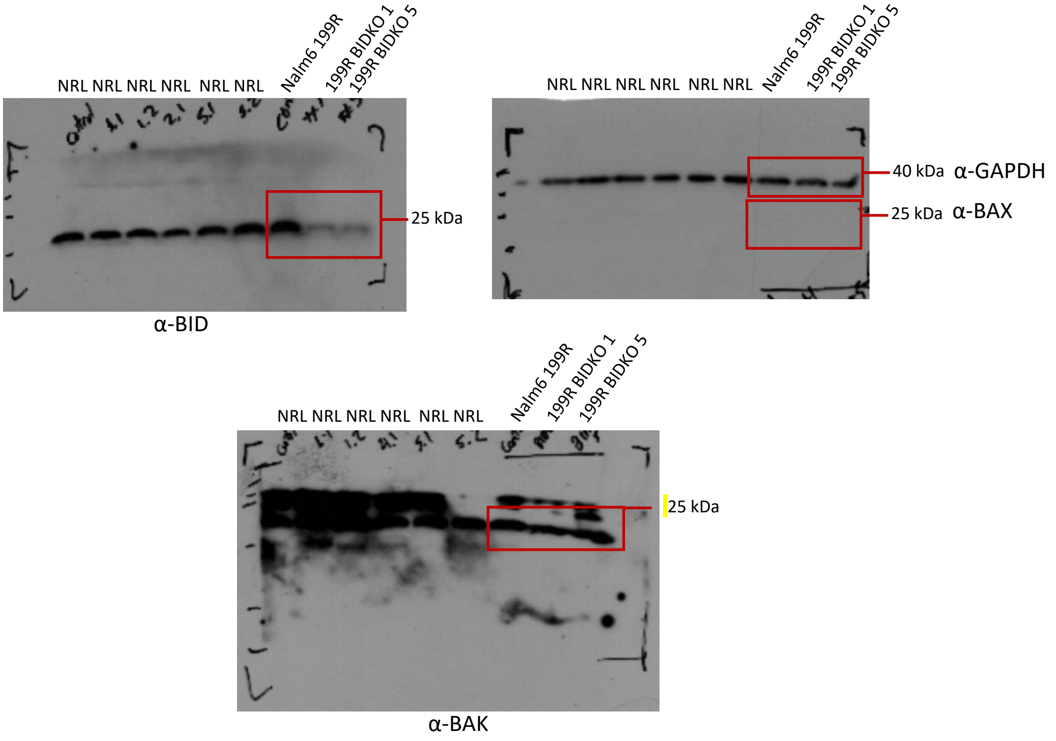

* NRL = Not Relevant Lane

#2

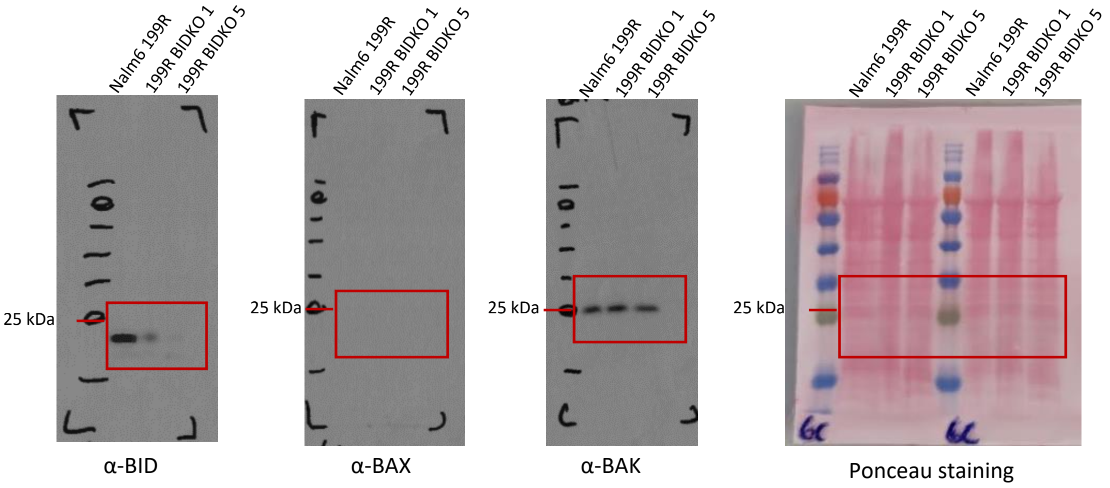

Figure 6F source data

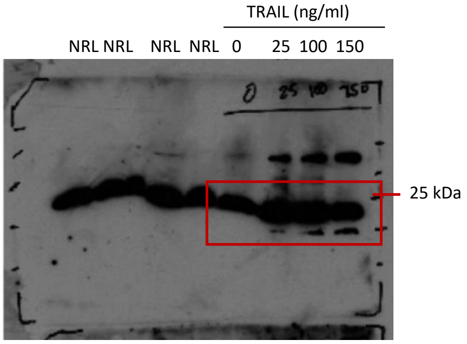

α-BID

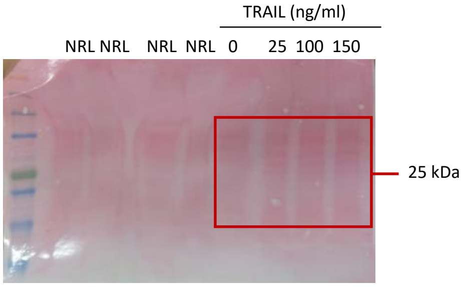

Ponceau staining

Figure 6G source data

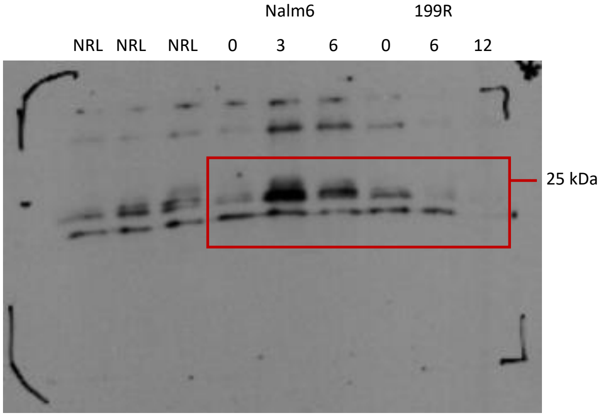

IP α-BAK

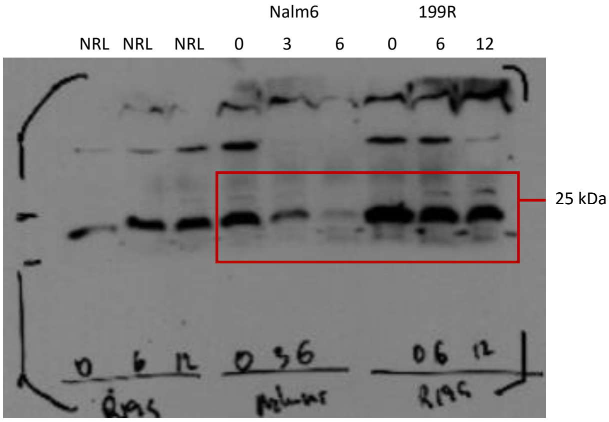

Input α-BAK

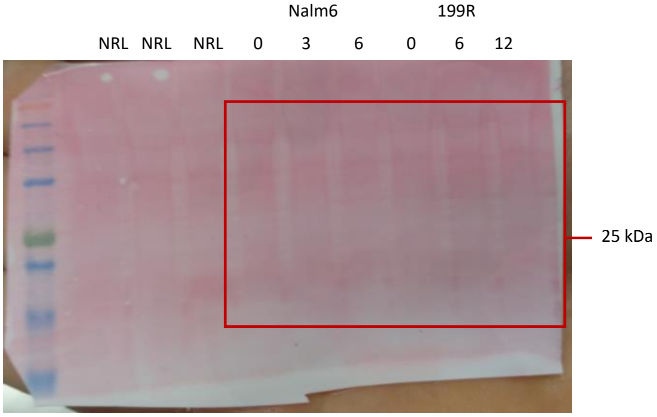

Ponceau staining

* NRL = Not Relevant Lane
