# Supplementary material for: BCL‐2‐family protein tBID can act as a BAX‐like effector of apoptosis
Source: EMBO J. 2021 Dec 21;41(2):e108690. doi: 10.15252/embj.2021108690 (PMC8762556; doi:10.15252/embj.2021108690)
Supplement: Supplementary file 8 [file EMBJ-41-e108690-s006.pdf]

THE EMBO JOURNAL

BCL-2-family protein tBID can act as a BAX-like effector of apoptosis

Hector Flores-Romero, Lisa Hohorst, Malina John, Marie-Christine Albert, Louise King, Laura Beckmann, Tamás Szabó, Vanessa Hertlein, Xu Luo, Andreas Villunger, Lukas Frenzel, Hamid Kashkar, and Ana Garcia-Saez
DOI: 10.15252/embj.2021108690

Corresponding author(s): Ana Garcia-Saez (ana.garcia@uni-koeln.de)

Review Timeline:

Submission Date:	10th May 21
Editorial Decision:	31st May 21
Revision Received:	2nd Sep 21
Editorial Decision:	29th Oct 21
Revision Received:	19th Nov 21
Accepted:	22nd Nov 21

Editor: Karin Dumstrei

Transaction Report:

(Note: With the exception of the correction of typographical or spelling errors that could be a source of ambiguity, letters and reports are not edited. Depending on transfer agreements, referee reports obtained elsewhere may or may not be included in this compilation. Referee reports are anonymous unless the Referee chooses to sign their reports.)

1st Editorial Decision 31st May 2021

Dear Ana,

Thank you for submitting your manuscript entitled "tBID can act as a BAX-like effector of apoptosis" [EMBOJ-2021-108690] to The EMBO Journal. Your study has now been assessed by three reviewers, whose reports are enclosed below.

While the referees find your study potentially interesting, they also raise several critical points that need to be addressed before they can support publication here.

Given the overall interest of your study, I am pleased to invite submission of a manuscript revised as indicated in the reports attached herein. I would like to point it out that addressing all referees' points in a conclusive manner, as well as a strong support from the reviewers, would be essential for publication in The EMBO Journal.

I should also add that it is our policy to allow only a single round of major revision. Therefore, acceptance of your manuscript will depend on the completeness of your responses in this revised version.

We generally grant three months as standard revision time. As we are aware that many laboratories cannot function at full capacity owing to the COVID-19 pandemic, we may relax this deadline. Also, we have decided to apply our 'scooping protection policy' to the time span required for you to fully revise your manuscript and address the experimental issues highlighted herein. Nevertheless, please inform us as soon as a paper with related content is published elsewhere.

When preparing your letter of response to the referees' comments, please bear in mind that this will form part of the Review Process File and will therefore be made available online. For more details on our Transparent Editorial Process, please visit our website: http://emboj.embopress.org/about#Transparent_Process

Before submitting your revised manuscript, deposit any primary datasets and computer code produced in this study in an appropriate public database (see http://msb.embopress.org/authorguide#dataavailability). Please remember to provide a reviewer password, in case such datasets are not yet public. The accession numbers and database names should be listed in a formal "Data Availability" section (placed after Materials & Method). Provide a "Data availability" section even if there are no primary datasets produced in the study.

I thank you again for the opportunity to consider this work for publication and look forward to your revision.

Best regards,

Elisabetta

Elisabetta Argenzio, PhD
Editor
The EMBO Journal

Instructions for preparing your revised manuscript:

Please make sure you upload a letter of response to the referees' comments together with the revised manuscript.

Please also check that the title and abstract of the manuscript are brief, yet explicit, even to non-specialists.

When assembling figures, please refer to our figure preparation guideline in order to ensure proper formatting and readability in print as well as on screen:
https://bit.ly/EMBOPressFigurePreparationGuideline

IMPORTANT: When you send the revision we will require
- a point-by-point response to the referees' comments, with a detailed description of the changes made (as a word file).
- a word file of the manuscript text.
- individual production quality figure files (one file per figure)
- a complete author checklist, which you can download from our author guidelines (https://www.embopress.org/page/journal/14602075/authorguide).
- Expanded View files (replacing Supplementary Information)

Please see out instructions to authors
https://www.embopress.org/page/journal/14602075/authorguide#expandedview

Please remember: Digital image enhancement is acceptable practice, as long as it accurately represents the original data and conforms to community standards. If a figure has been subjected to significant electronic manipulation, this must be noted in the figure legend or in the 'Materials and Methods' section. The editors reserve the right to request original versions of figures and the original images that were used to assemble the figure.

Further information is available in our Guide For Authors: https://www.embopress.org/page/journal/14602075/authorguide

Revision to The EMBO Journal should be submitted online within 90 days, unless an extension has been requested and approved by the editor; please click on the link below to submit the revision online before 29th Aug 2021:

https://emboj.msubmit.net/cgi-bin/main.plex

Referee #1:

BID is widely considered to be the precursor a pro-apoptotic BH3-only protein, that can for example be activated by caspase-8 mediated proteolysis in cells treated with agonists of death receptors (e.g. Fas ligand, TNF or TRAIL). However, it has long been known that the structure of BID closely resembles the structures of BAX, BAK (and BOK). This may indicate that BID, like BAX and BAK, could function as multi-BH domain effector of apoptosis, and not only as the precursor for tBID.
In this manuscript, Hector Flores-Romero and colleagues present data from biochemical and cell biology experiments that provide evidence that tBID can exert functions similar (but not exactly identical) to the functions of BAX and BAK. The data presented are of potential interest, but I recognize several points where the interpretation of the data is an over-reach and where additional experiments are needed to justify the conclusions. If all of the work that I have indicated below will be performed, I believe that a substantially manuscript may be of great interest to the readers of The EMBO Journal.

1) All data in Figure 1 come from studies using tBID-GFP fusioon proteins. What is the effect of GF? e.g. it could change the halflife of tBID and thereby facilitate an effect of tBID that it normaslly does not exert. To justify the conclusions that tBID can function in manner similar to BAX and BAK, the authors need to repeat all studies in Figure 1 and S2 with tBID-IRES-GFP expression vectors where tBID is NOT a fusion protein.

2) Figure 2: In addition to using COMBO (BCL-2/BCL-XL/BCL-W + MCL-1 inhibitors) this experiment must be performed in cells rendered deficient for the pro-survival BCL-2 family members that are expressed in these cells that have already been rendered BAX/BAK double knockout, probably BCL-XL and MCL-1 and maybe also BCL-2.

3) Figure 2: Emricasan inhibits not only caspase-8 but also additional caspases. Therefore this conclusion is an over-reach. The authors should generate caspase-8/Mlkl double knockout cell lines (already lacking BAX and BAK) to prove this point.

4) Figure 2K: Again to justify the conclusions of the authors, this experiment should also be conducted in cell that are BAX/BAK double knockout that are also rendered deficient for the pro-survival BCL-2 proteins that they normally express, in addition to using COMBO treatment.

5) Figure 3: As above (comments about data in Figure 1), there are potential issues with using fusion proteins of tBID (and variants therefor) to GFP. These experiments need to be complemented by experiments in which they express untagged tBID (and variants thereof) with GFP after an IRES as a marker.

6) Figure 4: Again (see comments about Figures 1 and 3), these experiments need to also be performed with tBID not as a fusion protein to GFP but tBID alone, using IRES GFP as a marker.

7) Figure 6: To properly justify the conclusions of the authors,this study needs to also be conducted by taking parental Nalm6 cells and rendeing them BAX/BAK double deficient or BAX/BAK/BID triple deficient to rule out that the mutant BAK may have a function.
The authors also need to generate Nalm6 cells deficient for BAX, BAK and BOK to rule out a possible role for BOK in this form of cell killing

Referee #2:

Manuscript by Flores-Romero and colleagues describes a novel function of Bcl-2 pro-apoptotic protein BID. The authors claim that processed/truncated form of BID, tBID can act as a BAX-like effector of apoptosis in cancer cells. Furthermore, the authors show that tBID can promote mitochondrial permeabilization to facilitate the leakage of Smac and cytochrome C and induction of apoptosis.

Major points:
- In figure 1, the authors should test all other BH3 proteins for their ability to induce MOMP, not just BID and BIM to verify this is not a common BH3 feature.
- The mutations that have been identified in cancer only showed altered cell death inducing potential in AKO cells. How many cancers display a lack of all pro-survival Bcl-2 family members? This question also seems important concerning the relevance of tBID as it appears to be a mediator of MOMP only when Bcl-2 family members are inhibited/deleted.
- Concerning the data generated for BID KO MEFs: What is the amount of cell death induced compared to a GFP transfected cells?
- In figure 5, the authors should generate BAX/BAK DKO HeLa cells to confirm the findings obtained with HCT DKO cells.

Specific points:
• Confirmation of expression levels of all the proteins of interest for Fig.1A
• Fig. 1C, S1B, 2D, 6F, 6G: Loading controls need to be added to the Western Blots. In case of fractionation it would be good to have a loading control at least for the soluble/ cytosolic fraction.
• Fig. 1E: The quality if the Western Blot is poor. The WB should be repeated and arrows should indicate the band for cleaved PARP.
• Compare Fig. 1D and S1D
• S1E: It would be great if the authors could add the MOCK cells.
• Indicate the time of S1F, G transfection in relation to the treatment.
• S2A: Poor quality Western Blot for BOK. It would be good to show a longer exposure to confirm deletion properly.
• Figure 3A is not cited in the text.
• 6C: Can the authors provide another method to confirm the KO (sequencing?), the WB indicates residual protein or unspecific band?
• Some of the graphs contain data points that are shown in white, which makes it hard to see them. The authors should reformate the graphs to allow better visibility of the graphs.

Comments for the Editor
This is relatively novel and interesting manuscript, but the authors should address all listed comments before re-evaluation.

Referee #3:
Manuscript # EMBOJ-2021-108690

General Remarks
This study looks at how the pro-apoptotic bcl2 family member, BID, causes apoptosis. In Figure 1, the authors use cells devoid of essentially all bcl2 family members to show that tBID over expression is sufficient to promote apoptosis. In Fig. 2, they show that endogenous tBID, provided pro-survival bcl2 family members are inhibited, is also able to induce apoptosis without the need for BAX/BAK. In Fig. 3, by expressing different mutants of BID they show that this apoptotic activity requires the core bcl2 like structure and not the BH3 (which normally inactivates pro-survival bcl2 family members). In Fig. 4, using Electron and STED Microscopy they show that while tBID can induce disruption of the mitochondrial membrane it does not do so by forming the large oligomers demonstrated for BAX/BAK. The authors than go onto the explore the ramifications of their findings: in Fig. 5 they follow up some of their earlier findings that SMAC release, following Shigella infection, which is BAX/BAK independent, can be driven by tBID, and finally in Fig. 6, they show that a cell line resistant to venetoclax because of loss of BAX/mutation in BAK, can be killed by TRAIL in a BID dependent manner.

I think the primary observations are striking, interesting to the field and difficult to account for in any other way than the interpretation proposed by the authors themselves. I find some of the later data difficult to square with the authors hypothesis but before clarifying my difficulties I wish to be clear that just because I don't understand doesn't mean that I wish to hold up publication.

1. The authors show that tBID is sufficient to promote efficient cytochrome c release into the cytoplasm and even mtDNA herniation. I find those observations difficult to square with the observations that tBID forms only limited numbers of breaches (Electron Microscopy) and without the massive oligomerization observed with BAX/BAK. The authors make a reasonable point that it's not proven that BAX/BAK oligomerization and large pore formation are required for these events ... but mtDNA herniation?

2. While I genuinely appreciate the fact that they have extended their earlier findings in Shigella infection by showing that Bax/Bak are not required and that BID alone is sufficient for SMAC release, this still leaves too? many questions open. It seems

superficially attractive that BID forms a different quality of pore to BAX/BAK but, again referring back to the data in this manuscript, it still doesn't explain why SMAC which is a lot larger than cytochrome c is released while cytochrome c is not.

Specific Remarks
While the quality of the data overall is high some of the Western blots look amateurish, the most egregious example is Fig. 1E, but there are a couple of others that I personally would be dissatisfied with.

Fig. 3 I am not a bcl2 family member expert, but does the data in Fig. 3 shed any light on why BID must be cleaved to become activated. Does the increased killing of the M97A/D98A mutant suggest that the BH3 of BID is inhibiting its pore forming killing activity?

Page 6 synergetic should be synergistic.

Fig. 6A, B why switch to caspase activity, when previous assays directly measured cell death ?
Fig. 6F. Which band is cleaved BID? Whjy have a panel with increasing levels of TRAIL when the reader is not informed what the dose used in the rest of the panels is in the figure legend? If the upper band is uncleaved BID, isn't it a little surprising that such small amounts of cleaved BID are able to cause cell death?

POINT-BY-POINT ANSWER TO REVIEWERS

Referee #1:

BID is widely considered to be the precursor a pro-apoptotic BH3-only protein, that can for example be activated by caspase-8 mediated proteolysis in cells treated with agonists of death receptors (e.g. Fas ligand, TNF or TRAIL). However, it has long been known that the structure of BID closely resembles the structures of BAX, BAK (and BOK). This may indicate that BID, like BAX and BAK, could function as multi-BH domain effector of apoptosis, and not only as the precursor for tBID.
In this manuscript, Hector Flores-Romero and colleagues present data from biochemical and cell biology experiments that provide evidence that tBID can exert functions similar (but not exactly identical) to the functions of BAX and BAK. The data presented are of potential interest, but I recognize several points where the interpretation of the data is an over-reach and where additional experiments are needed to justify the conclusions. If all of the work that I have indicated below will be performed, I believe that a substantially manuscript may be of great interest to the readers of The EMBO Journal.

We thank the reviewer for the positive review and for acknowledging the interest of our work.

1) All data in Figure 1 come from studies using tBID-GFP fusion proteins. What is the effect of GFP? e.g. it could change the halflife of tBID and thereby facilitate an effect of tBID that it normaslly does not exert. To justify the conclusions that tBID can function in manner similar to BAX and BAK, the authors need to repeat all studies in Figure 1 and S2 with tBID-IRES-GFP expression vectors where tBID is NOT a fusion protein.

We thank the reviewer for this suggestion. We have now confirmed the apoptosis-inducing activity of tBID using a tBID-IRES-GFP construct. In the new Appendix Figure S1, we show how tBID-IRES-GFP induces apoptosis, highly comparable to tBID-GFP, in HCT allKO cells. This has been confirmed with assays of cyt c release and PARP cleavage by Western blot, activation of caspases, as well as enumerating pyknotic nuclei by confocal microscopy.

2) Figure 2: In addition to using COMBO (BCL-2/BCL-XL/BCL-W + MCL-1 inhibitors) this experiment must be performed in cells rendered deficient for the pro-survival BCL-2 family members that are expressed in these cells that have already been rendered BAX/BAK double knockout, probably BCL-XL and MCL-1 and maybe also BCL-2.

We certainly acknowledge the suggestion made by this referee but are uncertain about the underlying rationale. Given the demonstrated selectivity of the BH3-mimetics used for targeting and inhibiting the relevant pro-survival proteins and the predictable effort in generating multiple sequential deletion mutants by CRISPR in HCT-116 DKO cells

we would like to understand the added value envisioned by this referee that may be coming from conducting such an experiment. Next to being extremely time consuming, they will also not be without caveats. Deletion of the suggested three pro-survival BCL2 proteins would not ensure that we have generated a genetic background lacking the entire pro-survival activity of the BCL2 family, as BCLW, BCLB and/or A1/BFL-1 could still be expressed in the cells. While BCLW is targeted also by ABT737, we cannot exclude compensatory upregulation of BCLB or BFL1. As such, to satisfy this reviewer´s recommendation, we would need to conduct the sequential deletion of in total six reportedly anti-apoptotic BCL2 family proteins, which we simply deemed unfeasible given the timeframe for revision. Albeit we appreciate the beauty of such a genetic experiment over pharmacological inhibition, we simple were unable to meet this request, focusing on the remaining suggestions by this and the two other referees. Yet, we are hopeful that the number of additional data provided will be seen favorable by this referee.

3) Figure 2: Emricasan inhibits not only caspase-8 but also additional caspases. Therefore this conclusion is an over-reach. The authors should generate caspase-8/Mlkl double knockout cell lines (already lacking BAX and BAK) to prove this point.

Following the reviewer's suggestion, we have generated caspase 8 deficient BAX BAK DKO cells by knock down and proven that cell death induction is reduced compared to BAX BAK DKO cells. We show this data now in Figure EV3D.

With regard to MLKL, in contrast to mice, it is not necessary to delete it to avoid cell death in human cell lines. HCT116 cells do not express RIPK3 and present very low levels of MLKL, hence resisting C8 depletion, suggesting that the necroptosis pathway is impaired (for example, Yang et al, 2017, CDDis. e3084).

4) Figure 2K: Again to justify the conclusions of the authors, this experiment should also be conducted in cell that are BAX/BAK double knockout that are also rendered deficient for the pro-survival BCL-2 proteins that they normally express, in addition to using COMBO treatment.

See response to point 2 above.

5) Figure 3: As above (comments about data in Figure 1), there are potential issues with using fusion proteins of tBID (and variants therefor) to GFP. These experiments need to be complemented by experiments in which they express untagged tBID (and variants thereof) with GFP after an IRES as a marker.

Following the reviewer's suggestion, we have now repeated this experiment using a tBID-IRES-construct, which is now shown in Figure EV4C-D. For the case of the mutations found in cancer patients, we have confirmed a selection of the mutants due to time constrains.

6) Figure 4: Again (see comments about Figures 1 and 3), these experiments need to also be performed with tBID not as a fusion protein to GFP but tBID alone, using IRES GFP as a marker.

Following the reviewer's suggestion, we have now repeated this experiment using a tBID-IRES-construct, which is now shown in Appendix Figure S3A.

7) Figure 6: To properly justify the conclusions of the authors,this study needs to also be conducted by taking parental Nalm6 cells and rendeing them BAX/BAK double deficient or BAX/BAK/BID triple deficient to rule out that the mutant BAK may have a function.

The authors also need to generate Nalm6 cells deficient for BAX, BAK and BOK to rule out a possible role for BOK in this form of cell killing.

Following the reviewer's suggestion, we have tested the effect of BAK and BOK deficiency in the Nalm6 cells. Since the parental and the venetoclax resistant cells may not behave in exactly the same way, we have generated a BAK KO and a BOK KO mutant cell line in the venetoclax resistant R199 background. In these experiments, which are now presented in Figure 6I, we show that, in contrast to BID KO, deletion of BAK or of BOK alone in Nalm6 R199 does not decrease TRAIL-induced cell death.

Due to time constrains, we performed the deletions using bulk CRIPR/Cas9, where we selected transfected cells by Venus expression. The Western blot showing BAK depletion is in Figure EV5E. Unfortunately, we are unable to detect BOK levels by Western blot in Nalm6 cells, because they are naturally very low. As a result, we do not have the possibility of confirming that BOK has been deleted, but in any case, the expression levels are undetectable and should not have a significant contribution. We have explained this in the corresponding figure caption.

Referee #2:

Manuscript by Flores-Romero and colleagues describes a novel function of Bcl-2 pro-apoptotic protein BID. The authors claim that processed/truncated form of BID, tBID can act as a BAX-like effector of apoptosis in cancer cells. Furthermore, the authors show that tBID can promote mitochondrial permeabilization to facilitate the leakage of Smac and cytochrome C and induction of apoptosis.

Major points:

- In figure 1, the authors should test all other BH3 proteins for their ability to induce MOMP, not just BID and BIM to verify this is not a common BH3 feature.

We thank the reviewer for this comment. We have now tested the cell death inducing activity of all other BH3-only proteins in the HCT all KO cells, which is shown in Figure EV1C. These results show that other BH3-only proteins do not significantly induce cell death.

- The mutations that have been identified in cancer only showed altered cell death inducing potential in AKO cells. How many cancers display a lack of all pro-survival Bcl-2 family members? This question also seems important concerning the relevance of tBID as it appears to be a mediator of MOMP only when Bcl-2 family members are inhibited/deleted.

Generally, apoptosis resistance is a hallmark of cancer, usually associated with pro-survival BCL-2 overexpression. Nevertheless, it has been shown that in many cancers pro-survival BCL-2 are blocked by BH3-only proteins, thereby rendering the cells primed for apoptosis induction. In this scenario, inactivation of the direct MOMP-inducing activity of tBID could be relevant for cancer cell survival. Our data with the venetoclax resistant Nalm6 R199 cells support this idea.

- Concerning the data generated for BID KO MEFs: What is the amount of cell death induced compared to a GFP transfected cells?

Following the reviewer's suggestion, we have now included the cell death induced by GFP transfection in Figure 3E. Overexpression of tBID-GFP in BID KO MEFs induces around 18,3 +/- 3,1 % cell death significantly higher than expression of GFP alone (9,2 +/- 1,9 %).

- In figure 5, the authors should generate BAX/BAK DKO HeLa cells to confirm the findings obtained with HCT DKO cells.

Following the reviewer's suggestion, we have now performed this experiment in BAX BAK DKO HeLa cells, which we have included in Figure 5D.

Specific points:

• Confirmation of expression levels of all the proteins of interest for Fig.1A

Following the reviewer's suggestion, the expression levels of proteins in Figure 1A is now shown in Figure EV1B.

• Fig. 1C, S1B, 2D, 6F, 6G: Loading controls need to be added to the Western Blots. In case of fractionation it would be good to have a loading control at least for the soluble/ cytosolic fraction.

Following the reviewer's suggestion, we have included the loading control with GAPDH and TOM20 for the subcellular fractionations in EV1B and ponceau staining in Figure 1C. For Figures 2D and 6F-G, due to time constrains, we keep the presentation of the loading controls with ponceau staining, which is widely accepted in the community and even explicitly recommended by some journals, like Journal of Biological Chemistry (https://doi.org/10.1074/jbc.E115.000002).

• Fig. 1E: The quality if the Western Blot is poor. The WB should be repeated and arrows should indicate the band for cleaved PARP.

We apologize for this and present a better Western blot in the revised version.

• Compare Fig. 1D and S1D

In Figure 1D we have performed a controlled time series of caspase activity to show that the activation of caspases increases over time for cells overexpressing GFP-BAX, GFP-BAK and tBID-GFP, but not GFP-BCL-xL. In Figure EV1D, we rather wanted to show that tBID without GFP also was similarly capable of activating caspases, but not full-length BID-GFP at a single time point. Here we use tBID-GFP and GFP-BAX as references. The average values are quite similar if not the same.

• S1E: It would be great if the authors could add the MOCK cells.

We have included them in the revised version, now EV1G.

• Indicate the time of S1F, G transfection in relation to the treatment.

We have now included this information in the figure caption (EV1F,G).

• S2A: Poor quality Western Blot for BOK. It would be good to show a longer exposure to confirm deletion properly.

We have now substituted for a better Western blot (Figure EV2A).

• Figure 3A is not cited in the text.

We have now corrected this.

• 6C: Can the authors provide another method to confirm the KO (sequencing?), the WB indicates residual protein or unspecific band?

The WB indicates low residual protein in agreement with the fact that the depletion was done by bulk CRISP/Cas9 genome editing and a small fraction of the cellular population may still express BID.

• Some of the graphs contain data points that are shown in white, which makes it hard to see them. The authors should reformate the graphs to allow better visibility of the graphs.

We have now corrected this.

Comments for the Editor

This is relatively novel and interesting manuscript, but the authors should address all listed comments before re-evaluation.

We thank the reviewer for acknowledging the novelty and interest of our work.

Referee #3:

Manuscript # EMBOJ-2021-108690

General Remarks

This study looks at how the pro-apoptotic bcl2 family member, BID, causes apoptosis. In Figure 1, the authors use cells devoid of essentially all bcl2 family members to show that tBID over expression is sufficient to promote apoptosis. In Fig. 2, they show that endogenous tBID, provided pro-survival bcl2 family members are inhibited, is also able to induce apoptosis without the need for BAX/BAK. In Fig. 3, by expressing different mutants of BID they show that this apoptotic activity requires the core bcl2 like structure and not the BH3 (which normally inactivates pro-survival bcl2 family members). In Fig. 4, using Electron and STED Microscopy they show that while tBID can induce disruption of the mitochondrial membrane it does not do so by forming the large oligomers demonstrated for BAX/BAK. The authors than go onto the explore the ramifications of their findings: in Fig. 5 they follow up some of their earlier findings that SMAC release, following Shigella infection, which is BAX/BAK independent, can be driven by tBID, and finally in Fig. 6, they show that a cell line resistant to venetoclax because of loss of BAX/mutation in BAK, can be killed by TRAIL in a BID dependent

manner.

I think the primary observations are striking, interesting to the field and difficult to account for in any other way than the interpretation proposed by the authors themselves. I find some of the later data difficult to square with the authors hypothesis but before clarifying my difficulties I wish to be clear that just because I don't understand doesn't mean that I wish to hold up publication.

We thank the reviewer for acknowledging the interest and novelty of our work.

1. The authors show that tBID is sufficient to promote efficient cytochrome c release into the cytoplasm and even mtDNA herniation. I find those observations difficult to square with the observations that tBID forms only limited numbers of breaches (Electron Microscopy) and without the massive oligomerization observed with BAX/BAK. The authors make a reasonable point that it's not proven that BAX/BAK oligomerization and large pore formation are required for these events ... but mtDNA herniation?

When we performed this experiment, we actually expected that tBID would not be able to release mtDNA, as it would be easier to explain considering its lower tendency to oligomerize. But we clearly observed mtDNA release in a very robust manner.

Unfortunately, the molecular mechanism how mtDNA is released from mitochondria during apoptosis is not well understood yet and we do not have a proven explanation of how tBID may mediate this. We can envision that alteration of the inner membrane structure and the cristae can disrupt the proton gradient, leading to matrix swelling, which would exert pressure on the outer mitochondrial membrane leading to bigger ruptures as the observed by EM and to mtDNA release. In the case of tBID, this process would be less efficient than for BAX and BAK, but still occur. But at the moment, this hypothesis is speculation and we have decided not to include it in the discussion because it goes off-topic from the main message of the study.

2. While I genuinely appreciate the fact that they have extended their earlier findings in Shigella infection by showing that Bax/Bak are not required and that BID alone is sufficient for SMAC release, this still leaves too? many questions open. It seems superficially attractive that BID forms a different quality of pore to BAX/BAK but, again referring back to the data in this manuscript, it still doesn't explain why SMAC which is a lot larger than cytochrome c is released while cytochrome c is not.

As published in Andree et al., 2014, EMBO J 33:2171, Sighella infection maintains the mitochondrial structure and function, which would keep cytochrome c engaged in the electron transport chain, and thereby more bound to the inner mitochondrial membrane. As a result, it is released much less efficiently than SMAC once the

permeability barrier of the outer mitochondrial membrane is compromised. We have explained this better in the text (page 10).

Specific Remarks

While the quality of the data overall is high some of the Western blots look amateurish, the most egregious example is Fig. 1E, but there are a couple of others that I personally would be dissatisfied with.

We have now changed several Western blots, including Figure 1E, in an attempt to improve their quality.

Fig. 3 I am not a bcl2 family member expert, but does the data in Fig. 3 shed any light on why BID must be cleaved to become activated.

According to the current knowledge in the field, the cleavage of BID is necessary to release the inhibitory N-terminus segment and the mutants in Figure 3 do not add to this view.

Does the increased killing of the M97A/D98A mutant suggest that the BH3 of BID is inhibiting its pore forming killing activity?

Unfortunately, we cannot make this conclusion because other mutations in the BH3 of BID do not have the same effect. The observed difference could also be mediated by other, yet unknown, mechanisms, which we believe are out of the scope of this study.

Page 6 synergetic should be synergistic.

We have corrected this in the text.

Fig. 6A, B why switch to caspase activity, when previous assays directly measured cell death?

The caspase activity assay is one of the several assays that we have used throughout the manuscript to characterize different aspects of tBID induced apoptosis (see Figure 1D). In this case in Figure 6A,B, we chose this assay for the main text because caspase activation is a hallmark of apoptosis, compared to other forms of regulated cell death, and thereby it provides additional specificity to stablish that the treatment induces or not apoptosis. The cell death induction assay with Draq7 is shown in Figure EV5A.

Fig. 6F. Which band is cleaved BID?

Cleaved BID corresponds to the lower band, which appears only after TRAIL treatment.

Whjy have a panel with increasing levels of TRAIL when the reader is not informed what the dose used in the rest of the panels is in the figure legend?

We apologize for this. We have now included the amount of TRAIL used in the experiments shown in the other panels in the figure caption.

If the upper band is uncleaved BID, isn't it a little surprising that such small amounts of cleaved BID are able to cause cell death?

Given the bulk nature of Western blot analysis and the single cell death analysis carried out with the Incucyte, we think that cleaved BID appears only in a fraction of the cells, which are the ones that undergo cell death. This population typically oscillated between 20-30%, see figure EV5D.

1st Revision - Editorial Decision 29th Oct 2021

Dear Ana,

Thank you for submitting your revised manuscript to The EMBO Journal. I am sorry for the slight delay in getting back to you with a decision, but I have now heard back from the referees and their comments are provided below.

As you can see below there are several issues that need to be resolved. We need better quality western blots. Please take a careful look at the blots and make sure that the quality is OK.

Do you have data on the half-lives for tBID vs tBID-GFP fusion proteins?

Regarding the use of genetic knock outs (ref #1): I see the point that the referee is making, but also considering the input of referee #3 regarding this it I find that it is OK to go without these experiments. If you have any data to further strengthen this point then please include it in the revised version.

When you submit the revised version will you also please address the following points:

You are missing a Data Availability Section. This is the place to enter accession numbers etc. If no data needs to be deposited in a database, then state This study includes no data deposited in external repositories. Please place the section after the Materials and methods and before Acknowledgements

We are missing the author contributions of Lukas Frenzel.

Regarding the reference list => for articles with more than 10 authors the author list should be cut after 10 authors followed by et al.

Please check that there are figure callouts for Fig 4C, Fig EV4D, Appendix Fig S1 panels

Our publisher has also done their pre-publication check on your manuscript. When you log into the manuscript submission system you will see the file "Data Edited MS file". Please take a look at the word file and the comments regarding the figure legends and respond to the issues.

We encourage the publication of source data, particularly for electrophoretic gels and blots, with the aim of making primary data more accessible and transparent to the reader. It would be great if you could provide me with a PDF file per figure that contains the original, uncropped and unprocessed scans of all or key gels used in the figure? The PDF files should be labeled with the appropriate figure/panel number and should have molecular weight markers; further annotation could be useful but is not essential. The PDF files will be published online with the article as supplementary "Source Data" files.

We include a synopsis of the paper (see http://emboj.embopress.org/). Please provide me with a general summary statement and 3-5 bullet points that capture the key findings of the paper.

We also need a summary figure for the synopsis. The size should be 550 wide by [200-400] high (pixels).

Let me know if we need to discuss any points further

With best wishes

Karin

Karin Dumstrei, PhD
Senior Editor
The EMBO Journal

Guide For Authors: https://www.embopress.org/page/journal/14602075/authorguide

The revision must be submitted online within 90 days; please click on the link below to submit the revision online before 27th Jan 2022.

https://emboj.msubmit.net/cgi-bin/main.plex

Referee #1:

The effects of induced expression of tBID on cell viability are rather small, such as only 2-fold induction of caspar activity over untreated healthy cells. This is not a big effect, making one wonder how relevant the proposed mechanism really is.

No data on protein half-lives for tBID vs tBID-GFP fusion proteins are shown. This is an important control.

The quality of several of the Western blots presented is not very good.

Several of the important experiments that I had requested during the assessment of the original version of this manuscript were not conducted. The added value of the requested experiments would be to be able to show that the proposed killing effects of tBID are independent of all other BCL-2 family members. When pro-survival proteins, such as BCL-2 or MCL-1 are bound by BH3 mimetic drugs they are inhibited in their ability to bind endogenous BH3-only proteins or BAX and BAK, but these proteins are still present and can exert functions. Such functions can only be excluded by doing the experiments that I had proposed.

The impact of loss of caspase-8 is not very large, making one wonder how important the mechanism involving caspase-8 that was proposed by the authors really is.

Referee #2:

The authors have largely addressed reviewers' comments although the quality of some western blot data is still questionable (levels of GAPDH, which is a loading control, are very uneven in figures EV1 and 2). But this manuscript could be accepted now, maybe with very minor modifications according to my suggestions.

Referee #3:

The authors have done a good job with their response. Re the request for genetic knock-outs from reviewer 1, I am entirely sympathetic with the response of the authors. While it would be "nice" to do the genetic knock outs, the bcl2 family inhibitors are very well characterised, so it's hard to see what this experiment adds conceptually. On the other hand it's easy to see that it adds enormously to the technical challenges and is unreasonable in the time alloted to revisions. In my opinion, the main novelty of the manuscript is that tBid can act to permeabilise mitochondria and unless the reviewer thinks that pro-survival bcl2 are killing (which would be way more noteworthy than tBid and a major paper in its own right), I, like the authors, do not think the cost/benefit is sufficient to justify this experiment.

POINT-BY-POINT ANSWER TO REVIEWERS #2

Referee #1:

A) The effects of induced expression of tBID on cell viability are rather small, such as only 2-fold induction of caspase activity over untreated healthy cells. This is not a big effect, making one wonder how relevant the proposed mechanism really is.

We thank the reviewer for this comment. Importantly, despite the moderate caspase activity in the presence of tBID overexpression (3,39 ±0,35-fold with respect to the basal caspase activity), the caspase activaty that we detect is similar to that elicited by BAX and BAK (4,39 ±0,56 and 5,10 ±0,83, respectively, at 24 h)(Fig 1D), with no significant difference among them. In contrast, the overexpression of GFP or GFP-BCL-XL did not produce an increase of caspase activity.

B) No data on protein half-lives for tBID vs tBID-GFP fusion proteins are shown. This is an important control.

While we agree with the reviewer that this would be an important control in the original submission, we believe that the additional experiments provided in the revised version already control for the possibility that the effects of tBid reported here are due to an altered half-life due to the GFP fusion. Concretely, we provide several assays in which non-tagged tBID (both tBID and tBID-IRES-GFP) produced similar amounts of cell death hallmarks compared to tBID-GFP (Fig 1C,D, EV1 E,F and appendix S1A-E).

C) The quality of several of the Western blots presented is not very good.

We thank the reviewer for pointing this out. Please, see answer to reviewer 2.

D) Several of the important experiments that I had requested during the assessment of the original version of this manuscript were not conducted. The added value of the requested experiments would be to be able to show that the proposed killing effects of tBID are independent of all other BCL-2 family members. When pro-survival proteins, such as BCL-2 or MCL-1 are bound by BH3 mimetic drugs they are inhibited in their ability to bind endogenous BH3-only proteins or BAX and BAK, but these proteins are still present and can exert functions. Such functions can only be excluded by doing the experiments that I had proposed.

We certainly acknowledge the suggestion made by this referee. Indeed, the concern of the reviewer was our rationale for the use of HCT AKO cells, where the moderate but still significant killing activity of tBID overexpression takes place in absence of other BCL-2 family members. Furthermore, in the revised version we showed that in this cellular system we did not detect induction of cell death by any other BH3-only protein nor by the antiapoptotic family members individually. Considering the non-observable killing activity of these BCL-2 proteins on their own, we understand that the analysis of tBID activity in this putative cell line suggested by the reviewer would reassemble the one done in HCT AKO cells. Thus, albeit we appreciate the suggestion, we simply were unable to meet this request due to time limitations, focusing on the remaining suggestions by this and the two other referees. Yet, we are hopeful that the number of additional data provided will be seen favorably by this referee.

E) The impact of loss of caspase-8 is not very large, making one wonder how important the mechanism involving caspase-8 that was proposed by the authors really is.

We agree with the reviewer that the effect of caspase-8 silencing is moderate. This is probably related to the relatively low cell death activity induced by endogenous tBID. However, we want to emphasize that we observe a significant reduction in cell death activity when casp8 is KD with respect to the siRNA control.

Referee #2:

The authors have largely addressed reviewers' comments although the quality of some western blot data is still questionable (levels of GAPDH, which is a loading control, are very uneven in figures EV1 and 2). But this manuscript could be accepted now, maybe with very minor modifications according to my suggestions.

We thank the reviewer for pointing this out. We have now included the ponceau staining as an additional loading control together with reblotted GAPDH for the subcellular fractionation in EV1, where the alike amount of protein per condition is more evident. Regarding EV2, it is very hard to detect BOK and to obtain a strong signal as requested by the reviewers, we had to load the gels as much as possible, which affected the running of the lanes. To solve this issue, we include now the ponceau staining of the gel as well as another replica of the experiment containing less amount of cell extracts per lane, where the loading controls look fine.

Referee #3:

The authors have done a good job with their response. Re the request for genetic knock-outs from reviewer 1, I am entirely sympathetic with the response of the authors. While it would be "nice" to do the genetic knock outs, the bcl2 family inhibitors are very well characterised, so it's hard to see what this experiment adds conceptually. On the other hand it's easy to see that it adds enormously to the technical challenges and is unreasonable in the time allowed to revisions. In my opinion, the main novelty of the manuscript is that tBid can act to permeabilise mitochondria and unless the reviewer thinks that pro-survival bcl2 are killing (which would be way more noteworthy than tBid and a major paper in its own right), I, like the authors, do not think the cost/benefit is sufficient to justify this experiment.

We thank the reviewer for acknowledging the effort done to improve the quality of our manuscript.

2nd Revision - Editorial Decision 22nd Nov 2021

Dear Ana,

Thank you for submitting your manuscript to The EMBO Journal. I have now had a chance to take a look at the revised version and I appreciate the introduced changes.

I am therefore very pleased to accept the manuscript for publication here.

With best wishes

Karin

Karin Dumstrei, PhD
Senior Editor
The EMBO Journal

Please note that it is EMBO Journal policy for the transcript of the editorial process (containing referee reports and your response letter) to be published as an online supplement to each paper. If you do NOT want this, you will need to inform the Editorial Office via email immediately. More information is available here: https://www.embopress.org/page/journal/14602075/authorguide#transparentprocess

Your manuscript will be processed for publication in the journal by EMBO Press. Manuscripts in the PDF and electronic editions of The EMBO Journal will be copy edited, and you will be provided with page proofs prior to publication. Please note that supplementary information is not included in the proofs.

Please note that you will be contacted by Wiley Author Services to complete licensing and payment information. The 'Page Charges Authorization Form' is available here: https://www.embopress.org/pb-assets/embo-site/tej_apc.pdf

Should you be planning a Press Release on your article, please get in contact with embojournal@wiley.com as early as possible, in order to coordinate publication and release dates.

If you have any questions, please do not hesitate to call or email the Editorial Office. Thank you for your contribution to The EMBO Journal.

** Click here to be directed to your login page: https://emboj.msubmit.net

EMBO PRESS

YOU MUST COMPLETE ALL CELLS WITH A PINK BACKGROUND ↓

PLEASE NOTE THAT THIS CHECKLIST WILL BE PUBLISHED ALONGSIDE YOUR PAPER

Corresponding Author Name:	Ana J. Garcia-Saez
Journal Submitted to:	The EMBO journal
Manuscript Number:	Manuscript EMBOJ-2021-108690

USEFUL LINKS FOR COMPLETING THIS FORM

http://www.antibodypedia.com
http://1degreebio.org
http://www.equator-network.org/reporting-guidelines/improving-bioscience-research-repor
http://grants.nih.gov/grants/olaw/olaw.htm
http://www.mrc.ac.uk/Ourresearch/Ethicsresearchguidance/Useofanimals/index.htm
http://ClinicalTrials.gov
http://www.consort-statement.org
http://www.consort-statement.org/checklists/view/32-consort/66-title
http://www.equator-network.org/reporting-guidelines/reporting-recommendations-for-tumo
http://datadryad.org
http://figshare.com
http://www.ncbi.nlm.nih.gov/gap
http://www.ebi.ac.uk/ega
http://biomodels.net/
http://biomodels.net/miriam/
http://jjj.biochem.sun.ac.za
https://osp.od.nih.gov/biosafety-biosecurity-and-emerging-biotechnology/
http://www.selectagents.gov/

Reporting Checklist For Life Sciences Articles (Rev. June 2017)

This checklist is used to ensure good reporting standards and to improve the reproducibility of published results. These guidelines are consistent with the Principles and Guidelines for Reporting Preclinical Research issued by the NIH in 2014. Please follow the journal's authorship guidelines in preparing your manuscript.

A- Figures

1. Data

The data shown in figures should satisfy the following conditions:

- ➔ the data were obtained and processed according to the field's best practice and are presented to reflect the results of the experiments in an accurate and unbiased manner.
- ➔ figure panels include only data points, measurements or observations that can be compared to each other in a scientifically meaningful way.
- ➔ graphs include clearly labeled error bars for independent experiments and sample sizes. Unless justified, error bars should not be shown for technical replicates.
- ➔ if n< 5, the individual data points from each experiment should be plotted and any statistical test employed should be justified
- ➔ Source Data should be included to report the data underlying graphs. Please follow the guidelines set out in the author ship guidelines on Data Presentation.

2. Captions

Each figure caption should contain the following information, for each panel where they are relevant:

- ➔ a specification of the experimental system investigated (eg cell line, species name).
- ➔ the assay(s) and method(s) used to carry out the reported observations and measurements
- ➔ an explicit mention of the biological and chemical entity(ies) that are being measured.
- ➔ an explicit mention of the biological and chemical entity(ies) that are altered/varied/perturbed in a controlled manner.
- ➔ the exact sample size (n) for each experimental group/condition, given as a number, not a range;
- ➔ a description of the sample collection allowing the reader to understand whether the samples represent technical or biological replicates (including how many animals, litters, cultures, etc.).
- ➔ a statement of how many times the experiment shown was independently replicated in the laboratory.
- ➔ definitions of statistical methods and measures:
  - common tests, such as t-test (please specify whether paired vs. unpaired), simple χ2 tests, Wilcoxon and Mann-Whitney tests, can be unambiguously identified by name only, but more complex techniques should be described in the methods section;
  - are tests one-sided or two-sided?
  - are there adjustments for multiple comparisons?
  - exact statistical test results, e.g., P values = x but not P values < x;
  - definition of 'center values' as median or average;
  - definition of error bars as s.d. or s.e.m.

Any descriptions too long for the figure legend should be included in the methods section and/or with the source data.

In the pink boxes below, please ensure that the answers to the following questions are reported in the manuscript itself. Every question should be answered. If the question is not relevant to your research, please write NA (non applicable). We encourage you to include a specific subsection in the methods section for statistics, reagents, animal models and human subjects.

B- Statistics and general methods

Please fill out these boxes ↓ (Do not worry if you cannot see all your text once you press return)

1.a. How was the sample size chosen to ensure adequate power to detect a pre-specified effect size?	The design of the experimental conditions was based on previous knowledge from similar experiments were done in the field (Ma SB et al 2014 in CDD, Usai A et al 2020 in Cancers and others). Also, pilot experiments were carried out that indicated the sample sizes chosen were adequate for a robust phenotype.
1.b. For animal studies, include a statement about sample size estimate even if no statistical methods were used.	NA
2. Describe inclusion/exclusion criteria if samples or animals were excluded from the analysis. Were the criteria pre-established?	NA
3. Were any steps taken to minimize the effects of subjective bias when allocating animals/samples to treatment (e.g. randomization procedure)? If yes, please describe.	NA
For animal studies, include a statement about randomization even if no randomization was used.	NA
4.a. Were any steps taken to minimize the effects of subjective bias during group allocation or/and when assessing results (e.g. blinding of the investigator)? If yes please describe.	Yes. Many experiments were carried out by 2 or more investigators independently and in addition, for imaging experiments the investigators performed a blinded analysis.
4.b. For animal studies, include a statement about blinding even if no blinding was done	NA
5. For every figure, are statistical tests justified as appropriate?	Yes. Where 2 experimental groups are being compared a Student's t-test was performed. When comparing multiple experimental groups, one-way ANOVA was perfomed.
Do the data meet the assumptions of the tests (e.g., normal distribution)? Describe any methods used to assess it.	We have used Sigmaplot11 for the statitiscal analysis within which the calculation of normal distribution and equal variance test are performed. When data do not fit this criteria we perform Mann-whitney Rank sum test or anova on ranks.
Is there an estimate of variation within each group of data?	Yes. The variation within each group of data was estimated by standard deviation.
Is the variance similar between the groups that are being statistically compared?	Yes. The standard deviation of the different samples analysed within the same experiment are in the same order of magnitude.

C- Reagents

6. To show that antibodies were profiled for use in the system under study (assay and species), provide a citation, catalog number and/or clone number, supplementary information or reference to an antibody validation profile. e.g., Antibodypedia (see link list at top right), 1DegreeBio (see link list at top right).	anti-cyt c (BD Biosciences Cat# 556432, RRID:AB_396416); anti-SMAC (Cell Signaling Technology Cat# 15108, RRID:AB_2798711); anti-PARP (Cell Signaling Technology Cat# 9542, RRID:AB_2160739); anti-BAX (Cell Signaling Technology Cat# 2772, RRID:AB_10695870); anti-actin (Cell Signaling Technology Cat# 4967, RRID:AB_330288); anti-GAPDH (Santa Cruz Biotechnology Cat# sc-47724, RRID:AB_627678); anti-BAK (Cell Signaling Technology Cat# 3814, RRID:AB_2290287); anti-BID (Cell Signaling Technology Cat# 2002, RRID:AB_10692485); anti-BOK (Abcam Cat# ab186745, RRID:AB_2728737); anti-α-tubulin (Cell Signaling Technology Cat# 2144, RRID:AB_2210548); anti-VDAC (Cell Signaling Technology Cat# 4661, RRID:AB_10557420); anti-cytochrome c (BD Biosciences Cat# 556433, RRID:AB_396417); anti-GFP (Roche Cat# 11814460001, RRID:AB_390913); anti-BAX 6A7 antibody (BD Biosciences Cat# 556467, RRID:AB_396430); anti-BAK (Millipore Cat# AM03-20UG, RRID:AB_564252); anti-Tom20 (Cell Signaling Technology Cat# 42406, RRID:AB_2687663)
7. Identify the source of cell lines and report if they were recently authenticated (e.g., by STR profiling) and tested for mycoplasma contamination.	HeLa - EMBL Heidelberg; U2OS BAX/BAK DKO - ATCC® HTB-96; MEF BID KO - generated from C57BL/6J BIDKO mice (Andree et al, 2014); HCT WT, HCT BAX/BAK DKO - gifted from Dr Schulze-Osthoff from the University of Tübingen; HCT all BCL-2 KO - generated via CRISPR/Cas9 (O'Neill et al, 2016); HCT BAX/BAK/BID TKO, HCT BCL-2 KO BOK KO, Nalm6 199R BID KO, Nalm6 199R BAK KO, Nalm6 199R BOK KO - generated in-house by CRISPR/Cas9 from previously mentioned parental cell lines.x`

* for all hyperlinks, please see the table at the top right of the document

D- Animal Models

8. Report species, strain, gender, age of animals and genetic modification status where applicable. Please detail housing and husbandry conditions and the source of animals.	NA
9. For experiments involving live vertebrates, include a statement of compliance with ethical regulations and identify the committee(s) approving the experiments.	NA
10. We recommend consulting the ARRIVE guidelines (see link list at top right) (PLoS Biol. 8(6), e1000412, 2010) to ensure that other relevant aspects of animal studies are adequately reported. See author guidelines, under 'Reporting Guidelines'. See also: NIH (see link list at top right) and MRC (see link list at top right) recommendations. Please confirm compliance.	NA

E- Human Subjects

11. Identify the committee(s) approving the study protocol.	NA
12. Include a statement confirming that informed consent was obtained from all subjects and that the experiments conformed to the principles set out in the WMA Declaration of Helsinki and the Department of Health and Human Services Belmont Report.	NA
13. For publication of patient photos, include a statement confirming that consent to publish was obtained.	NA
14. Report any restrictions on the availability (and/or on the use) of human data or samples.	NA
15. Report the clinical trial registration number (at ClinicalTrials.gov or equivalent), where applicable.	NA
16. For phase II and III randomized controlled trials, please refer to the CONSORT flow diagram (see link list at top right) and submit the CONSORT checklist (see link list at top right) with your submission. See author guidelines, under 'Reporting Guidelines'. Please confirm you have submitted this list.	NA
17. For tumor marker prognostic studies, we recommend that you follow the REMARK reporting guidelines (see link list at top right). See author guidelines, under 'Reporting Guidelines'. Please confirm you have followed these guidelines.	NA

F- Data Accessibility

18: Provide a "Data Availability" section at the end of the Materials & Methods, listing the accession codes for data generated in this study and deposited in a public database (e.g. RNA-Seq data: Gene Expression Omnibus GSE39462, Proteomics data: PRIDE PXD000208 etc.) Please refer to our author guidelines for 'Data Deposition'.<br><br>Data deposition in a public repository is mandatory for:<br>a. Protein, DNA and RNA sequences<br>b. Macromolecular structures<br>c. Crystallographic data for small molecules<br>d. Functional genomics data<br>e. Proteomics and molecular interactions	NA
19. Deposition is strongly recommended for any datasets that are central and integral to the study; please consider the journal's data policy. If no structured public repository exists for a given data type, we encourage the provision of datasets in the manuscript as a Supplementary Document (see author guidelines under 'Expanded View' or in unstructured repositories such as Dryad (see link list at top right) or Figshare (see link list at top right).	NA
20. Access to human clinical and genomic datasets should be provided with as few restrictions as possible while respecting ethical obligations to the patients and relevant medical and legal issues. If practically possible and compatible with the individual consent agreement used in the study, such data should be deposited in one of the major public access-controlled repositories such as dbGAP (see link list at top right) or EGA (see link list at top right).	NA
21. Computational models that are central and integral to a study should be shared without restrictions and provided in a machine-readable form. The relevant accession numbers or links should be provided. When possible, standardized format (SBML, CellML) should be used instead of scripts (e.g. MATLAB). Authors are strongly encouraged to follow the MIRIAM guidelines (see link list at top right) and deposit their model in a public database such as Biomodels (see link list at top right) or JWS Online (see link list at top right). If computer source code is provided with the paper, it should be deposited in a public repository or included in supplementary information.	NA

G- Dual use research of concern

22. Could your study fall under dual use research restrictions? Please check biosecurity documents (see link list at top right) and list of select agents and toxins (APHIS/CDC) (see link list at top right). According to our biosecurity guidelines, provide a statement only if it could.	NA
